# Proteomics of resistance to Notch1 inhibition in acute lymphoblastic leukemia reveals targetable kinase signatures

**DOI:** 10.1038/s41467-021-22787-9

**Published:** 2021-05-04

**Authors:** Giulia Franciosa, Jos G. A. Smits, Sonia Minuzzo, Ana Martinez-Val, Stefano Indraccolo, Jesper V. Olsen

**Affiliations:** 1 Proteomics Program, Novo Nordisk Foundation Center for Protein Research, University of Copenhagen, Copenhagen, Denmark; 2 Department of Molecular Developmental Biology, Radboud University, Nijmegen, Netherlands; 3 Department of Surgery, Oncology and Gastroenterology, University of Padova, Padova, Italy; 4 Veneto Institute of Oncology IOV – IRCCS, Padova, Italy

**Keywords:** Proteomics, Mass spectrometry, Acute lymphocytic leukaemia, Cell signalling

## Abstract

Notch1 is a crucial oncogenic driver in T-cell acute lymphoblastic leukemia (T-ALL), making it an attractive therapeutic target. However, the success of targeted therapy using γ-secretase inhibitors (GSIs), small molecules blocking Notch cleavage and subsequent activation, has been limited due to development of resistance, thus restricting its clinical efficacy. Here, we systematically compare GSI resistant and sensitive cell states by quantitative mass spectrometry-based phosphoproteomics, using complementary models of resistance, including T-ALL patient-derived xenografts (PDX) models. Our datasets reveal common mechanisms of GSI resistance, including a distinct kinase signature that involves protein kinase C delta. We demonstrate that the PKC inhibitor sotrastaurin enhances the anti-leukemic activity of GSI in PDX models and completely abrogates the development of acquired GSI resistance in vitro. Overall, we highlight the potential of proteomics to dissect alterations in cellular signaling and identify druggable pathways in cancer.

## Abstract (web summary)

NOTCH1 is a driver of T-cell acute lymphoblastic leukemia that can be inhibited by γ-secretase inhibitors (GSIs), but their clinical efficacy is limited. Here, the authors compare the phosphoproteomes of GSI resistant and sensitive models, and identify potential kinase targets to overcome GSI resistance.

## Introduction

The Notch1 (N1) receptor belongs to the Notch family of transmembrane proteins, which are highly evolutionary conserved in vertebrates, with crucial roles in cell-fate choices^1^. Activation of Notch signaling requires interactions between a DSL (Delta, Serrate, Lag2) ligand, expressed on the surface of a signal-sending cell, and a Notch receptor (Notch1-4 in mammals), expressed on the surface of an opposing cell (signal-receiving cell). The DSL ligands are type-1 transmembrane glycoproteins, and there are five in mammals, designated as either Delta-like (Dll1, Dll3, and Dll4) or Serrate-like (Jagged1 and Jagged2)^2^. Ligand binding induces proteolytic cleavage of the Notch receptor by the γ-secretase protease complex. This results in the release of the Notch intracellular domain (NICD), which migrates to the nucleus and initiates transcription of downstream target genes^3^.

Deregulation of Notch signaling has wide-ranging roles in multiple cancers^4^. Most prominently, activating oncogenic mutations in the N1 gene are found in 75% of T-cell acute lymphoblastic leukemia (T-ALL) cases^5^. T-ALL is an aggressive hematological malignancy caused by oncogenic transformation of early T-cell progenitors and their diffuse infiltration in the bone marrow leading to hematopoietic failure^6^. The prognosis of patients with primary resistant or relapsed T-ALL remains poor despite the introduction of intensive chemotherapy regimens^7^. The high mutation frequency of the N1 gene makes its protein product an attractive therapeutic target. This has led to clinical testing of γ-secretase inhibitors (GSIs) that prevent N1 cleavage and subsequent activation^8^. However, clinical development of GSI-based therapies have largely failed thus far, primarily due to lack of evidence of sustained anti-tumor responses^9^. This indicates that resistance mechanisms may occur, limiting their clinical efficacy.

Despite the advances of precision medicine, the development of resistance to targeted therapies broadly contributes to cancer mortality^10^. Multiple mechanisms are known to cause GSI resistance in T-ALL cells. Loss-of-function mutations affecting the *FBXW7* gene results in impairment of the main E3-ubiquitin ligase implicated in N1-ICD turnover^11^, leading to residual N1 signaling. Notably, Fbxw7 has also been shown to be involved in the degradation of the cMyc transcription factor^12^, known to be the key N1 target gene responsible for N1 leukemogenic potential in T-ALL^13^. Moreover, acquired changes in epigenetic marks can induce alternative cMyc transcriptional upregulation through the chromatin regulator Brd4^14^, which controls an alternative long-range cMyc enhancer^15^. Furthermore, mutational loss of Pten, a phosphoinositide phosphatase that acts as a tumor suppressor by negatively regulating Akt kinase signaling, was originally associated with GSI resistance^16^, but subsequent studies have not been able to confirm that finding^17^.

**Fig. 1:**
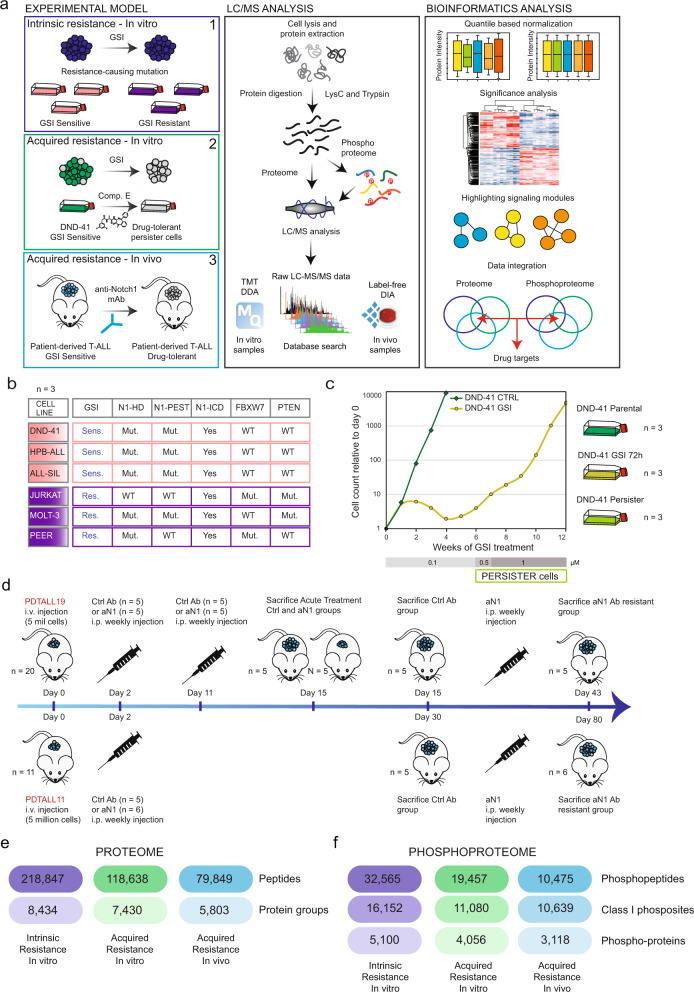
Experimental design and phosphoproteomics workflow for comprehensive analysis of resistance to NOTCHi in T-ALL. **a** Overview of the experimental design and the phosphoproteomics workflow used to study resistance to NOTCHi in T-ALL. **b** T-ALL cell line panel of choice. More information is provided in Supplementary Data 1. **c** Relative live cell count performed by trypan blue exclusion of DND-41 cells treated with an increasing amount of GSI (Compound E) for 12 weeks (left). The experiment was performed once. Schematic representation of three experimental conditions (parental, short-term GSI-treated, and persister DND-41 cells) used to perform the proteomics experiment (right). The three biologically independent samples were collected between week 9 and 11 of treatment. **d** Outline of the treatment with the antiNotch1 monoclonal antibody OMP52M51 or control antibody Rituximab of two T-ALL PDX models (PDTALL11 and PDTALL19) engrafted in NOD/SCID mice. **e**–**f** Overview of results from proteome (E) and phosphoproteome (F) analysis of model-1 (T-ALL cell lines; blue); model-2 (DND-41 acquired resistance; green); model-3 (T-ALL PDX acquired resistance; light blue). LC/MS liquid chromatography mass spectrometry, mAb monoclonal antibody, DDA data-dependent acquisition, DIA data-independent acquisition, Res resistant, Sens sensitive, N1 Notch1, HD heterodimerization domain, PEST PEST domain, ICD intracellular domain, Mut mutated, WT wild type, CTRL DMSO-treated cells, *n* number of biologically independent experiments/mice, aN1 anti-Notch1, RTX Rituximab, i.v. intravenous, i.p. intraperitoneal. Source data are provided as Source Data file.

To explore if intrinsically (driven by genetic mutations) and acquired (driven by non-genetic mechanisms) resistant T-ALL cells share common molecular signatures, we analyzed three complementary in vitro and in vivo models of resistance to Notch inhibition (NOTCHi) by high-resolution liquid chromatography-tandem mass spectrometry (LC-MS/MS)-based proteomics, with the aim of identifying common mediators of resistance (Fig. 1a).

## Results

### A quantitative proteomics approach to define shared mechanisms of resistance to NOTCHi in T-ALL

To characterize intrinsic GSI resistance, we analyzed a panel of six T-ALL cell lines (Model n.1; Fig. 1b). DND-41, HBP-ALL, and ALL-SIL are known to be sensitive to NOTCHi, whereas JURKAT, MOLT-3, and PEER are intrinsically resistant^16^. The cell lines in each group were chosen based on their genetic heterogeneity to best mimic cancer diversity (Supplementary Data 1). For example, within the resistant group, JURKAT and MOLT-3 harbor *PTEN*-null mutations, while JURKAT and PEER carry *FBXW7* mutations, both having been previously linked to GSI resistance^11,16^. Analysis of cell survival after treatment with the GSI XXI, Compound E, showed that the GSI resistant group tolerated an almost 1000-fold higher half-maximal inhibitory concentration (IC50) on average compared to the sensitive group (Supplementary Fig. 1a). The observed differential response was not due to the lack of activity of the drug on N1 cleavage in the resistant cells (Supplementary Fig. 1b). The sensitive cell lines DND-41 and HBP-ALL also showed a cell cycle arrest in the G0/G1 phase, while cell cycle progression was unaffected in the sensitive cell line ALL-SIL and all three resistant cell lines (Supplementary Fig. 1c, d). We further validated ALL-SIL sensitivity by long-term treatment with GSI up to 9 weeks during which no recovery of cell growth was observed (Supplementary Fig. 1e).

As in vitro model of acquired GSI resistance, we isolated a persister cell population from the sensitive cell line DND-41, after 5–6 weeks of treatment with increasing GSI doses (Model n.2; Fig. 1c)^14^. The acquired resistance of persister cells to NOTCHi was confirmed by an almost 1000-fold higher GSI IC50 value than the parental cells (Supplementary Fig. 2a). We included a short-term GSI treatment (72 h) condition to discern between transient and long-term effects of N1 inhibition (Fig. 1c). We validated N1ICD and cMyc protein expression by immunoblotting and confirmed what was previously reported^14^: N1ICD was completely absent in both short and long-term GSI-treated cells, while the N1 target cMyc was rescued in persister cells (Supplementary Fig. 2b).

To reproduce the development of acquired resistance in patients, we treated two Notch-addicted, Notch1-mutated T-ALL patient-derived xenografts (PDX) (PDTALL11 and PDTALL19^18^; Supplementary Data 1), after systemic engraftment in NOD/SCID mice, with the Notch1-specific (aNotch1) neutralizing monoclonal antibody OMP52M51, until leukemia occurrence^19^ (Model n.3). Mice were treated with OMP52M51 or the control anti-Cd20 antibody Rituximab (RTX) once a week. Resistance to OMP52M51 treatment appeared with different kinetics in the two PDX analyzed (after 43 days of PDTALL19 and 80 days of PDTALL11), as previously described^19^. In both models, resistant cells showed downregulation of Notch1 target gene expression and full absence of N1ICD protein expression^19^. T-ALL cells for MS analysis were obtained from the splenocyte population (Fig. 1d).

To investigate differential signaling between sensitive and resistant T-ALL cell lines on a global scale, we performed quantitative mass spectrometry (MS)-based proteomics analysis of two signaling layers: proteome and phosphoproteome (Fig. 1a). We employed two different proteomics strategies across the different experiments (Supplementary Fig. 3). For models 1–2, we used an isobaric labeling-based workflow combined with LC-MS/MS on a Q-Exactive HF-X mass spectrometer^20^. Three biological replicates were collected per condition (Supplementary Figs. 1f, g, 2c). For model n.3, we utilized a single-shot label-free approach in data-independent-acquisition mode without using spectral libraries (direct-DIA) on an Orbitrap Exploris 480^21^ in combination with short LC gradients using the Evosep One system^22^. Five-six mice were allocated per group. In dataset n.1, we quantified 218,847 peptides on 8434 protein groups, 32,565 phosphopeptides on 5100 phosphoproteins and 16,152 localized (class I)^23^ phosphosites. In dataset n.2, we quantified 118,638 peptides on 7430 protein groups, 19,457 phosphopeptides on 4056 phosphoproteins and 11,080 class I phosphosites. In dataset n.3, we quantified 79,849 peptides on 5803 protein groups and 10,639 class I phosphosites corresponding to 10,475 phosphopeptides and 3118 phosphoproteins (Fig. 1e).

### Intrinsic resistance to NOTCHi might be reverted by blocking protein synthesis

**Fig. 2:**
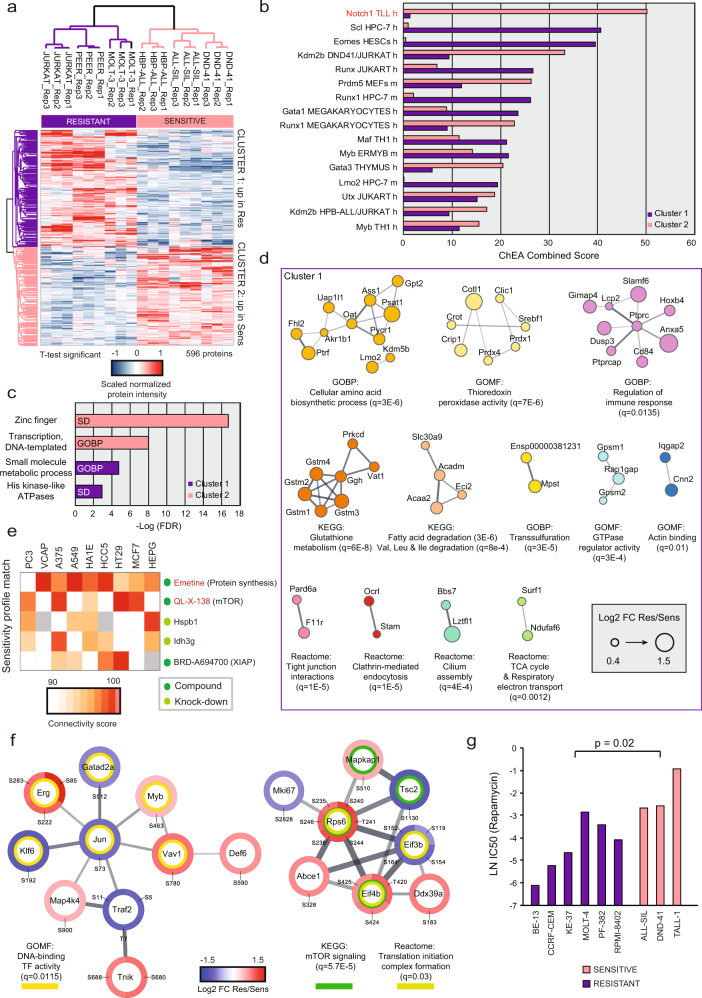
Global proteome signature of intrinsically GSI resistant cells. **a** Hierarchical clustering of normalized protein intensities scaled per replicate (columns: Pearson correlation; rows: Canberra distance) for significantly regulated proteins between resistant and sensitive cell lines (unpaired two-sided SAM test: permutation-based FDR < 0.05; s0 = 0.1; *n* = 9 biologically independent samples examined over three independent experiments). **b** ChIP enrichment analysis (ChEA) performed through Enrichr of all proteins belonging to Cluster1 or Cluster 2. The combined score merges the result of a Fisher exact test and of Z-score in one value. High combined score indicates increased transcriptional activity. Only transcription factors with Benjamini–Hochberg-corrected *p* value < 0.05 are included. **c** Gene ontology enrichment analysis performed through the stringApp (FDR: Benjamini–Hochberg-corrected *p* value). Background: proteome. **d** Functional STRING network of the proteins significantly more abundant in GSI resistant cell lines (permutation-based FDR < 0.01), created through the stringApp. Clusters were generated by MCL clustering through the Clustermaker2 app. Gene ontology enrichment analysis was performed through the stringApp (background: proteome). q: Benjamini–Hochberg-corrected *p* value. **e** Connectivity map analysis of the top-300 differentially regulated proteins between GSI resistant and sensitive cell lines (permutation-based FDR < 0.05), ranked by t-Test statistic (150 proteins on each side have been queried). The connectivity score was calculated using Kolmogorov-Smirnov two-sided statistics implemented in CLUE. A positive score indicates similarity between a given perturbagen’s signature and that of GSI sensitivity. **f** Functional STRING network of phosphoproteins whose phosphorylation is significantly more abundant in resistant cell lines (unpaired two-sided SAM test: permutation-based FDR < 0.01; s0 = 0.1; *n* = 9 biologically independent samples), generated through the stringApp. Clusters were generated by MCL clustering through Clustermaker2. Gene ontology enrichment analysis was performed through the stringApp. Only the two biggest clusters are shown. Background: phosphoproteome. **g** Sensitivity values (natural logarithm, LN, of the half-maximal inhibitory concentration, IC50, in µM) for 9 human T-ALL cell lines treated with the mTOR inhibitor rapamycin. Data were obtained from the Genomics of Drug Sensitivity in Cancer project. An unpaired two-sided *t* test was used to assess the differences in mean LN (IC50) between the GSI resistant (*n* = 6) and sensitive (*n* = 3) cell lines. Res resistant, Sens sensitive, h human, m murine, GOBP gene ontology biological processes, SD smart domain, FC fold-change. Source data are provided as Source Data file.

To evaluate if the response to GSI was evident on the proteome level, we performed a principal component analysis (PCA) after Analysis of Variance (ANOVA) statistical test among the different cell lines (1122 regulated proteins; Supplementary Fig. 4a). This analysis revealed that the different cell line proteomes separated on component-1 in two clearly distinct groups reflecting their response to GSI. Next, we sought to identify the differentially regulated proteins among sensitive and resistant cell line groups by performing a Significance Analysis of Microarrays (SAM) statistical test^24^. We found 596 regulated proteins: 323 were significantly more abundant in the resistant cell line group (cluster 1, C1) and 273 proteins we upregulated in the sensitive cell line group (cluster 2, C2) (Fig. 2a; Supplementary Data 2). To determine whether a particular transcription factor was the master regulator of the proteins more abundant in either sensitive or resistant cells, we performed a transcription factor enrichment analysis using the ChIP enrichment analysis (ChEA) database^25,26^ (Fig. 2b). N1 was found to be the most significantly enriched transcription factor in C2. Among C1 proteins, we revealed the top-enrichment of the Tal1/Scl. Notably, Jurkat and MOLT-3 both harbor a somatic mutation in *TAL1* promoter causing its hyperactivation^27^. Taken together, this set of analyses showed that intrinsic N1 addiction can be predicted by the basal overexpression of proteins functionally connected to N1 at the proteome level.

We next performed gene ontology (GO) enrichment analysis on each cluster (Fig. 2c), which showed that C2 proteins were associated with the biological process “transcription, DNA-templated”, containing a zinc finger domain. The biological process most enriched in C1 was “small molecule metabolic process”. Given the high heterogeneity within C1 proteins, we performed functional network analysis^28^ followed by Markov clustering (MCL)^29^ and GO/pathway enrichment on each sub-cluster^30^ (Fig. 2d). The major GO terms enriched were diverse metabolic processes, involving biosynthesis and catabolism of amino acids, fatty acid degradation, and reactive oxygen species (ROS) metabolic processes. To unravel if there was a common mediator among the diverse GO terms enriched in resistant cells, we queried the Connectivity Map (CMap), a large collection of gene-expression signatures in response to 19,811 compounds and 5075 genetic perturbations^31^. This analysis revealed that the GSI sensitivity profile had the highest similarity to the expression signature of the protein synthesis inhibitors emetine, followed by the mammalian target of rapamycin (mTOR) inhibitor QL-X-138 (Fig. 2e).

To pinpoint phosphosites discriminating GSI sensitivity, we performed a SAM test between the resistant and the sensitive cell line group on the phosphoproteome data, resulting in 107 significantly regulated phosphosites (Supplementary Data 2). Through a functional network analysis on all regulated phosphoproteins, we identified in the second biggest cluster a significant enrichment of the KEGG pathway “mTOR signaling” and the Reactome pathway “Translation initiation complex formation” (Fig. 2f), further proving the CMap prediction. To extend these findings to a larger cell line panel and validate the biological relevance of the findings, we performed an independent analysis on a panel of six GSI resistant and three sensitive T-ALL cell lines (Supplementary Data 1) from the high-throughput Genomics of Drug Sensitivity in Cancer Project (GDSC)^32^. This showed that the resistant cell lines are significantly more sensitive to two different mTOR inhibitors, rapamycin (Fig. 2g) and JW-7-52-1 (Supplementary Fig. 4b). Pten is a known negative regulator of the mTORC1 complex, explaining the observed increase in phosphorylation of mTORC1 targets for the *PTEN* null cell lines, JURKAT and MOLT-3. For the PEER cell line, which does not harbor a *PTEN* null mutation, we discovered through the Cancer Cell Line Encyclopedia database^33^ that it has a nonsense mutation (W164*)^34^ in the *TSC1* locus, which encodes for the negative mTORC1 regulator hamartin. Overall, this set of analyses indicated that intrinsic GSI resistance could potentially be reverted by the inhibition of mTORC1 signaling or protein synthesis, in line with previous reports^35–37^.

### Acquired GSI resistance in DND-41 partially shares aberrant signaling modules with intrinsic resistance

**Fig. 3:**
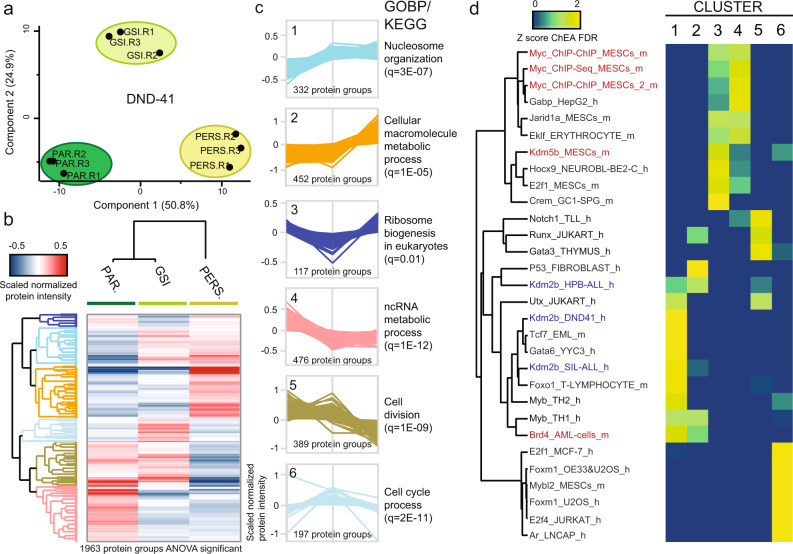
Proteomics analysis of drug-tolerant persister DND-41 cells. **a** Principal component analysis (PCA) of all quantified proteins. **b** Hierarchical clustering of normalized protein intensities scaled per replicate (columns: Pearson correlation; rows: Canberra distance) for significantly regulated proteins among conditions (one-way ANOVA: Benjamini–Hochberg FDR = 0.01; s0 = 0.1; *n* = 3 biologically independent samples). The mean for each protein per condition is shown. **c** Protein expression profiles for each cluster are shown on the left. The most enriched gene ontology term per cluster is shown on the right side of each profile. Enrichment analysis was performed in Perseus using all regulated proteins per cluster (background: proteome). **d** Regulated proteins were ranked based on the log2 fold-change Persister/Parental (Cluster 1–2 and 4–5) or Persister/GSI (Cluster 3 and 6) and ChIP enrichment analysis (ChEA) was performed with the top-100 through Enrichr. The −log10 of the Benjamini–Hochberg corrected *p* values (FDR) are Z-score normalized and the results are shown in a heatmap. PAR parental, PERS persister, GOBP gene ontology biological processes, q Benjamini–Hochberg-corrected *p* value, h human, m murine. Source data are provided as Source Data file.

To gain insights into the biological bases of acquired GSI resistance in DND-41 cells, we performed a PCA analysis on the proteome data (Fig. 3a). Notably, the short-term GSI condition was exactly in between parental and persister cells on component-1. However, it appeared to have unique features, separating from both persister and parental cells on component-2. To explore the reproducibility of our proteome dataset against the microarray data published by Bernstein and coworkers^14^, we performed a linear regression analysis between the two datasets, and we found a considerable direct correlation between the two datasets (Pearson correlation coefficient, *R* = 0.64; Supplementary Fig. 5a). To find the differentially regulated proteins among the three conditions, we performed an ANOVA test. We found 1963 differentially regulated proteins (Supplementary Data 3) and performed hierarchical clustering to determine the directionality of the regulation (Fig. 3b). We identified six expression clusters (C1–6; Fig. 3c). We ranked the proteins in each cluster by fold-change parental/persister and performed ChEA on the top-100 (Fig. 3d). Both in C1 (up in GSI & persister) and C2 (up in persister), we identified the Bromodomain-containing protein 4 (Brd4) significantly enriched. Besides, its expression was slightly higher in persister cells (fold-change = 1.06; FDR-corrected *p* value, *q* = 0.02), confirming what was previously reported^14^. Myb was also enriched in these clusters, showing similarity with intrinsically resistant cells (Fig. 2a). Intriguingly, Kdm2b’s targets were enriched too; but conversely, they were upregulated in intrinsically sensitive cells. C3 (down in GSI) included the Notch target cMyc (*q* = 0.005). Indeed, here ChEA identified proteins mainly being under cMyc transcriptional control. We also identified Kdm5b in this cluster, indicating that its targets are downregulated by GSI and rescued in persister cells. This demethylase was also upregulated in intrinsically resistant cells (fold-change = 1.43; *q* = 0.009). C4 proteins followed the same trend expected for both N1 and its targets (down in GSI & persister). Indeed, N1 itself was part of this cluster (*q* = 0.0002) and ChEA revealed both N1 and Myc enrichment. These findings indicate that the majority of cMyc targets are still under N1 dependency in persister cells, whereas only a defined group of them is re-expressed after the prolonged suppression of N1. In C5 we again identified N1, indicating that its activity is even more downregulated after long-term GSI treatment than short-term. We also found Gata3, whose activity was upregulated in intrinsically resistant cells. C6 (up in GSI) showed a unique profile, and no overlap with other clusters or dataset 1.

To unravel the main signaling pathways significantly affected in persister cells, we performed GO enrichment analysis on each cluster (Fig. 3c; Supplementary Data 4). C1 was enriched in the GO terms: “chromatin binding” and “nucleosome organization”. This suggests that the epigenetic remodeling responsible for resistance development starts from the very beginning of drug exposure. C2 displayed enrichment of several metabolic processes, as “reactive oxygen species metabolic process”. Importantly, ROS clearance enzymes were also overrepresented in the intrinsically GSI resistant cell lines (Fig. 2d). One of the main ROS sources in the cell is byproducts of mitochondrial respiration^38^. Indeed, we found the GO term “mitochondrial matrix” enriched in C2, which included several proteins with electron transfer activity. The Reactome term “respiratory electron transfer” was also enriched in acquired resistant cells (Fig. 2d). C4 proteins appeared to be involved in protein synthesis at multiple levels, which is consistent with the known role of cMyc as master regulator of protein synthesis^39^. C3 also included proteins involved in protein translation, specifically in ribosome biogenesis. This suggests that persister cells might have to partially rescue translation-related processes in order to survive, which agrees with prior findings in intrinsic resistance (Fig. 2e, f). Proteins belonging to C5 and C6 were enriched in cell cycle-related processes. These changes can likely be explained by the slower proliferation rate of persister cells compared to the parental cells (Supplementary Fig. 5b). We then analyzed the regulation of individual proteins involved in the cell cycle, whose expression has been shown to be N1-dependent in T-ALL^40,41^. While Cdk4 (C5; *q* = 0.009), Cdk6 (C4; *q* = 0.0003), and Cdkn1b (C2; *q* = 0.0002) showed expression profiles consistent with N1 inhibition, Cdkn2d (C6; *q* = 0.002), known to be repressed by cMyc^41^, escapes N1 control, consistent with cMyc rescue. We also observed a significant decrease in persister cells of several proteins involved in the DNA damage response (GOBP “DNA repair” enriched in C5). These findings are akin to the recent discovery of “adaptive mutability” in response to Egfr/Braf inhibition in human colorectal cancer^42^.

**Fig. 4:**
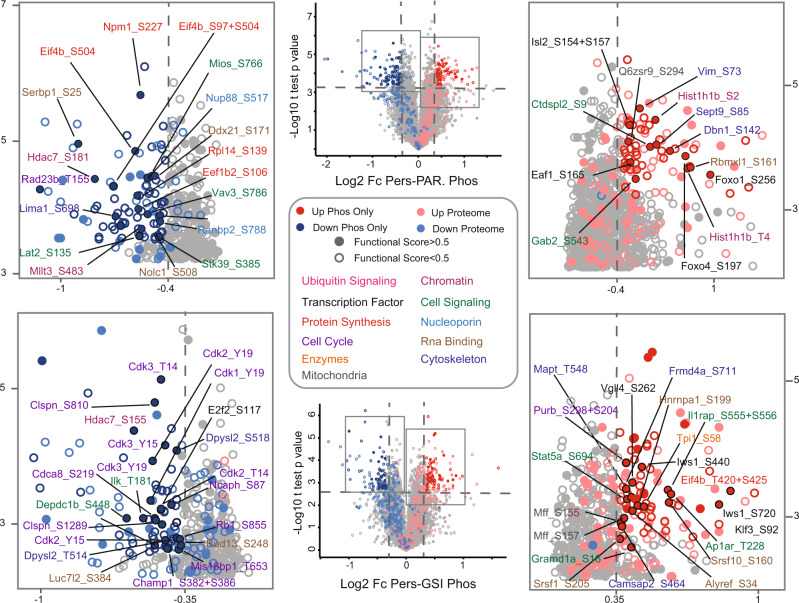
Phosphoproteomics analysis of drug-tolerant persister DND-41 cells. Volcano plot analysis for the Persister/Parental (upper part) and Persister/GSI (lower part) comparisons. Significance was determined through unpaired two-sided *t* test (Benjamini–Hochberg FDR < 0.01 for Persister/Parental and <0.05 for Persister/GSI; *n* = 3 biologically independent experiments). For significance analysis, a threshold on the Log2 fold-change was applied, equal to three times standard deviation, which was calculated on all sites, separately for up and downregulated ones. The annotated plots are magnified sections of the volcano plots in the middle. Phosphosites whose corresponding proteins are regulated on the proteome level, using the same criteria applied to the phosphoproteome, are marked in pink (upregulated) and light blue (downregulated). Phosphosites with a functional score >0.5 are represented as filled circles. Only significantly regulated sites with functional scores >0.5 and not regulated on the proteome level are labeled. In the Pers/GSI comparison, shared regulated sites between comparisons are not labeled. The color name indicates the biological process the corresponding protein is involved.

Next, we analyzed the phosphorylation changes induced by long-term GSI treatment in DND-41 cells compared to parental and short-term GSI-treated cells. In the persister-parental comparison, we identified 491 significantly regulated phosphosites, 231 were upregulated and 260 were downregulated. In the persister-GSI comparison, we identified 565 significantly regulated phosphosites, 287 were upregulated and 278 were downregulated (Supplementary Data 3). Given the relatively high correlation (*R* = 0.45) observed between proteome and phosphoproteome for both comparisons (Supplementary Fig. 5c, d), we performed a volcano plot analysis, where we highlighted only the phosphosites with the highest degree of regulation, which were not regulated on the proteome level to the same extent (Fig. 4). To prioritize functionally relevant phosphosites, we marked only those with a high functional score^43^. This analysis showed a substantial overlap between biological processes regulated on both layers. However, it also allowed us to pinpoint some differences. For example, we noticed the downregulation of the Eukaryotic translation initiation factor 4B (Eif4b) on several sites in the persister-parental comparison, while we observed two different sites upregulated in the persister-GSI comparison, which might represent a way for persister cells to rescue translation initiation, which was highly downregulated on the proteome level.

### Acquired resistance to NOTCH1i in PDX recapitulates the signaling reprogramming observed in vitro

**Fig. 5:**
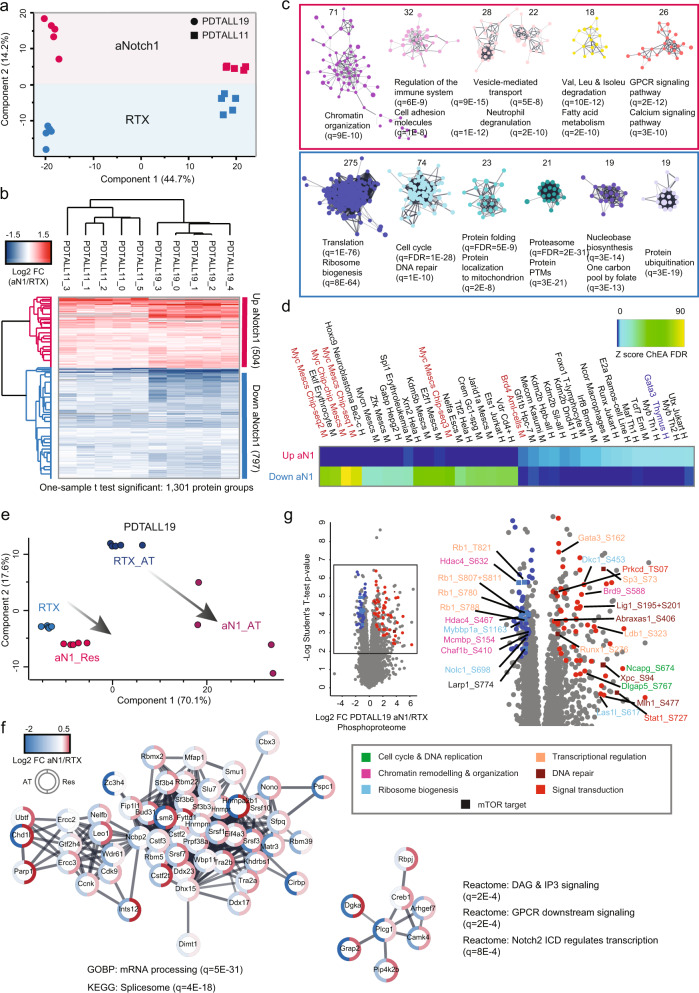
Proteomics and phosphoproteomics analysis of T-ALL PDXs resistant to the anti-Notch1 monoclonal antibody OMP52M51. **a** Principal component analysis of all proteins quantified in at least three mice per group. **b** Unsupervised hierarchical clustering of log2 fold-change anti-Notch1/Rituximab for significantly regulated proteins among conditions (one-sample *t* test: Benjamini–Hochberg FDR < 0.01; s0 = 0.1; *n* = 9 biologically independent mice). Columns: Pearson correlation; rows: Canberra distance. The median for RTX was used to calculate the log2 fold-change. **c** Functional STRING network of significantly regulated proteins represented in b (cutoff: log2 fold-change >2-time standard deviation), generated through the stringApp. Clusters were generated by MCL clustering through the Clustermaker2 app. Gene ontology enrichment analysis was performed through the stringApp. Only the six biggest clusters are shown. Background: proteome. **d** ChIP enrichment analysis (ChEA) was performed on significantly regulated proteins represented in **b** (cutoff: log2 fold-change >2-time standard deviation). The −log10 of the Benjamini–Hochberg corrected *p* values (FDR) are Z-score normalized and the results are shown in a heatmap. **e** Principal component analysis of proteins significantly regulated in the PDTALL19 model, both in the antiNotch1 acute treatment (AT) and resistance (Res) (unpaired two-sided *t* test: permutation-based FDR < 0.05; *n* = 5 or 4, respectively). **f** Functional STRING network of proteins whose expression was upregulated in the resistance and downregulated in the acute treatment (belonging to the pink cluster in Supplementary Fig. 6d). The network was generated through the Omics visualizer app. **g** Volcano plot analysis for the aNotch1/Rituximab comparison in the PDTALL19 phophoproteome. Significance was determined through a SAM test (unpaired two-sided *t* test: permutation-based FDR < 0.01; s0 = 0.1; *n* = 5). Only a selection of phosphosites with functional scores >0.5 is labeled. Color name indicates the biological processes associated with the corresponding protein. RTX control antibody Rituximab, aNotch1 or aN1 anti-Notch1, FC fold-change, h human, m murine, q Benjamini–Hochberg-corrected *p* value. Source data are provided as Source Data file.

In order to gain insights into the biological bases of acquired resistance to aNotch1 mAb in PDTALL11 and PDTALL19 cells, we performed a phospho-proteome profiling of these two PDX models (dataset n. 3, Fig. 1d). Interestingly, PCA on the proteome data showed a separation between control (RTX) and the aNotch1 group on the component-2 in the same direction for both different models (Fig. 5a). This suggests a shared response to the prolonged aNotch1 treatment in the two PDX models. To identify the differentially regulated proteins among control and aNotch1 groups, we performed a one-sample *T*-test on the log2-transformed ratios and identified 1301 regulated proteins, 504 upregulated and 797 downregulated (Fig. 5b; Supplementary Data 5). Next, we performed functional network analysis followed by MCL clustering and GO enrichment on the six biggest sub-clusters (Fig. 5c). Among proteins upregulated by aNotch1 treatment, we found enrichment of the GO terms “Chromatin organization”, “Cell adhesion”, “Valine, leucine, and Isoleucine degradation”, “Fatty acid metabolism”. The same terms were also enriched in intrinsic resistant (Fig. 1d) and persister cells (Supplementary Data 4). The terms “Vesicle-mediated transport”, “Neutrophil degranulation”, “GPCR signaling” and “calcium signaling” appeared unique to this dataset. However, we found the KEGG “Chemokine signaling pathway” enriched in C1 (DND-41), which involves GPCR signaling. Among proteins downregulated by aNotch1 treatment, all clusters showed GO term overlap with persister cells. In particular, the first biggest 275-protein cluster showed enrichment of “translation” and “ribosome biogenesis”; the second biggest cluster included proteins involved in cell cycle and DNA repair. We then performed ChEA on each cluster and identified as enriched most of the TFs previously found in DND-41 dataset (Fig. 5d). The only TF following an opposite trend with respect to DND-41 and acquired resistance was Gata3, whose function was increased in PDX aN1 and decreased in DND-41 persister and intrinsically resistant cells.

Importantly, we evaluated the correlation of our proteomics data with the published microarray dataset in the PDTALL19 model^19^ and found a high positive correlation (*R* = 0.53; Supplementary Fig. 6a). Not only this corroborates the trends observed at the protein level but also indicates that protein regulatory mechanisms observed here are largely driven by gene expression regulation.

As for the PDTALL19 model we also generated a proteome dataset after aNotch1 acute treatment (AT) (Supplementary Data 5), we compared it with the long-term treatment (resistance). First, we analyzed cMyc protein expression and found it showed the same rescue observed in DND-41 persister cells (Supplementary Fig. 6b, c). PCA showed that the directionality of the proteome alteration was common between AT and resistance, with the AT being far more extreme, especially for the downregulated proteins, and with a greater standard deviation of the log2 fold-changes (Fig. 5e, Supplementary Fig. 6d, e). This suggests that resistant cells attenuate NOTCH1i protein signature. A possible explanation can be a partial pathway reactivation, likely due to other members of the Notch family compensating for N1 loss, as previously described^44^. Notably, we identified the transcriptional regulators Rbpj, otherwise known as Csl, and Creb1 among the proteins with a significantly opposite response between AT and aNotch1 (Supplementary Fig. 6f, Fig. 5f). In the absence of active Notch, Rbpj is thought to be primarily a transcriptional repressor^3^. However, it has been proposed that Rbpj might also have a Notch-independent activity^45^. Creb1 has been shown to be significantly enriched on Rbpj DNA-binding motifs^46^. This might represent an adaptation mechanism specific to Notch1-directed therapies, and not GSI, which would require further investigation.

Next, we analyzed the phosphorylation changes induced by aNotch1 compared to RTX. In PTALL19, we identified 294 significantly regulated phosphosites, 173 were upregulated and 121 were downregulated (Supplementary Data 5). We then performed a volcano plot analysis, where we highlighted only the phosphosites with a high functional score (Fig. 5g). We identified a noticeable overlap with the biological processes found for the DND-41 persister dataset (Fig. 4). Some specific examples are the upregulation Stat1 phosphorylation (Stat5a phosphorylation was upregulated in the persister-GSI comparison) and the downregulation of Hdac4 phosphorylation (Hdac7 phosphorylation was downregulated in persister cells for both comparisons).

### Kinase signature associated with resistance to NOTCHi pinpoints PKC activation

**Fig. 6:**
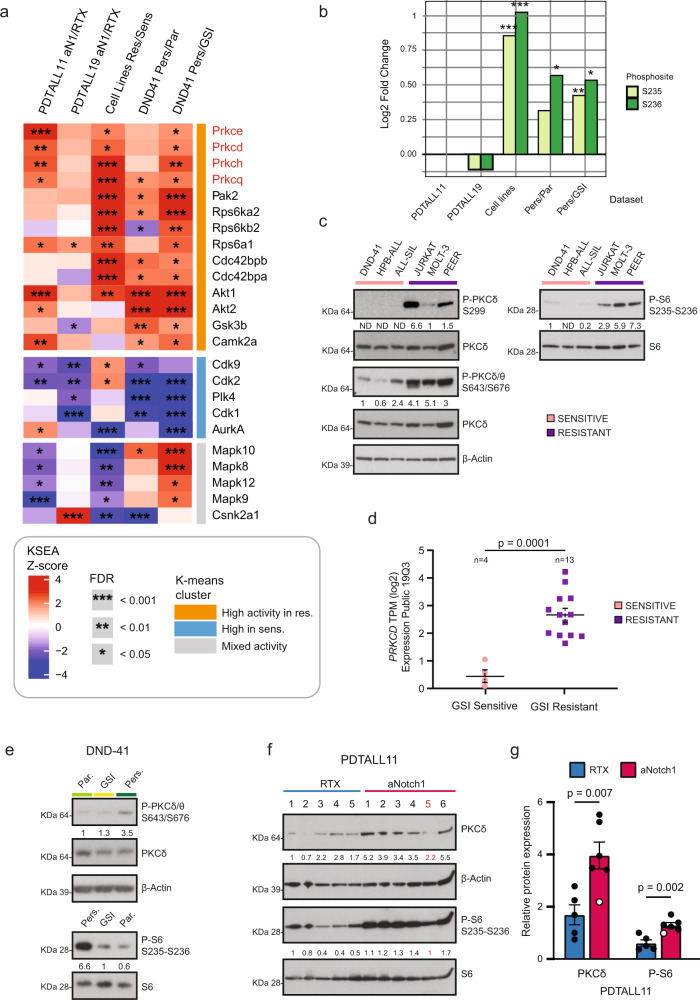
Kinase signature associated with resistance to NOTCHi reveals PKC activation in resistant cells. **a** Kinase-Substrate Enrichment Analysis (KSEA) output is represented in a heatmap. Columns are the different comparisons and rows are the kinases whose activity is significantly regulated (*q*: Benjamini–Hochberg FDR < 0.05) in at least three out of five comparisons. Kinases with less than four substrates were excluded. The three clusters have been generated through k-means row clustering (*n* = 3). KSEA significance derives from *n* = 3 biologically independent experiments per comparison, and it is indicated as *q < 0.05; **q < 0.01; ***q < 0.001. **b** P-S6 S235-S236 Log2 fold-change for the five different comparisons used in **a**. “*/**/***” represent *p* values resulting from unpaired two-sided *t* test. *** = 0.001; ** = 0.01; * = 0.05. **c**, **e**–**f**. Immunoblot analysis of lysates from T-ALL cell lines (C; *n* = 1 for left blot; right blot is representative of *n* = 2 independent experiments), DND-41 cells (E; *n* = 1 for upper blot; lower blot is representative of *n* = 5 independent experiments) and PDTALL11 cells FACS-sorted from the spleen (F; *n* = 1). β-Actin in **c** was run of a different gel. Protein expression was quantified in ImageJ. Mouse n° 5 in **e** (red): outlier in the proteomics data. **d** *PKCδ* gene expression (RNAseq) in a T-ALL cell line panel. The black line indicates the mean and the dashed line is the SEM (*n* = 4–13 biologically independent cell lines). An unpaired two-sided *t* test was used to assess the differences in mean log2 Transcripts Per Million (TPM) between the GSI resistant and sensitive T-ALLs. Data (RNAseq) were taken from the CCLE database (Expression Public 19Q3 dataset). **g** Quantification of the immunoblot in Fig. 6f. The values shown are mean ± SEM (*n* = 5–6 biologically independent mice). An unpaired two-sided *t* test was used to assess the differences in protein expression and phosphorylation. Outliers values are marked in white. RTX Rituximab. Source data are provided as Source Data file.

We next investigated kinase regulation in the GSI resistant and sensitive states through the Kinase-Substrate Enrichment Analysis (KSEA) algorithm, which scores each kinase candidate based on the relative hyper- or dephosphorylation of the majority of its substrates^47^ (Fig. 6a; Supplementary Data 6). This analysis showed that Akt1 was the kinase most significantly upregulated in resistant cells, in line with previous findings^48^. We also found its phosphorylation increased in resistant cells (JURKAT, MOLT-3, and DND-41 persister) by immunoblotting (Supplementary Fig. 7a, b). The common kinase signature associated with GSI resistance also highlighted activation of the protein kinase C (PKC) novel subfamily, including PKC Ɵ, δ, ɛ and η. Moreover, we observed a prominent prevalence of different ribosomal protein S6 kinases (Rps6kb1, Rps6ka1, Rps6ka2). Furthermore, Pak1-2 kinases were found more active in the resistant state.

PKCδ has previously been involved in acquired resistance to targeted therapies in solid tumors^49^. Therefore, we focused specifically at its targets and identified the phosphorylation of serines 235 and 236 on the ribosomal protein S6 (Rps6) significantly upregulated in three out of five comparisons (Fig. 6b). Notably, these phosphorylation events have been previously reported to enhance the initiation of mRNA translation^50^, which is hampered by NOTCHi in resistant cells. Thus, we hypothesized that PKCδ might represent a way for T-ALL cells to escape GSI detrimental effects. Indeed, in intrinsically GSI resistant cells we found an increase of PKCδ protein expression (fold-change = 1.5; *q* = 0.002; Fig. 2d) and enhanced phosphorylation on two of its activating sites: Ser299 (fold-change = 2.3; *q* = 0.004) and Ser645 (fold-change = 2.1; *q* = 0.006). We further validated these findings by immunoblotting (Fig. 6c) and confirmed the upregulation of *PKCδ* gene expression in a broader T-ALL cell line panel^33^ (Fig. 6d). In persister cells and PDTALL11, we did not identify PKCδ either in the proteome or the phosphoproteome. However, we found by immunoblotting the Ser645 upregulated in persister cells (Fig. 6e) and PKCδ protein level upregulated in PDTALL11 after aNotch1 treatment (Fig. 6f, g). Lastly, we identify the activating phosphosite Thr507 (fold-change = 1.8; *q* = 0.002; Fig. 5g, Supplementary Fig. 7d) and the total PKCδ protein level upregulated in PDTALL19 after aNotch1 treatment (Supplementary Fig. 7c). Furthermore, we observed a positive correlation between PKCδ activation and S6 phosphorylation in all models tested (Fig. 6c, e–g; Supplementary Fig. 7d).

We further validated the kinase-substrate relationship between PKCδ and S6 by using two different PKC inhibitors, rottlerin and sotrastaurin, which both induced a massive reduction in S6 phosphorylation in all the resistant cell lines tested (Supplementary Fig. 7e). To exclude that PKCδ regulation of P-S6 was mTOR-mediated, we analyzed the canonical mTOR target P-4e-bp1 Thr37/46 from the same lysates in DND-41 persister cells, and found no regulation (Supplementary Fig. 7f), indicating that S6 is a “bona fide” direct PKCδ substrate.

### Notch and PKC inhibition synergistically affects resistant T-ALL cells’ survival

**Fig. 7:**
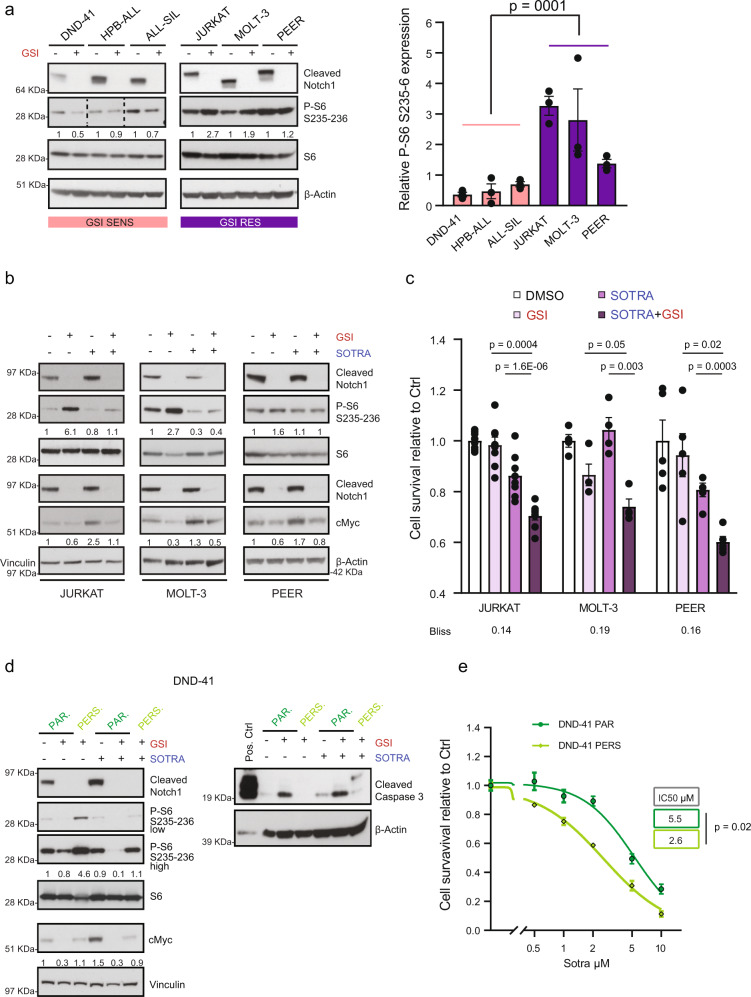
Combination treatment targeting Notch and PKC in cells resistant to NOTCHi. **a** Cells were treated for 5 days with GSI (0.5 μM) and the lysates were immunoblotted for the indicated antibodies. HPB-ALL samples: higher exposure time. The values shown in the right panel are mean ± SEM (*n* = 3 biologically independent samples per cell line). An unpaired two-sided *t* test was used to assess the differences in mean protein expression (*n* = 9 biologically independent samples examined over 3 independent experiments). **b** Cells were treated with GSI (0.5 μM), sotrastaurin (5 μM) or the combination, and the lysates were immunoblotted for the indicated antibodies. P-S6 and S6: 3 days of treatment; cMyc: 5 days of treatment. JURKAT: the blot is representative of 3 (cMyc) or 4 (S6) independent experiments. MOLT-3/S6: *n* = 2. MOLT-3/cMyc and PEER: *n* = 1. **c** Cells were treated for 6 days with GSI (0.5 μM), sotrastaurin (5 μM) or the combination, and they were assayed for cell survival by CCK8 assay. The values shown are mean ± SEM (JURKAT: *n* = 8 biologically independent samples examined over 4 independent experiments; MOLT-3 and PEER: *n* = 4 biologically independent samples examined over two independent experiments). An unpaired two-sided *t* test was used to assess the differences in mean survival. Bliss >0 synergy; <0 antagonism; =0 additive effect. **d** Parental and persister DND-41 cells were treated for 5 days with GSI (0.5 μM), sotrastaurin (5 μM) or the combination, and the lysates were immunoblotted for the indicated antibodies (left blot is representative of *n* = 3 biologically independent experiments; right blot of *n* = 2). **e** Cell survival assay of parental and persister DND-41 cells in response to increasing doses of sotrastaurin (5 μM) for 6 days. The values shown are mean ± SEM (*n* = 12 biologically independent samples examined over three independent experiments). Half-maximal inhibitory concentration (IC50) was calculated using a non-linear regression in GraphPad Prism. An unpaired two-sided *t* test was used to assess the differences of mean IC-50 (log-transformed values). Protein expression was quantified in ImageJ for all blots. SOTRA sotrastaurin, Ctrl DMSO-treated cells. Source data are provided as Source Data file.

N1 positively regulates S6 phosphorylation through cMyc in GSI sensitive T-ALL cell lines^35^. To evaluate N1 effect on S6 phosphorylation in resistant cell lines, we treated all T-ALL cell lines with GSI for 5 days. This highlighted that N1 inhibition was significantly upregulating S6 phosphorylation in resistant cells, while decreasing it in sensitive cells (Fig. 7a).

It is known that mTOR activity increases in the G1/S phase of the cell cycle but decreases in quiescent cells^51^. To rule out that the observed change in the phosphorylation of the mTOR target S6 was caused by GSI-dependent alterations of the cell cycle, we performed a GSI time course in the two sensitive cell lines DND-41 and HBP-ALL. These experiments showed cell cycle arrest after six days of GSI treatment in both cell lines (Supplementary Fig. 1d). Furthermore, we observed a reduction of S6 phosphorylation at the earliest time point of 24 h in both cell lines (Supplementary Fig. 8a), where no change in cell proliferation was detected (Supplementary Fig. 8b), suggesting that the P-S6 decrease is cell cycle-independent. In the ALL-SIL cell line, P-S6 regulation did not show a clear trend within the first 72 h.

We then investigated if the observed GSI-dependent upregulation of P-S6 in resistant cell lines might be a consequence of PKCδ activation. For this purpose, we used the PKC inhibitor sotrastaurin that is a clinical drug (phase II), highly specific towards PKCδ among the different PKC isoforms with very few off-targets at very high concentrations^52^ (Supplementary Fig. 9a, b). We treated resistant cell lines with GSI, sotrastaurin (at a concentration lower than the experimentally determined IC-50; Supplementary Fig. 9c) and in combination (Fig. 7b, Supplementary Fig. 9d). The addition of sotrastaurin to GSI significantly reduced the increase in P-S6 in all the cell lines tested. This crosstalk was specifically directed to P-S6 but not mTOR, as we observed no regulation of P-4e-bp1 (Supplementary Fig. 9h). Moreover, PKCδ inhibition alone resulted in a significant upregulation of cMyc protein expression, which was completely abrogated by the combination treatment. The same combination treatment resulted in a synergistic effect on cell growth, as shown by a Bliss independence analysis (Fig. 7c). These results were independently confirmed in JURKAT cells by using the PKCδ inhibitor rottlerin (Supplementary Fig. 9e–g).

To determine if PKCδ activation was responsible for P-S6 upregulation in DND-41 persister cells (Fig. 6e), we treated parental and persister cells with GSI, sotrastaurin, and the combination (Fig. 7d). The combination of sotrastaurin and GSI completely abrogated P-S6 increase in persister cells and reduced P-S6 in the parental cells even further than GSI alone. Moreover, PKCδ inhibition alone resulted in a significant increase in cMyc protein expression in parental cells, which was abrogated by GSI, displaying the same behavior observed in intrinsically resistant cells. Also, persister cells exhibited a significant and higher than two-fold increase in sotrastaurin IC50 compared to parental cells (Fig. 7e) and the combination of GSI and sotrastaurin increased T-ALL cells apoptosis, as proved by the upregulation of cleaved caspase-3 (Fig. 7d).

Taken together, these experiments show that PKCδ inhibition can restore GSI sensitivity in resistant cells. Moreover, they highlight that the expression of PKCδ and N1 is mutually exclusive, as the inhibition or either of the two leads to the activation of the other. To validate if this is a general feature in T-ALL, we found a significant negative correlation (*R* = −0.6; *p* = 0.0009) between *N1* and *PKCδ* gene expression in a T-ALL cell line panel^33^ (Supplementary Fig. 10a). Furthermore, we analyzed RNAseq data from a cohort of 264 T-ALL patients^5^, and we confirmed the general negative correlation observed in the cell line panel (*R* = −0.14; *q* = 0.055; Supplementary Fig. 10b).

### PKCδ is indispensable for the development of adaptive GSI resistance

**Fig. 8:**
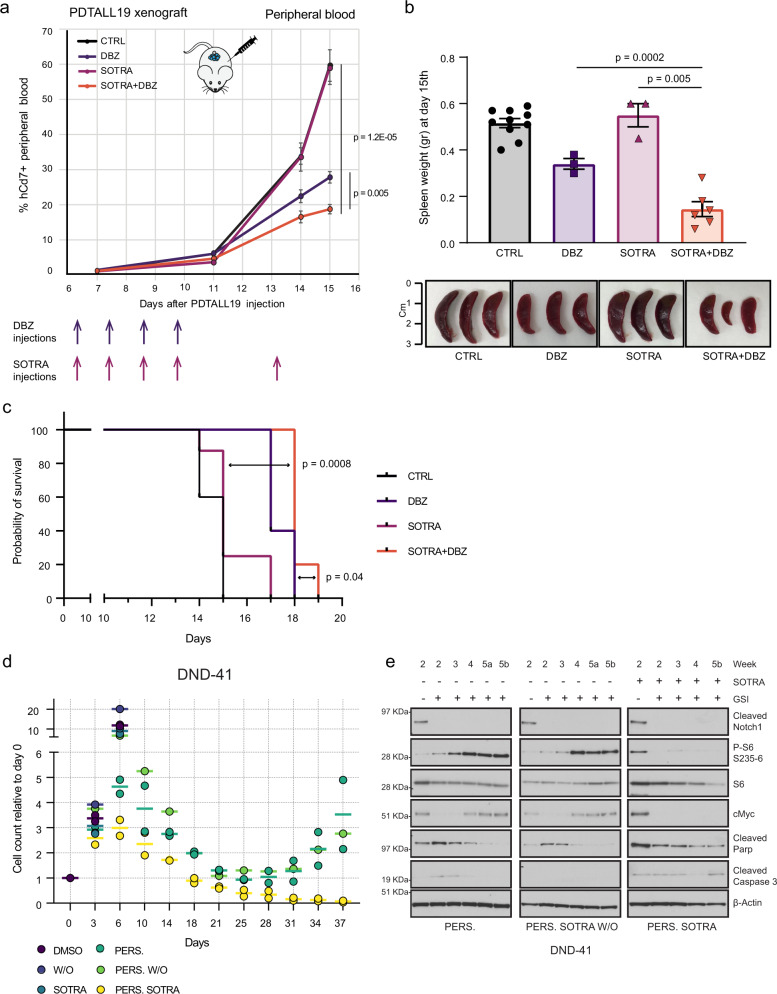
Combination treatment targeting Notch and PKC in cells sensitive to NOTCHi. **a** NOD/SCID mice were injected with 1 × 10^6^ PDTALL19 cells on day 1. On day 7, the mice were randomized and treated with vehicle (CTRL), GSI (DBZ: dibenzazepine), sotrastaurin (SOTRA) or sostrastaurin + DBZ. DBZ was administered in a 4 days on/3 days off regimen (4 doses in total). Sotrastaurin was administered in a regimen of 5 days on/2 days off (5 doses in total). Flow cytometry analysis of human Cd7^+^ cells in peripheral blood was performed on day 7, 11, 14, and 15 (sacrifice day). The values showed are mean ± SEM and significance were assessed by unpaired two-sided *t* test and. Vehicle *n* = 6 biologically independent mice; DBZ *n* = 3; sotrastaurin *n* = 5; sotrastaurin + DBZ *n* = 6. Gating strategies are provided in Supplementary Fig. 12. **b** Spleen weight (top) and size (bottom) after mice sacrifice after 15 days from PDTALL19 injection. The values showed are mean ± SEM and significance were assessed by unpaired two-sided *t* test. Vehicle *n* = 10 biologically independent mice; DBZ *n* = 3; sotrastaurin *n* = 3; sotrastaurin + DBZ *n* = 6. **c** Kaplan–Meier survival curves for PDTALL19. Significance was assessed by a two-sided log-rank (Mantel-Cox) test. Vehicle *n* = 10 biologically independent mice; DBZ *n* = 5; sotrastaurin *n* = 8; sotrastaurin + DBZ *n* = 5. **d** DND-41 were pretreated with sotrastaurin (5 μM) for 3 days. Next, GSI treatment (0.5 μM) was performed up to 5 weeks for three different populations: persister cells (DMSO + GSI), sotrastaurin washout (DMSO + GSI), and sotrastaurin (SOTRA + GSI). Live cell count was performed by trypan blue exclusion. *n* = 2 independent experiments for persister and persister/sotrastaurin; *n* = 1 for sotrastaurin washout. **e** Cells were harvested at different time points (2–5 weeks) and immunoblotted with the indicated antibodies. 5a and 5b indicate two-time points within week 5. The experiment was performed once. W/O washout. Source data are provided as Source Data file.

Since we observed an early appearance of the GSI resistance protein signature in both DND-41 and PDTALL19 datasets (Figs. 3a, 5e), we hypothesized that the combination of GSI and sotrastaurin could have synergistic effects on GSI sensitive cells, too. To test this, we therefore examined its efficacy on PDTALL19 in vivo. We engrafted NOD/SCID mice with PDTALL19 cells and initiated the treatment when a leukemic burden of ~1–2% of human CD7^+^ in peripheral blood was reached (day 7). Randomized groups were treated with vehicle, the GSI DBZ, sotrastaurin or the DBZ-sotrastaurin combination. The development of leukemia was evaluated by regular flow cytometric analysis of human CD5 and CD7 T-ALL markers on peripheral blood. The vehicle-treated mice reached the human endpoint (natural death or a leukemic burden higher than 40% CD7-positive (CD7^+^) in peripheral blood) by day 15. On the same day, three mice per group were sacrificed and leukemic burden was evaluated in peripheral blood (PB), spleen and bone marrow (BM). We observed a significant difference in the percentage of both CD5^+^ and CD7^+^ cells in PB between the combination and single-agent therapies (Fig. 8a, Supplementary Fig. 11a). In the BM we observed a significant difference only for the CD7^+^ cells (Supplementary Fig. 11b). The spleen was almost completely infiltrated in all mice (Supplementary Fig. 11c), but we observed a significant reduction of the spleen weight in the combination compared to single-agent therapies, which was directly reflected in the spleen size (Fig. 8b). Following sacrifice, we further observed that combination therapy significantly prolonged survival compared to treatment with single-agent therapy (Fig. 8c).

**Fig. 9:**
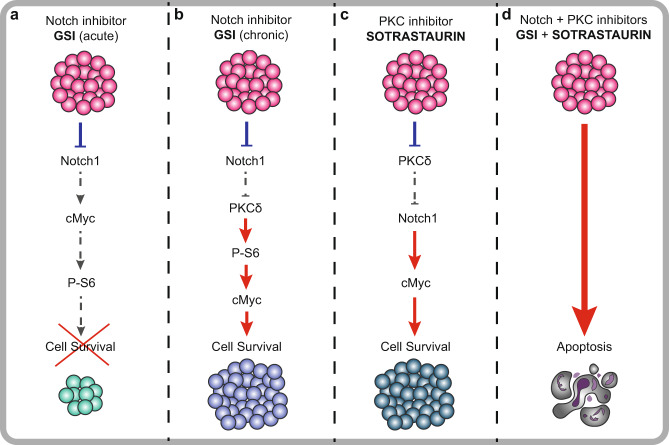
Graphical model for therapeutic inhibition of Notch and PKC in T-ALL cells to prevent drug resistance. **a** In sensitive cells, NOTCHi leads to downregulation of its transcriptional target cMyc, which in turn regulates S6 phosphorylation in a mTORC1-dependent manner. **b** After chronic administration of Notch inhibitors, cells adapt to Notch absence through the upregulation of PKCδ, which phosphorylates S6 and sustains cMyc expression in a Notch-independent manner. **c** Notch and PKCδ activation show a mutually exclusive pattern. In fact, PKC inhibition by sotrastaurin leads to upregulation of cMyc expression. **d** The combined inhibition of Notch and PKC has a synergistic effect and fully inhibits the development of acquired resistance.

Next, we sought to determine the effects of the GSI-sotrastaurin combination on resistance development. To do so, we treated the DND-41 parental cells with GSI, sotrastaurin, and the combination. We then compared resistance development induced by long-term GSI treatment between untreated and sotrastaurin-treated persister cells. This experiment showed that continuous PKCδ inhibition completely abolished the rescue of cell growth of drug-tolerant persister cells (Fig. 8d) and induced a stable apoptotic response (Fig. 8e). P-S6 was upregulated already after 2 weeks of GSI treatment. Conversely, cMyc expression was still downregulated after 2–3 weeks and only rescued between week 3rd and 4th. When GSI was administered together with sotrastaurin, no rescue of either P-S6 or cMyc was observed. To discern if the acquired resistant cells were a preexisting population, we pre-treated the DND-41 parental cell with sotrastaurin and then washed the drug out, before GSI treatment. This experiment displayed that sotrastaurin pretreatment did not affect persister cells survival (Fig. 8d), neither P-S6 nor cMyc expression (Fig. 8e). Taken together, this experiment showed that P-S6 is an early hallmark of GSI resistance development that occurs through an adaptive response led by PKCδ (Fig. 9).

## Discussion

Acquired resistance to cancer therapy can emerge in response to drug treatment either from the expansion of pre-existing resistant cell clones harboring a resistance-causing mutation, or “de novo” through an intermediate drug-tolerant state^53^, that functions as a reservoir for acquiring of diverse resistance-causing mutations^54^. Our results provide a proof of principle that cells intrinsically resistant to N1 inhibition by genetic mutations and drug-tolerant acquired resistant cells share several signaling modules, despite the different origin of resistance.

Molecular mechanisms of resistance to targeted therapy can fall into one of three main categories: pathway reactivation, bypass, and indifference^10^. Here we show that persister cells completely switch off Notch signaling, but bypass this inhibition through the activation of several parallel pathways, like PKC activation, converging on cMyc and protein translation. Conversely, PDTALL19 cells seem to become resistant both through pathway reactivation and bypass. Furthermore, we highlight several alternative ‘cellular states’ (pathway indifference), shared between intrinsic and acquired resistance, which could represent further potential targetable vulnerabilities. We observe a metabolic switch towards valine, leucine, and isoleucine degradation and fatty acid metabolism in resistant cells. Remarkably, metabolic rewiring converging on alterations of lipid metabolism has been reported also by Agnusdei et al. in the PDTALL19 model resistant to antiNotch1 mAb^19^. We also identify the upregulation of several classes of ROS scavenging enzymes. Notably, increased detoxification from ROS has previously been associated with cancer drug resistance^55,56^. We also notice that acquired resistant cells cannot divide as fast as the parental cells, indicating they could reside in a semi-quiescent cell state allowing them to survive GSI detrimental effects. A similar mechanism was shown in neuroblastoma in response to doxorubicin treatment^57^. This alternative state can also explain how the cells survive to high ROS levels, since a faster DNA replication would lead to high replication stress, genome instability, and cell death, especially in presence of lower DNA repair activity, which was observed in acquired resistant cells.

Acquired drug resistance can appear when drug-resistant tumor cell subpopulations expand under the selection pressure of the drug to drive recurrence (Darwinian model). Cancer cells can also become resistant through rewiring of signaling networks (adaptation model)^58^. Here we notice an early appearance of the GSI resistance protein signature in both DND-41 and PDTALL19 datasets. This indicates that rewiring of signaling networks happens rapidly after N1 inhibition, pointing towards the hypothesis of an adaptive mechanism for acquired GSI resistance. Indeed, pretreatment of the DND-41 population with sotrastaurin, followed by long-term GSI treatment, still leads to drug resistance development, whereas resistance cannot occur under continuous combined GSI and sotrastaurin treatment.

Finally, our work suggests that combination treatment of GSI and sotrastaurin evades acquired resistance development. These findings could have translational potential in the near future, especially if the presenilin-1-specific GSI, which showed very limited toxicity effects in vivo^59^, will be tested in clinical trials. However, more work will be needed to outline the role of the different members of the PKC family in the observed synergism with GSI.

Overall, the quantitative datasets described here represent a resource of Notch1 signaling in T-ALL. Through our proteomics approach, we were able to confirm previous findings and uncover unknown mechanisms of signaling rewiring responsible for GSI resistance in T-ALL, paving the way for unique opportunities of therapeutic intervention.

## Methods

### Cell lines

The human cell lines JURKAT (Clone E6-1) and MOLT-3 were purchased from ATCC. The human cell lines DND-41, HPB-ALL, ALL-SIL, and PEER were purchased from DSMZ. JURKAT, DND-41, and ALL-SIL were cultured in 90% RPMI-1640 Glutamax (Gibco), supplemented with 10% of heat-inactivated fetal bovine serum (FBS, Gibco) and 10,000 U/mL penicillin and streptomycin (Invitrogen). HPB-ALL and PEER were cultured in 80% RPMI-1640 Glutamax, supplemented with 20% heat-inactivated FBS and 10,000 U/mL penicillin and streptomycin. All cell lines were cultured at 37 °C, in a humidified incubator with 5% CO_2_, and routinely checked for mycoplasma, using the EZ PCR Mycoplasma detection Kit (Biological Industries); only mycoplasma-free cells were used. To avoid artefacts derived from long-term culture of immortalized cell lines, typically cells were not kept in culture for more than 2 months, starting from frozen stocks derived from the commercial repository.

Persister cells were generated by treating the DND-41 cells for up to 12 weeks with 0.1–1 μM GSI (GSI XXI, Compound E; Calbiochem). The GSI was chosen based on the original publication where DND-41 persister cells were generated for the first time^14^.

Cell count was performed by trypan blue exclusion on the automated CytoSMART cell counter (Corning).

### Drug treatment

Prior to drug treatment, cells were resuspended in fresh medium at 0.1–1 million cells per ml. The concentration of GSI used was 0.5 μM. Minimum rottlerin (Calbiochem) concentration for short-term treatment prior to immunoblot experiments was chosen at 3 μM; for long-term drug treatment, a concentration of 0.75 μM has been used. Sotrastaurin (Selleckchem) doses used for short-term treatment prior to immunoblot experiments were chosen at 1, 5, and 10 μM; a 5 μM dose was used for long-term drug treatments.

### In vivo drug treatment of T-ALL PDXs

Primary T-ALL cells (PDTALL) were obtained from the bone marrow of newly diagnosed ALL pediatric patients, according to the guidelines of the local ethics committee. For initial xenografts establishment, 6–9 weeks-old NOD/SCID mice (NOD.CB17-Prkdcscid/NCrCrl), purchased from Charles River Laboratories, were injected intravenously (i.v.) with 1 × 10^7^ T-ALL cells in 300 ml of Dulbecco’s phosphate-buffered saline. Secondary transplants were obtained by injecting 5 × 10^6^ T-ALL cells per mouse^18^.

Resistance to NOTCHi was developed as previously described^19^. 5 × 10^6^ cells of PDTALL11 and PDTALL19 cells were i.v. injected in NOD/SCID mice (5–6 mice/group). Animals were intraperitoneally (i.p.) treated with the humanized anti-human NOTCH1 mAb OMP52M51 (Oncomed Pharmaceuticals Inc.) or control antibody (Rituximab, Roche) until disease progression. Both antibodies were administrated weekly at 20 mg/kg, starting two days after i.v. injection of T-ALL cells. In the acute treatment experiment (only PDTALL19), mice were treated with Rituximab or OMP52M51 four days prior to sacrifice.

To perform in vivo DBZ/sotrastaurin treatment, PDTALL19 cells were i.v. injected in NOD/SCID mice (1 × 10^6^ cells/mouse; 8–10 mice/group) and animals were i.p. treated with vehicle (2.5% DMSO + 0.5% methylcellulose/0.1% Tween-80 for DBZ and 4% DMSO + 30% PEG 300/H20 for sotrastaurin), DBZ (Tocris), sotrastaurin or the combination. DBZ was administered at 5 mg/kg on a 4 days on/3 days off regimen, as previously described^60^. Sotrastaurin was administered at 40 mg/kg on a 5 days on/2 days off. Treatment started when a leukemic burden of ~1–2% of human CD7-positive (CD7^+^) in peripheral blood was reached (7 days after i.v. injection).

Mice were sacrificed where they reached 40% of human CD7^+^ in peripheral blood. Lymphocytes were isolated from peripheral blood, bone marrow, and spleen for further analysis (flow cytometry, proteomics or immunoblotting).

Procedures involving animals conformed to current laws and policies (EEC Council Directive 86/609, OJ L 358, 12/12 1987) and were authorized by the Italian Ministry of Health (894/2016-PR).

### Whole-cell lysis and immunoblotting

For biochemical assays, whole-cell lysates were generated by using modified radioimmunoprecipitation assay (RIPA) buffer [1% NP-40, 0.1% Natrium Deoxycholate, 150 mM NaCl, 1 mM EDTA, 50 mM Tris-HCl (pH 7.5)]. Lysis buffer was supplemented with 5 mM b-glycerophosphate, 5 mM sodium fluoride, 2 mM sodium orthovanadate, and the mini EDTA-free protease inhibitor cocktail (Roche). Protein concentration was measured by using Bradford (Bio-Rad). For immunoblot analysis, 10–50 μg of protein was separated by SDS-PAGE (NuPAGE 4–12% Bis-Tris Gel, Invitrogen), transferred to a nitrocellulose membrane and incubated with the primary antibodies. A list of all the antibodies used in this publication is provided in Supplementary Table 1 in the supplementary information. After incubation with a secondary antibody (Jackson ImmunoResearch), the membranes were incubated with the Novex ECL Chemiluminescent Substrate Reagent Kit (Invitrogen). Quantification analysis was performed through ImageJ software and images were processed by using GIMP and Adobe Illustrator software. The values shown for quantification are mean ± SEM.

### Cell survival assay

Cells were seeded at 0.1–0.5 × 10^6^/mL within 24–48 well plates. Medium and drugs were replenished every 3 days. All conditions contained the same amount of DMSO. Cell viability was measured using the cell counting kit-8 (tebu-bio) according to the manufacturer’s instructions. Absorbance was measured using a microplate reader (BGM LABTECH). Each replicate was measured twice (two technical replicates). All experiments were performed in at least two biological replicates. The IC50 values were derived from a four-parameter non-linear curve fitting using GraphPad Prism software.

### Flow cytometry analysis of T-ALL PDX cells

Leukemic cells were collected from peripheral blood, spleen, and BM of control and treated mice. Fluorescein isothiocyanate-labeled mAb against CD5 and phycoerythrin-Cy5-labeled mAb against CD7 (Coulter) were used for the detection of T-ALL cells in mouse samples. Samples were analyzed on the BD FACSCelesta flow cytometer. BD FACSDiva software was used for data acquisition and analysis. Gating strategies are provided in Supplementary Fig. 12.

### Cell cycle analysis

1–2 × 10^6^ cells were stained with the ghost dye violet 450 (Tonbo Biosciences), then fixed and permeabilized by using the Foxp3/Transcription Factor Staining Buffer Set (eBioscience), according to the manufacturer’s instructions. DNA was stained by using a solution of 7-Aminoactinomycin D (7-AAD; Sigma-Aldrich), Ribonuclease A from bovine pancreas (Sigma-Aldrich) and PBS (Gibco). Cells were analyzed on a LSR Fortessa (BD Biosciences) flow cytometer; raw data were acquired through the BD FACSDiva software and analyzed by using the ModFit LT software. Only single and live cells were used for the analysis. Gating strategies are provided in Supplementary Fig. 12.

### Cell lysis and digestion

#### GndCl-based lysis and in solution digestion

Cell pellet was resuspended in boiling lysis buffer (6 M guanidine hydrochloride, GndCl, Sigma-Aldrich), supplemented with 5 mM tris(2-carboxyethyl)phosphine (TCEP), 10 mM chloroacetamide (CAA), 100 mM Tris-HCl (pH 8.5). Cells were boiled for an additional 10 min and further lysed by micro tip sonication (Vibra-Cell VCX130, Sonics, Newtown, CT) for 2 min with pulses of 1 second on and 1 s off at 80% amplitude. Protein concentration was estimated by Bradford assay (Bio-Rad).

The lysates were digested with Lys-C (FUJIFILM Wako Pure Chemical Corporation) in an enzyme/protein ratio of 1:200 (w/w) for 1 h followed by a 6-fold dilution with 25 mM Tris, pH 8.5, to 1 M GndCl and further digested overnight with trypsin (Sigma-Aldrich) 1:100 (w/w) at 37 °C.

#### SDS-based lysis and on-bead digestion

Cellular pellet was resuspended in boiling lysis buffer (5% sodium dodecyl sulfate (SDS), 5 mM tris(2-carboxy- ethyl)phosphine (TCEP), 10 mM chloroacetamide (CAA), 100 mM Tris, pH 8.5). Cells were boiled for an additional 10 min and further lysed by micro tip probe sonication. Protein concentration was estimated by BCA assay (Pierce).

Protein digestion using the PAC method^61^ was automated on a KingFisher™ Flex robot (Thermo Fisher Scientific) in 96-well format, as previously described^21^. The 96-well comb is stored in plate #1, the sample in plate #2 in a final concentration of 70% acetonitrile and with magnetic amine beads (ReSyn Biosciences) in a protein/bead ratio of 1:2. Washing solutions are in plates #3–5 (95% Acetonitrile (ACN)) and plates #6–7 (70% Ethanol). Plate #8 contains 300 μl digestion solution of 50 mM ammonium bicarbonate (ABC), LysC in an enzyme/protein ratio of 1:500 (w/w) and trypsin in an enzyme:protein ratio of 1:250. The protein aggregation was carried out in two steps of 1 min mixing at medium mixing speed, followed by a 10 min pause each. The sequential washes were performed in 2.5 min and slow speed, without releasing the beads from the magnet. The digestion was set to 12 h at 37 °C with slow speed.

### Peptide cleanup

Protease activity was quenched by acidification with trifluoroacetic acid (TFA) to a final concentration of ~1%, and the resulting peptide mixture was concentrated using reversed-phase Sep-Pak C18 Cartridge (Waters). Peptides were eluted off the Sep-Pak with 300 μl of 40% acetonitrile (ACN) followed by μl of 60% ACN. The ACN was removed by vacuum centrifugation for 40 min at 60 C and the final peptide concentration was estimated by measuring absorbance at 280 nm on a NanoDrop 2000C (Thermo Scientific).

For single-shot DIA proteome analysis, samples have been directly loaded on C18 Evotips (Evosep) after acidification.

### TMT labeling

Peptide (250 µg) from each sample was labeled with 1 of 11 different TMT reagents (1 μl of TMT per 10 μg per channel), according to the manufacturer’s protocol (Thermo Fisher Scientific). After labeling, samples were pooled, acidified, and either used for phosphopetide enrichment or concentrated on SepPaks (Waters). After SepPaks, peptides were eluted with 300 μl of 40% ACN, followed by 300 μl of 60% ACN and by 300 μl of 80% ACN. The combined eluate was reduced by SpeedVac.

### TiO2 phosphopeptide enrichment

After TMT labeling, samples were pooled, acidified, and adjusted to 80% ACN/6% TFA, and phosphopeptides were enriched by two sequential rounds of titansphere chromatography, using titanium dioxide beads (TiO2; GL Sciences), as previously described^62^. TiO2 beads were pre-incubated in 2,5-dihydroxybenzoic acid (DHB; 20 mg/ml; Sigma-Aldrich) in 80% ACN/1% TFA (5 µl/mg of beads) for 20 min. Beads equivalent to 2X starting protein amount were added to each sample, which was then incubated for 20 min while rotating. Beads were transferred to C8 (Sigma-Aldrich) StageTips and washed with 10% ACN/6% TFA, 40% ACN/6% TFA, and 60%/6% TFA. Phosphopeptides are then eluted with 5% ammonia and 10% ammonia/25% ACN and subsequently loaded onto C18 (Sigma-Aldrich) StageTips. Peptides were eluted with 40 and 60% ACN and subjected to Offline High pH reversed-phase HPLC fractionation.

### Automated Ti-IMAC phosphopeptide enrichment

Phosphopeptide enrichment was carried out on a KingFisher™ Flex robot (Thermo Fisher Scientific) in 96-well format, as previously described^21^. 50–250 µg of peptide were used for enrichments with 5–25 µl of magnetic Ti-IMAC HP beads (ReSyn Biosciences). Briefly, the 96-well comb is stored in plate #1, Ti-IMAC HP beads in 100% ACN in plate #2 and loading buffer (1 M glycolic acid, 80% ACN, 5% TFA) in plate #3. The sample volume is minimum doubled with loading buffer while kept in a total of 300 µl and added in plate #4. Plates 5–7 are filled with 500 µl of washing solutions; loading buffer, 80% ACN, 5% TFA, and 10% ACN, 0.2% TFA respectively. Plate #8 contains 200 µl of 1% ammonia for elution. The beads were washed in loading buffer for 5 min at medium mixing speed, followed by binding of the phosphopeptides for 20 min and medium speed. The sequential washes were performed in 2 min and fast speed. Phosphopeptides were eluted in 10 min at medium mixing speed. The eluate is acidified with TFA and loaded directly on EvoTips for further MS analysis.

### Offline high pH reversed-phase HPLC fractionation

For proteome analysis of TMT-labeled samples, 120 µg of TMT-labeled peptides were resuspended in 5 mM ABC and fractionated using a reversed-phase Acquity CSH C18 1.7  μm 1 × 150 mm column (Waters) on an UltiMate 3000 high-pressure liquid chromatography (HPLC) system (Thermo Fisher Scientific) by using the Chromeleon software (Thermo Fisher Scientific). The instrument operated at 30 μl per minute. Buffer A (5 mM ABC) and buffer B (100% ACN) were used. 46 fractions were collected without concatenation, as previously described^63^. For phosphoproteome analysis, TMT-labeled samples, enriched for phosphopeptide as mentioned above, were subject to fractionation using the same system as for total proteome but collecting 12 concatenated fractions. Peptides were separated by a multi-step gradient as follows: 0–10 min 6.5%B–15%B, 10–59.5 min 15%B–30%B, 59.5–67 min 30%B–65%B, 67–70 min 65%B–80%B, 70–77 min 80%B, 78–87 min 6.5%B.

### Nanoflow LC–MS/MS

All TMT samples were analyzed on an Easy-nLC 1000 (Proxeon) coupled to a Q-Exactive HF-X MS (Thermo Fisher Scientific)^20^, equipped with a nanoelectrospray source. Peptides were separated on a 15-cm analytical column (75-µm inner diameter) in-house packed with 1.9-µm C18 beads (Dr. Maisch). The column temperature was maintained at 40 °C, using an integrated column oven (PRSO-V1, Sonation). For TMT-proteome, peptides eluted during a 30 min gradient, ranging from 10% buffer B (80% ACN and 0.1% formic acid) to 30% B in 25 min and ramped to 45% B in 5 min at a flow rate of 350 nL/min. The washout followed at 80% buffer B for 4 min. A similar gradient but with 60 min duration was employed for phosphoproteomics samples. The MS was operated in data-dependent acquisition (DDA) mode. Raw data were acquired through the Xcalibur software (Thermo Fisher Scientific).

Single-shot label-free samples were analyzed on the Evosep One system^21^ using an in-house packed 15 cm, 150 μm i.d. capillary column with 1.9 μm Reprosil-Pur C18 beads (Dr. Maisch) using the pre-programmed gradients: 30 samples per day for proteome; 60 samples per day for phosphoproteome. The column temperature was maintained at 60 °C and interfaced online with the Orbitrap Exploris 480 MS (Thermo Fisher Scientific)^21^, equipped with a nanoelectrospray source. Raw data were acquired through the Chronos software (LEAP). Proteomes were analyzed in technical duplicates.

Specific MS/MS settings are provided in the supplementary methods.

### Raw MS data processing

DDA raw LC-MS/MS files were processed using the MaxQuant software suite^64^, with Andromeda search engine^65^ against the human Swissprot database (downloaded between April 2017 and January 2019). A false discovery rate (FDR) of 1% was used at the peptide and protein level. TMT correction factors were not used. Carbamidomethylation of cysteine was specified as fixed modification. Protein N-terminal acetylation, oxidation of methionine (M), Deamidation (NQ), cyclization of N-terminal glutamine to pyroglutamate (PyroGlu) and phosphorylation of serine, threonine, and tyrosine residues (pSTY) were considered as variable modifications and used for protein quantification. Protein quantitation required a minimum one peptide (unique or razor). For Phosphoproteomics data, maximum four variable modifications were allowed per peptide. The “maximum peptide mass” was set to 7500 Da and the “modified peptide minimum score” and “unmodified peptide minimum score” were set to 25, as previously described^20,21^. More information is in the supplementary methods.

All DIA raw files were processed with Spectronaut^66^ (Biognosys), using a library-free approach (directDIA), as previously described^67^. The FASTA file used was the human Swissport database, downloaded in January 2019 and containing 21,074 entries. We also included the MaxQuant contaminants list, which included 245 entries. The PTM localization filter was disabled for proteome analysis and Deamidation (NQ) was included as variable modification. Besides that, default settings were used. For phospho-enriched samples, pSTY was also added as a variable modification and PTM localization cut-off was set to 0.75.

### Bioinformatics analysis of MS data

Most of the bioinformatics analysis has been performed using the Perseus software^68^. Detailed information on data normalization for each dataset is provided in the Supplementary methods.

Contaminants were excluded from all statistical analyses.

Unsupervised hierarchical clustering analysis (Canberra distance for row and Pearson correlation for column clustering), PCA, and volcano plot analysis were performed in Perseus.

Transcription factor enrichment analysis was performed using the ChEA 2016 database^25^, through the online web tool Enrichr^26^.

Network analysis was performed in Cytoscape by using the stringApp^30^ to generate the protein association networks. Clustering network analysis has been performed by using the MCL algorithm through the Cytoscape plugin clusterMaker2^29^.

GO enrichment and pathway analyses were performed in Perseus by performing a Fisher-exact test, with Benjamini–Hochberg correction of the *p* values, or by using the stringApp^30^ in Cytoscape. All (phospho)-proteins quantified were used as background.

Connectivity map analysis^31^ was performed and visualized by using the online tool available at https://clue.io/. To identify a shortlist of drugs potentially able to revert the GSI resistant cell proteome towards the sensitivity state, we sorted the proteins more abundantly expressed in resistant cells by their t-test statistics value (resistant/sensitive) and queried the first top 150 as downregulated in the CMap. Conversely, proteins more abundant in sensitive cells were queried as upregulated in the CMap, following the same sorting criteria.

The Kinase-Substrate (K-S) Enrichment Analysis (KSEA)^47^ was performed by using the R package KSEA app^69^. To extract known K-S relationships the database phosphositeplus (PSP) was used. Since only a small portion of experimentally identified phosphosites have documented K-S annotations in PSP, we expanded the K-S database to include relationships predicted by NetworKIN with a NetworKIN score minimum of 3. The chosen FDR cutoff (Benjamini–Hochberg correction) for significantly differential kinase activity was set at 0.05. Kinases with <4 substrates were excluded from the analysis. KSEA was run for five different comparisons: aN1/RTX PDTALL19, aN1/RTX PDTALL11, PERS/PAR, PERS/GSI and RES/SENS. K-means row clustering (*N* = 3) of the KSEA Z-scores was performed using the R package Complex Heatmap.

Linear regression analysis was performed in Excel (Supplementary Figs. 5 and 9) or in R.

### Drug combination effect

Bliss independence analysis was performed as previously described^70^. Two drugs A and B have additive effects if the surviving fraction (S) of cells upon simultaneous administration, denoted S(AB), is equal to the product of S(A) and S(B), when the drugs are given separately. Therefore, there is synergy if S(AB) < S(A)S(B), and antagonism if S(AB) > S(A)S(B). Here, Bliss index was calculated as S(AB)-S(A)S(B): a positive value denotes synergy; a negative value denotes antagonism; a value of 0 denotes an additive effect.

### Statistical tests for targeted analyses

A two-sided Student’s *t* test was used to assess differences between means. A *p* value of 0.05 was considered statistically significant.

### Reporting summary

Further information on research design is available in the Nature Research Reporting Summary linked to this article.

## Supplementary information

Supplementary Information

Description of Additional Supplementary Files

Supplementary Dataset 1

Supplementary Dataset 2

Supplementary Dataset 3

Supplementary Dataset 4

Supplementary Dataset 5

Supplementary Dataset 6

Reporting Summary

## Data Availability

All raw mass spectrometric data files generated in this study have been deposited to the ProteomeXchange Consortium via the PRIDE partner repository^71^ with the dataset identifier PXD018744. The human Swiss-Prot database used for raw data search was downloaded from the UniProt database (https://www.uniprot.org/). RNAseq gene expression data used in Fig. 6b and Supplementary Fig. 10a were obtained from the CCLE database (Expression Public 19Q3 dataset) and were downloaded from the DepMap portal, by using the Data Explorer tool (https://depmap.org/portal/interactive). Gene expression data used in Supplementary Fig. 5a were downloaded from GEO using accession code GSE54380 (Gene expression in DND-41). Gene expression data used in Supplementary Fig. 6a were downloaded from GEO using accession code GSE123751 (PDTALL19 model). RNAseq gene expression data used in Supplementary Fig. 10b were downloaded from the supplementary information of Liu et al.^5^. Survival data used in Fig. 2g and Supplementary Fig. 4b were obtained from the Genomics of Drug Sensitivity in Cancer project (https://www.cancerrxgene.org), dataset GDSC1. Data used in Supplementary Fig. 9a, b were downloaded from the supplementary material of Klaeger et al.^52^. There are no restrictions on data availability. Source data are provided with this paper.

## Source data

Source Data
