# Supplementary material for: Proteomics of resistance to Notch1 inhibition in acute lymphoblastic leukemia reveals targetable kinase signatures
Source: Nat Commun. 2021 May 4;12:2507. doi: 10.1038/s41467-021-22787-9 (PMC8097059; doi:10.1038/s41467-021-22787-9)
Supplement: Supplementary file 1 — Supplementary Information [file 41467_2021_22787_MOESM1_ESM.pdf]

Supplementary information

Proteomics of resistance to Notch1 inhibition in acute lymphoblastic leukemia reveals targetable kinase signatures

Giulia Franciosa, Jos G. A. Smits, Sonia Minuzzo, Ana Martínez-Val, Stefano Indraccolo, Jesper V. Olsen

SUPPLEMENTARY METHODS

LC-MS/MS settings

Q-Exactive HF-X

Spray voltage was set to 2 kV, s-lens RF level at 40, and heated capillary temperature at 275 °C. Former target ions were dynamically excluded for 30 seconds and all experiments were acquired using positive polarity mode. Full scan resolution was set to 60,000 at m/z 200 and the mass range was set to m/z 350-1400. Full scan ion target value was 3E6 allowing a maximum fill time of 45 ms. For proteome analysis, 12 most intense precursors were selected for fragmentation. MS2 data was acquired at 15K resolution and an ion target value of 1E5, allowing maximum filling time of 22 ms. Higher-energy collisional dissociation (HCD) fragment scans was acquired with optimal setting for parallel acquisition using 1.3 m/z isolation width and normalized collision energy of 33. Phosphopeptide-enriched samples were analyzed with a sensitive top10 scanning method. Ion target value for HCD fragment scans were set to 1E5 with a maximum fill time of 86 ms and analyzed with 45,000 MS2 resolution. Only for DND-41 proteome experiment, MS/MS data was recorded in centroid mode (Phi-SDM).

Orbitrap Exploris 480

Intensity threshold was kept at 2E5. Isolation width was set at 1.3 m/z. Normalized collision energy was set at 30%. Data were acquired in DIA mode, as previously described21. Full MS resolution was set to 120,000 at m/z 200 and full MS AGC target was 300% with an IT of 45 ms. Mass range was set to 350–1400. AGC target value for fragment spectra was set at 1000%. 49 windows of 13.7 Da were used with an overlap of 1 Da. Resolution was set to 15,000 and IT to 22 ms. Normalized collision energy was set at 27%. All data were acquired in profile mode using positive polarity and peptide match was set to off, and isotope exclusion was on.

Settings for raw MS data processing

T-ALL proteome. MaxQuant version: 1.5.8.4. Human Swissprot database was downloaded in April 2017 and contained 21,042 entries. Maximum two variable modifications were allowed per peptide. Maximum three miscleavages were allowed per peptide. The “maximum peptide mass” was set to 7500 Da.

T-ALL phosphoproteome. MaxQuant version: 1.6.0.15. Human Swissprort database was downloaded in April 2017 and contained 21,042 entries.

DND-41 proteome. MaxQuant version: 1.6.1.11. Human Swissprot database was downloaded in January 2019 and contained 21,074 entries.

DND-41 phosphoproteome. MaxQuant version: 1.6.17.0. Human Swissprot database was downloaded in January 2019 and contained 21,074 entries.

ID count

MaxQuant

Peptides and phosphopeptides have been counted from the “modificationSpecificPeptides” table, after filtering out the reverse hits and rows without quantitative values. Protein groups have been counted from the “proteinGroups” table, after filtering out the reverse hits, proteins identified by only

modified peptides and rows without quantitative values. Phosphoproteins have been counted from the “modificationSpecificPeptides” by using the first Uniprot identifier of each protein group. Class-I phosphosites have been counted from the “Phospho (STY)Sites” table, after expanding the site table (in Perseus), filtering out the reverse hits, rows without quantitative values, and all sites with a localization probability >0.75. Sites identified on different peptides have been considered as one ID.

Spectronaut

To count peptides, we exported “EG.PrecursorId” and “EG.TotalQuantity”. To count protein groups, we exported “PG.ProteinGroups” and “EG.TotalQuantity”. To count phosphopeptides and phosphosites, we exported “EG.PrecursorId”, “EG.PTMAssyProbability” and “EG.TotalQuantity”. We thene generated a Perseus-compatible table by using our in-house R script implemented as Perseus plugin, as previously published [1].

Proteomics data normalization

T-ALL proteome

To remove the TMT batch effect, log2-transformed protein intensities were scaled per TMT/replicate using the median. Afterwards, we performed quantile-based normalization to correct for pipetting errors. Three valid values in at least one cell line were required for statistical analysis.

T-ALL phosphoproteome

After expanding the site table in Perseus, the common pooled channel was subtracted from each log2 intensity value, in order to remove the TMT batch effect. To remove a second batch effect due to biological replicates (collected in different days), log2-transformed protein intensities were scaled per replicate using the median. Afterwards, we performed quantile-based normalization to correct for pipetting errors. We only used class I phosphosites identified in all samples for the statistical analysis (100% valid values).

DND-41 proteome

To remove the batch effect due to biological replicates (collected in different days), log2-transformed protein intensities were scaled per replicate using the mean. Afterwards, we performed quantile-based normalization to correct for pipetting errors.

DND-41 phosphoproteome

After expanding the site table in Perseus, log2-transformed protein intensities were scaled per replicate using the mean, in order to remove a batch effect due to biological replicates (collected in different days). Afterwards, we performed quantile-based normalization to correct for pipetting errors.

PDTALL datasets

Data were normalized in Spectronaut (default settings). For the PDTALL19 resistant vs acute treatment, a further step of median normalization was also performed. Two samples (PDTALL19_aN1_AT_B and PDTALL11_aN1_4) were considered outliers and excluded from the statistical analysis. Three valid values in at least one condition were required for statistical analysis.

Normalized data and statistics for all datasets are provided as supplementary data.

MS2 spectra annotation

MS2 spectra annotation was performed in MS viewer, part of the Protein Prospector Web package [2]. Apl files from the MaxQuant combined/Andromeda folder were uploaded as one compressed folder. The msms.txt (from combined/txt folder) was uploaded after filtering out all spectra from proteins identified with >1 peptide. The data set can be accessed using the following URLs:

- T-ALL proteome. Search key is bgy8bdvegv.
- DND-41 proteome. Search key is 5opxpqh5iu.

Supplementary Table 1. Antibodies used for immunoblotting.

**Antibody**	**Brand**	**Cat. number**	**Dilution**
Cleaved Notch1 (Val1744)	Cell Signaling	4147	1:1000
Phospho-S6 Ribosomal Protein (Ser235/236)	Cell Signaling	4858	1:1000
S6 Ribosomal Protein	Santa Cruz Biotechnology	74459	1:500
Phospho-PKCδ (Thr505)	Cell Signaling	9374	1:1000
Phospho-PKCδ/θ (Ser643/676)	Cell Signaling	9376	1:1000
Phospho PKCδ (Ser299)	Abcam	133456	1:1000
PKCδ	Cell Signaling	9616	1:1000
Phospho-Akt (Ser473)	Cell Signaling	4060	1:1000
Phospho-Akt (Thr 308)	Cell Signaling	4056	1:1000
Akt	Cell Signaling	9272	1:1000
c-Myc	Cell Signaling	5605	1:1000
Cleaved caspase 3	Cell Signaling	9664	1:1000
Cleaved PARP	Cell Signaling	5625	1:1000
γ-Tubulin	Sigma-Aldrich	5326	1:5,000
Vinculin	Sigma-Aldrich	V9264	1:5000
β-Actin	Santa Cruz Biotechnology	47778	1:500
Anti-rabbit HRP conjugated secondary antibody	Jackson Immunoresearch	111-036-045	1:10000
Anti-mouse HRP conjugated secondary antibody	Jackson Immunoresearch	115-036-062	1:10000

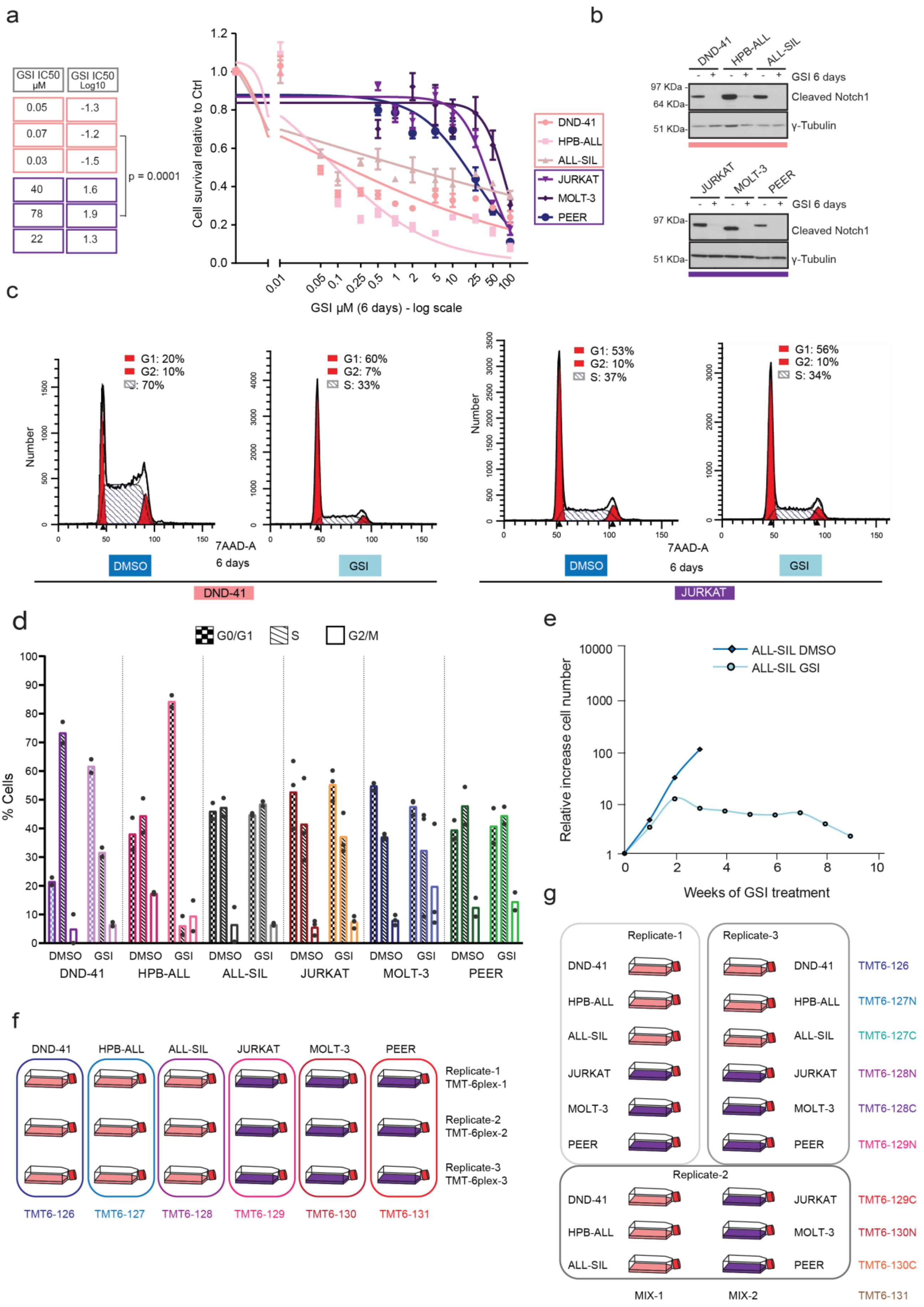
a
GSI IC50 μM
GSI IC50 Log10
0.05
-1.3
0.07
-1.2
0.03
-1.5
40
1.6
78
1.9
22
1.3
p = 0.0001
Cell survival relative to Ctrl
GSI μM (6 days) - log scale
0.01
0.05
0.1
0.25
0.5
1
2
5
10
25
50
100
DND-41
HPB-ALL
ALL-SIL
JURKAT
MOLT-3
PEER
b
DND-41
HPB-ALL
ALL-SIL
GSI 6 days
97 KDa
64 KDa
51 KDa
Cleaved Notch1
γ-Tubulin
JURKAT
MOLT-3
PEER
GSI 6 days
97 KDa
51 KDa
Cleaved Notch1
γ-Tubulin
c
G1: 20%
G2: 10%
S: 70%
G1: 60%
G2: 7%
S: 33%
G1: 53%
G2: 10%
S: 37%
G1: 56%
G2: 10%
S: 34%
Number
7AAD-A
6 days
DMSO
GSI
DND-41
JURKAT
d
G0/G1
S
G2/M
% Cells
DMSO
GSI
DND-41
HPB-ALL
ALL-SIL
JURKAT
MOLT-3
PEER
e
ALL-SIL DMSO
ALL-SIL GSI
Relative increase cell number
Weeks of GSI treatment
f
DND-41
HPB-ALL
ALL-SIL
JURKAT
MOLT-3
PEER
Replicate-1
TMT-6plex-1
Replicate-2
TMT-6plex-2
Replicate-3
TMT-6plex-3
TMT6-126
TMT6-127
TMT6-128
TMT6-129
TMT6-130
TMT6-131
g
Replicate-1
Replicate-3
Replicate-2
DND-41
HPB-ALL
ALL-SIL
JURKAT
MOLT-3
PEER
TMT6-126
TMT6-127N
TMT6-127C
TMT6-128N
TMT6-128C
TMT6-129N
TMT6-129C
TMT6-130N
TMT6-130C
TMT6-131
MIX-1
MIX-2

Supplementary Figure 1. Intrinsic sensitivity or resistance to Notch1 inhibition in T-ALL cell lines.

a. Cell survival of T-ALL cell lines in response to increasing doses of GSI (Compound E) for 6 days (right). The values shown are mean ± SEM (n=4-8 biologically independent samples examined over two independent experiments). Half maximal inhibitory concentration (IC50) was calculated using a non-linear regression in GraphPad Prism. An unpaired two-sided t test was used to assess the differences in mean IC50 (on -log transformed data) between the GSI resistant and sensitive T-ALLs (n=3 IC50 values). **b**. Immunoblot analysis of lysates from T-ALL cells for the indicated antibodies. The experiment was repeated at least 3-times. **c-d**. Flow cytometry analysis of 7AAD-stained T-ALL cells after six days of GSI treatment (0.5 µM). Representative histograms are presented only for DND-41 (GSI sensitive) and Jurkat (GSI resistant) cells. Data shown in D represent the mean between n=2 biologically independent experiments. Gating strategies are provided in Supplementary Fig. 12. **e**. Relative cell count performed by trypan blue exclusion of ALL-SIL cells treated with a fixed GSI dose of 0.1 µM up to 9 weeks. The experiment was repeated once. **f-g**. Experimental design of the TMT-labeling strategy used to perform proteomics (F) and phosphoproteomics analysis (G) in T-ALL cell lines. Source data are provided as Source Data file.

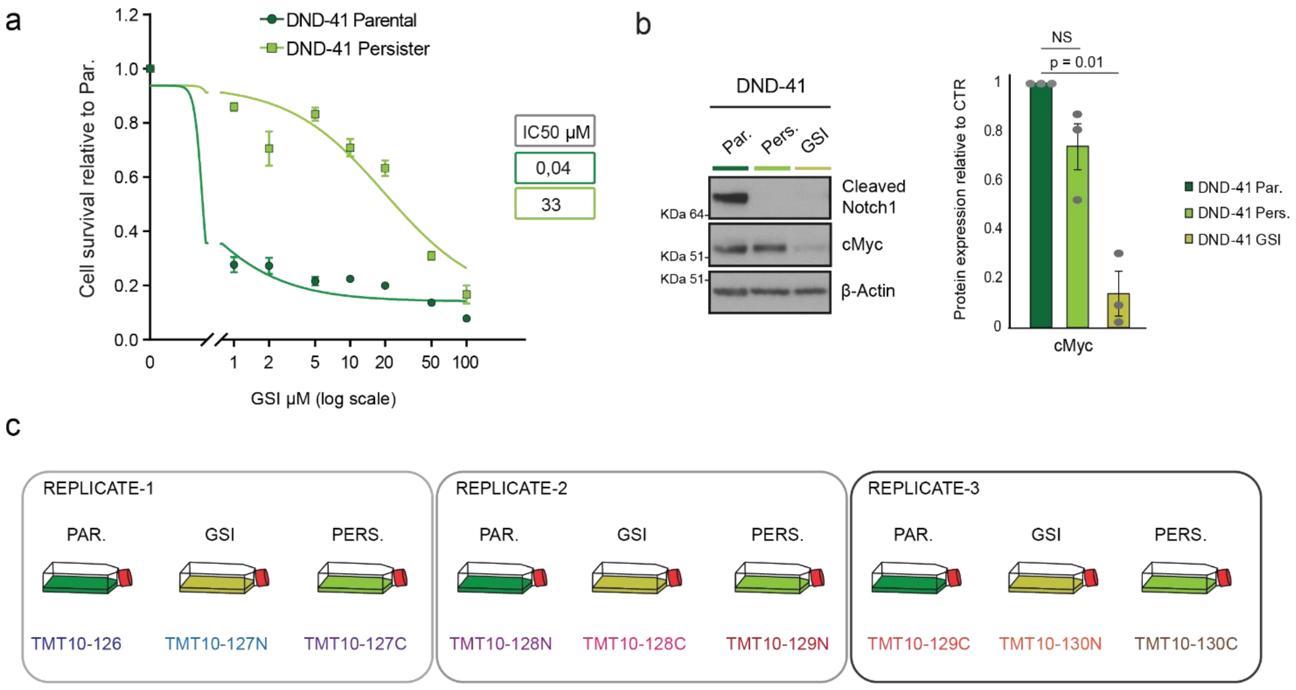

Supplementary Figure 2. **Generation of drug-tolerant “persister” DND-41 cells**.
a. Cell survival of parental and persister DND-41 cells in response to increasing doses of GSI for 6 days. The values shown are mean ± SEM (n=4 biologically independent samples examined over one experiment). Half maximal inhibitory concentration (IC50) was calculated using a non-linear regression in GraphPad Prism. **b**. Immunoblot analysis of lysates from DND-41 cells for the indicated antibodies. The values shown are mean ± SEM. An unpaired two-sided t test was used to assess the differences in protein expression (n=3 biologically independent experiments). Immunoblot quantification was performed in ImageJ. **c**. Experimental design of the TMT-labeling strategy used to perform proteomics and phosphoproteomics analysis in DND-41 cells. Par: parental; pers: persister; NS: non-significant. Source data are provided as Source Data file.

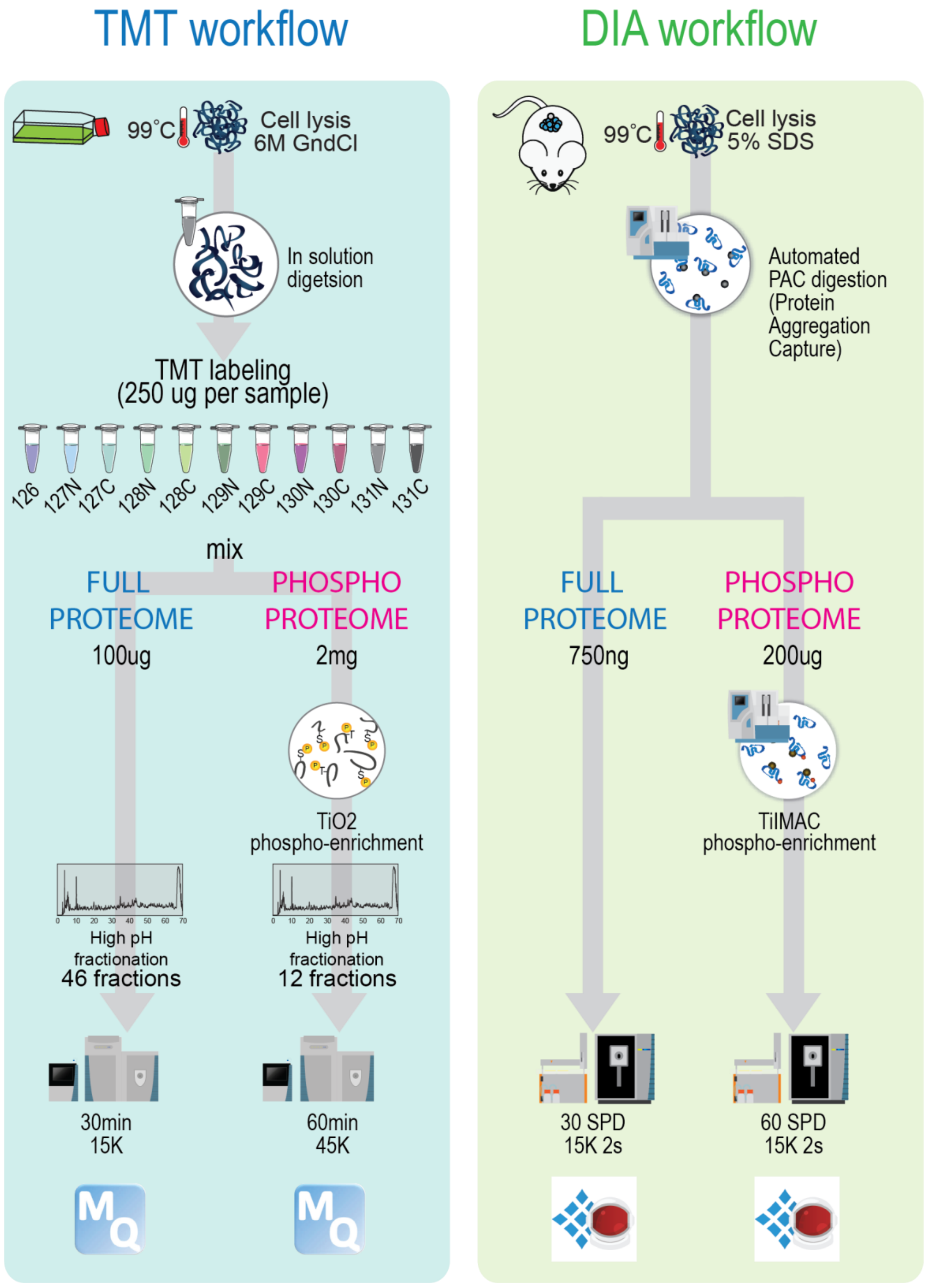

Supplementary Figure 3. **Proteomics and phosphoproteomics workflow**.
Datasets 1 and 2 have been generated by stable isotope labeling, using a tandem mass tag (TMT)-based workflow. Cells were lysed in a 6M guanidine hydrochloride (GndCl) lysis buffer and proteins were digested in-solution. After TMT labeling, the resulting peptides have been fractionated in 46 fractions for proteome analysis. Enrichment of phosphopeptides was performed through titanium dioxide (TiO2) and the resulting phosphopeptides were fractionated in 12 fractions. Samples have been analyzed on an EASY-nLC 1200 coupled with a Q-Exactive HFX mass spectrometer. The T-

ALL cell line proteome was analyzed in 30-min gradient per fraction, with a 15K MS2 resolution. DND-41 proteome was analyzed in Phi-SDM mode. Phosphoproteome fractions were analyzed in 60-min gradient per fraction, with a 45K MS2 resolution.

Dataset 3 was generated through a data independent acquisition (DIA) workflow. Cells were lysed in a 5% SDS lysis buffer and proteins were digested after aggregation on magnetic beads (PAC: protein aggregation capture) in a magnetic robot. Enrichment of phosphopeptides was performed through TiIMAC beads in a magnetic robot. Samples have been analyzed on the Evosep system (HPLC) coupled with an Orbitrap Exploris 480 mass spectrometer. Proteome samples have been analyzed label-free on a 44-min gradient (30 SPD = samples per day) and phosphoproteome samples were run on a 21-min gradient (60 SPD). For all samples a MS2 resolution of 15K and a cycle time of 2 seconds were used.

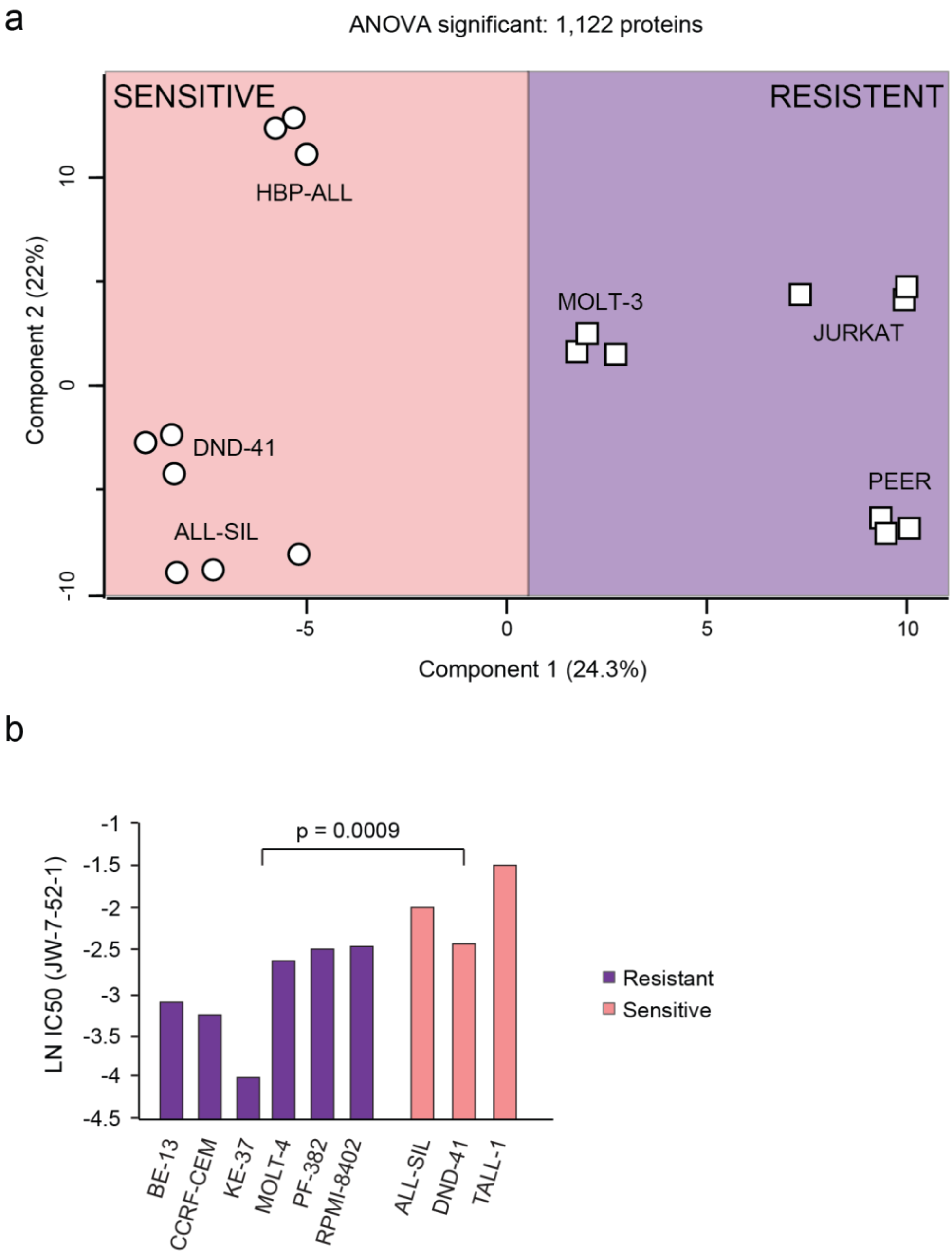

Supplementary Figure 4. Proteomics analysis of T-ALL cell lines.

a. Principal component analysis (PCA) of significantly regulated proteins among the 6 cell lines (one-way ANOVA: permutation-based FDR <0.01; s0=0.1; n=3 biologically independent experiments). **b**. Sensitivity values (natural logarithm, LN, of the half maximal inhibitory concentration, IC50, in µM) of 9 human T-ALL cell lines treated with the mTOR inhibitor JW-7-52-1. An unpaired two-sided t test was used to assess the differences in mean LN (IC50) between the GSI resistant (n=6) and sensitive (n=3) T-ALL cell lines. Data were obtained from the Genomics of Drug Sensitivity in Cancer project (GDSC1). Source data are provided as Source Data file.

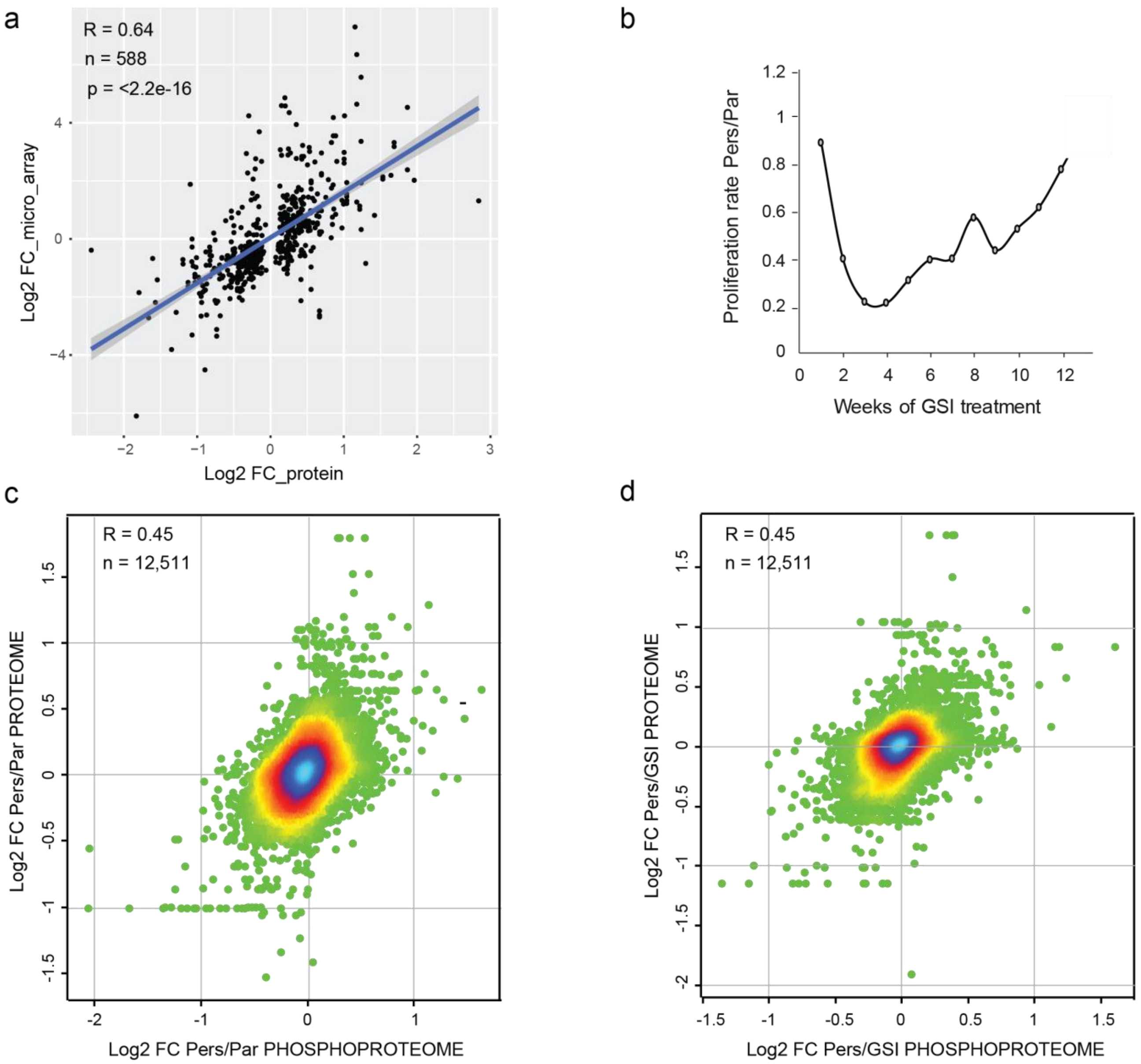

Supplementary Figure 5. Proteomics and phosphoproteomics analysis of drug-tolerant "persister" DND-41 cells.

a. Two-tailed linear regression analysis for proteins significantly regulated in the persister cell proteome dataset (Persister-parental comparison; SAM test with Benjamini-Hochberg FDR <0.01, S0=0.1 and n=3 biologically independent experiments) and genes reliably measured in the microarray dataset (detection p value <0.05). The grey shading represents the 95% confidence interval of the regression line. Microarray data were downloaded from the GEO database. **b**. Proliferation rate of DND-41 persister cells relative to parental cells over long-term GSI treatment (12 weeks). Cell count was determined by trypan blue exclusion. The experiment was performed once. **c-d**. Linear regression analysis between log2 of the fold-change persister-parental (C) or persister-GSI (D) in the phosphoproteome (x axis) and the proteome (y axis). R=Pearson correlation coefficient; par: parental; pers: persister. Source data are provided as Source Data file.

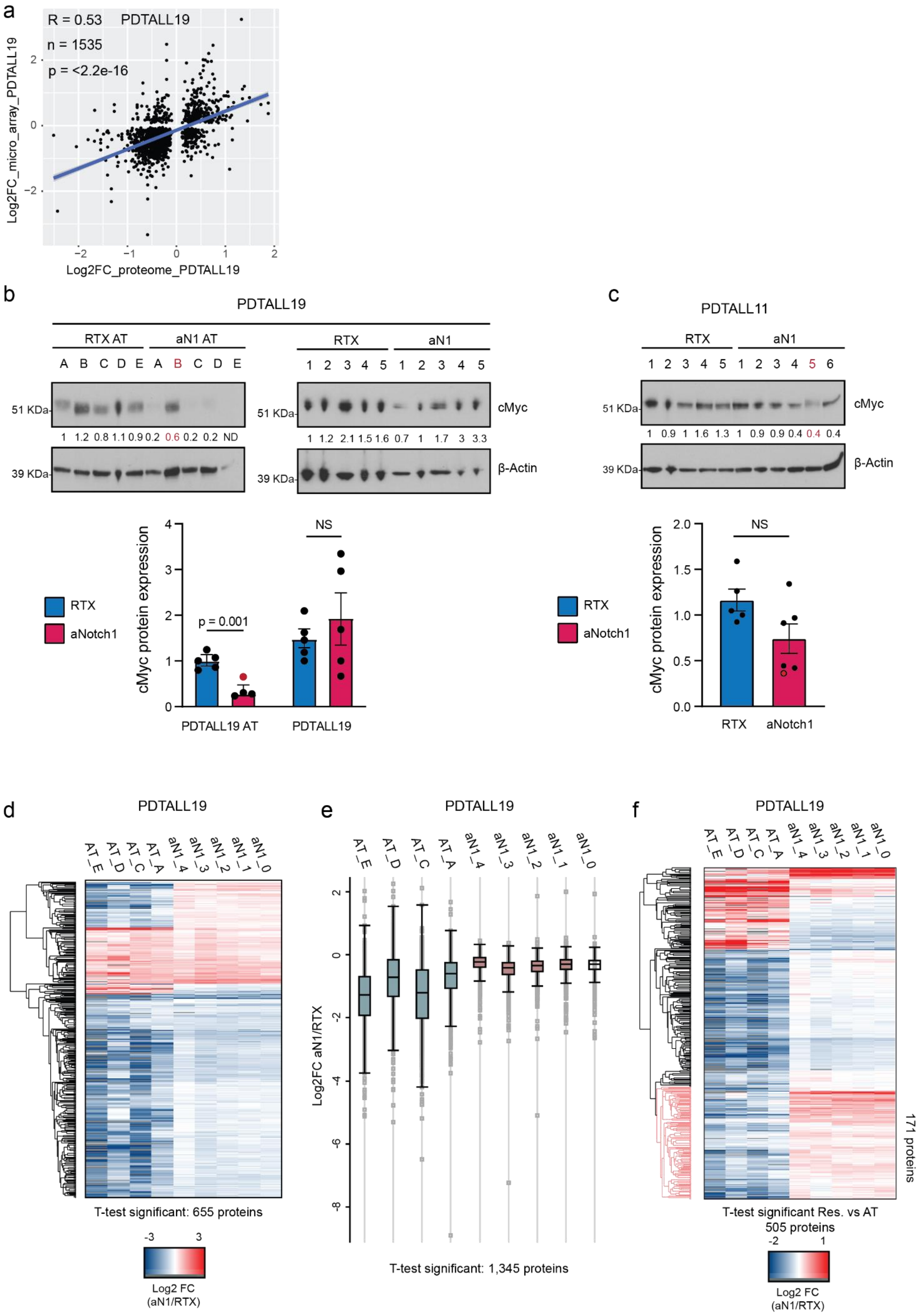
a
R = 0.53 PDTALL19
n = 1535
p = <2.2e-16
Log2FC_micro_array_PDTALL19
Log2FC_proteome_PDTALL19
b
PDTALL19
RTX AT
aN1 AT
RTX
aN1
A B C D E A B C D E
1 2 3 4 5 1 2 3 4 5
51 KDa
39 KDa
cMyc
β-Actin
1 1.2 0.8 1.1 0.9 0.2 0.6 0.2 0.2 ND
1 1.2 2.1 1.5 1.6 0.7 1 1.7 3 3.3
RTX
aNotch1
cMyc protein expression
p = 0.001
NS
PDTALL19 AT
PDTALL19
c
PDTALL11
RTX
aN1
1 2 3 4 5 1 2 3 4 5 6
51 KDa
39 KDa
cMyc
β-Actin
1 0.9 1 1.6 1.3 1 0.9 0.9 0.4 0.4 0.4
RTX
aNotch1
cMyc protein expression
NS
RTX
aNotch1
d
PDTALL19
AT_E AT_D AT_C AT_A aN1_4 aN1_3 aN1_2 aN1_1 aN1_0
T-test significant: 655 proteins
-3 3
Log2 FC
(aN1/RTX)
e
PDTALL19
AT_E AT_D AT_C AT_A aN1_4 aN1_3 aN1_2 aN1_1 aN1_0
Log2FC aN1/RTX
T-test significant: 1,345 proteins
f
PDTALL19
AT_E AT_D AT_C AT_A aN1_4 aN1_3 aN1_2 aN1_1 aN1_0
171 proteins
T-test significant Res. vs AT
505 proteins
-2 1
Log2 FC
(aN1/RTX)

Supplementary Figure 6. Proteomics analysis of T-ALL PDX resistant to anti-Notch1 monoclonal antibody OMP52M51.

a. Two-tailed linear regression analysis for proteins significantly regulated in the PDTALL19 proteome dataset (aNotch1-RTX; Benjamini-Hochberg FDR <0.05; S0=0.1; n=5 biologically independent mice) and genes significantly regulated in the microarray dataset (significance analysis was performed in GEO2R: p value <0.05). R=Pearson correlation coefficient. The grey shading represents the 95% confidence interval of the regression line. Microarray data were downloaded from the GEO database. **b-c**. Immunoblot analysis of lysates from PDTALL19 (B) and PDTALL11 (C) cells for the indicated antibodies. The values shown are mean ± SEM (PDTALL11: RTX: n=5 biologically independent mice; aN1: n=6. PDTALL19: n=5; PDTALL19 acute treatment, AT: RTX: n=5; aN1: n=4, since sample E was excluded). An unpaired two-sided t test was used to assess the differences in protein expression. Samples labeled in red represent the outliers in the proteome data. Immunoblot quantification was performed in ImageJ. **d**. Shared regulated proteins in both PDTALL19 and PDTALL19 AT are represented in a heatmap. Significance analysis was performed for aN1/RTX and aN1_AT/RTX by unpaired two-sided t test with permutation-based FDR <0.05. **e**. Boxplot of log2 fold-changes aN1/RTX and aN1_AT/RTX. The upper and lower whiskers represent values outside the middle 50%. The end of the lower whisker represents the minimum intensity value. The end of the upper one represents the maximum. The median marks the mid-point of the data. **f**. Log2 fold-changes aN1/RTX and aN1_AT/RTX of proteins whose log2 fold-changes are significantly different between PDTALL19 (n=5 biologically independent mice) and PDTALL19 acute treatment (n=4; unpaired two-sided SAM test with Benjamini-Hochberg FDR <0.05, s0=0.1). RTX: Rituximab; aN1: anti-Notch1; AT: acute treatment with anti-Notch1 antibody; NS: non-significant. Source data are provided as Source Data file.

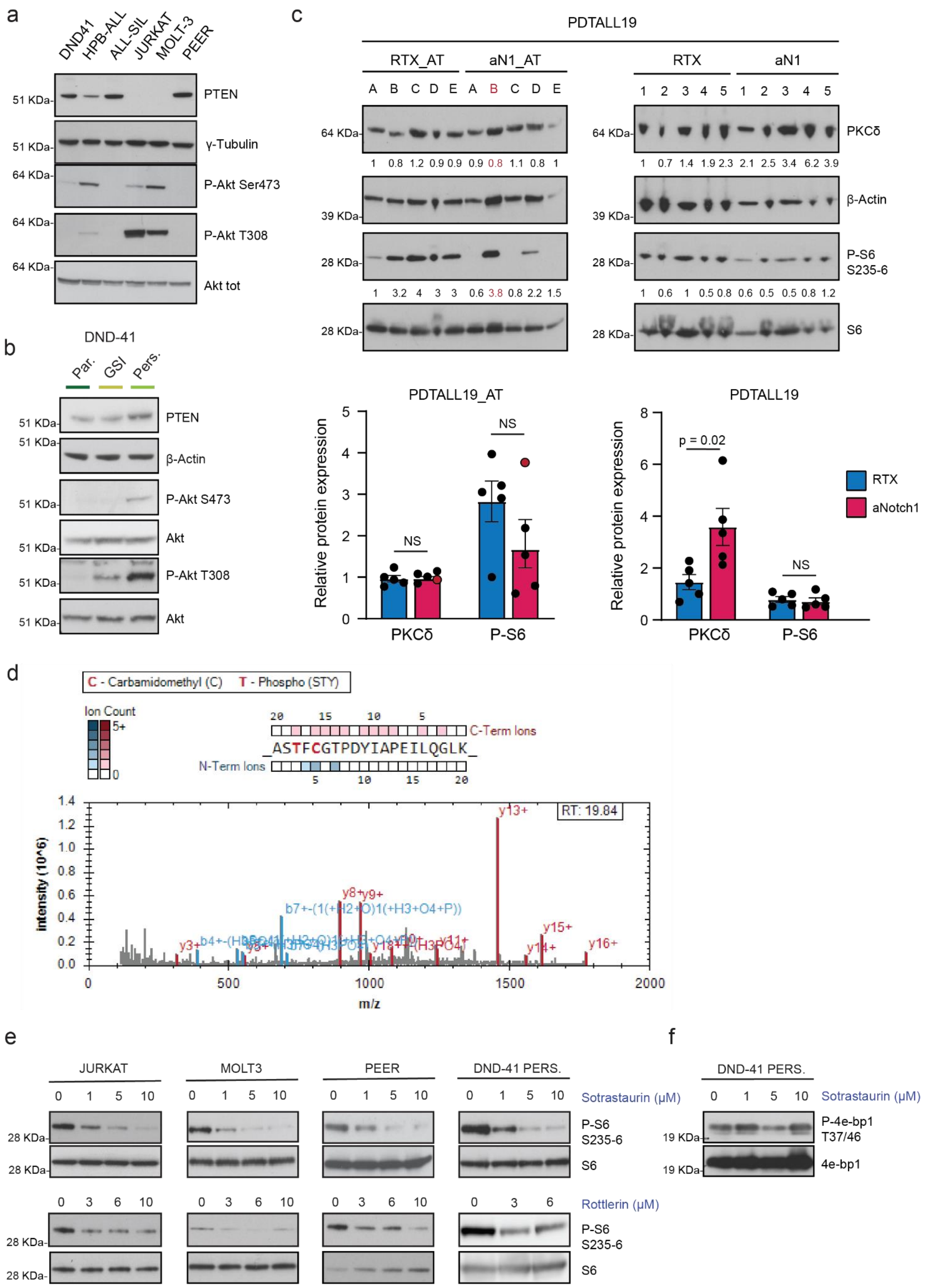
a
DND41
HPB-ALL
ALL-SIL
JURKAT
MOLT-3
PEER
51 KDa-
PTEN
51 KDa-
γ-Tubulin
64 KDa-
P-Akt Ser473
64 KDa-
P-Akt T308
64 KDa-
Akt tot
b
DND-41
Par.
GSI
Pers.
51 KDa-
PTEN
51 KDa-
β-Actin
51 KDa-
P-Akt S473
51 KDa-
Akt
51 KDa-
P-Akt T308
51 KDa-
Akt
c
PDTALL19
RTX_AT
aN1_AT
RTX
aN1
A B C D E A B C D E
1 2 3 4 5 1 2 3 4 5
64 KDa-
PKCδ
1 0.8 1.2 0.9 0.9 0.9 0.8 1.1 0.8 1
1 0.7 1.4 1.9 2.3 2.1 2.5 3.4 6.2 3.9
39 KDa-
β-Actin
28 KDa-
P-S6
S235-6
1 3.2 4 3 3 0.6 3.8 0.8 2.2 1.5
1 0.6 1 0.5 0.8 0.6 0.5 0.5 0.8 1.2
28 KDa-
S6
PDTALL19_AT
Relative protein expression
NS
NS
PKCδ
P-S6
PDTALL19
Relative protein expression
p = 0.02
NS
PKCδ
P-S6
RTX
aNotch1
d
C - Carbamidomethyl (C) T - Phospho (STY)
Ion Count
5+
0
C-Term Ions
ASTFCGTPDYIAPEILQGLK
N-Term Ions
RT: 19.84
y13+
y8+y9+
b7+-(1(+H2+O)1(+H3+O4+P))
y15+
y16+
y14+
y3+
intensity (10^6)
m/z
e
JURKAT
MOLT3
PEER
DND-41 PERS.
0 1 5 10
Sotrastaurin (μM)
28 KDa-
P-S6
S235-6
28 KDa-
S6
0 3 6 10
Rottlerin (μM)
28 KDa-
P-S6
S235-6
28 KDa-
S6
f
DND-41 PERS.
0 1 5 10
Sotrastaurin (μM)
19 KDa-
P-4e-bp1
T37/46
19 KDa-
4e-bp1

Supplementary Figure 7. **Kinase signature associated with resistance to NOTCHi reveals PKC activation in resistant cells**.

a-b. Immunoblot analysis of lysates from T-ALL cells for the indicated antibodies. The blot shown in a is representative of n=2 biologically independent experiments. The experiment in b was repeated once. **c**. Immunoblot analysis of lysates from PDTALL19 cells for the indicated antibodies. The values shown are mean ± SEM (n=5 biologically independent mice). An unpaired two-sided t test was used to assess the differences in protein expression. Samples labeled in red represent the outliers in the proteome data. If the N1_AT B sample were excluded for P-S6: p = 0.05. Immunoblot quantification was performed in ImageJ. **d**. MS/MS spectrum of the phosphorylated T07 peptide of PKCδ. **e-f**. T-ALL cells were treated for 30 minutes with the PKCδ inhibitors sotrastaurin or rottlerin at the indicated doses and the lysates were immunoblotted for the indicated antibodies. The experiment was performed once. RTX: Rituximab; aN1 or aNotch1: anti-Notch1; AT: acute treatment with anti-Notch1 antibody; NS: non-significant. Source data are provided as Source Data file.

a

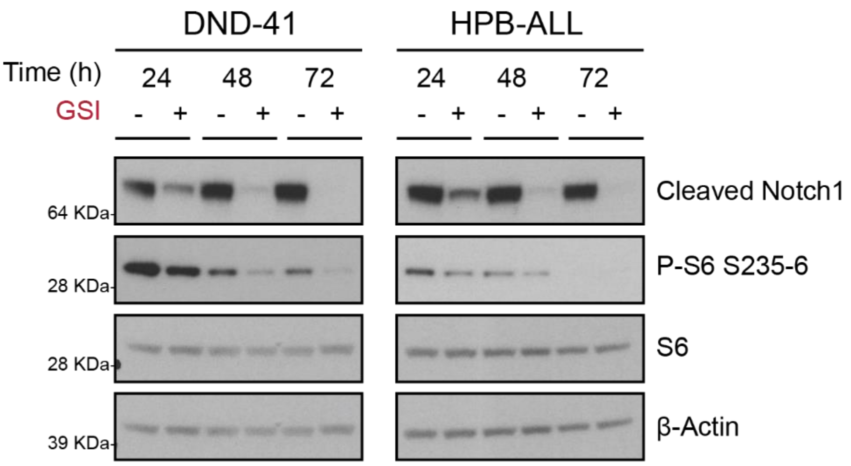

b

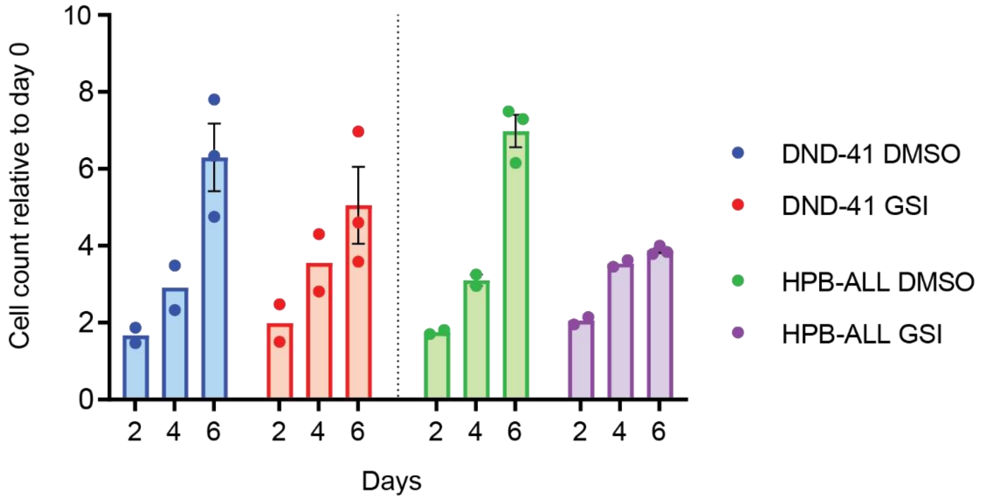

Supplementary Figure 8. **Short-term NOTCHi by GSI in the sensitive cell lines DND-41 and HPB-ALL**.

a. The T-ALL cell lines DND-41 and HPB-ALL were treated for 24-48-72 hours with GSI (0.5 µM) and the lysates were immunoblotted with the indicated antibodies. The experiment was performed once. **b**. Cell count relative to day 0, performed by trypan blue exclusion, of DND-41 and HPB-ALL cells treated with a GSI (0.5 µM). The data shown represent mean ± SEM (2, 4 days: n=2; 6 days: n=3 biologically independent experiments). Source data are provided as Source Data file.

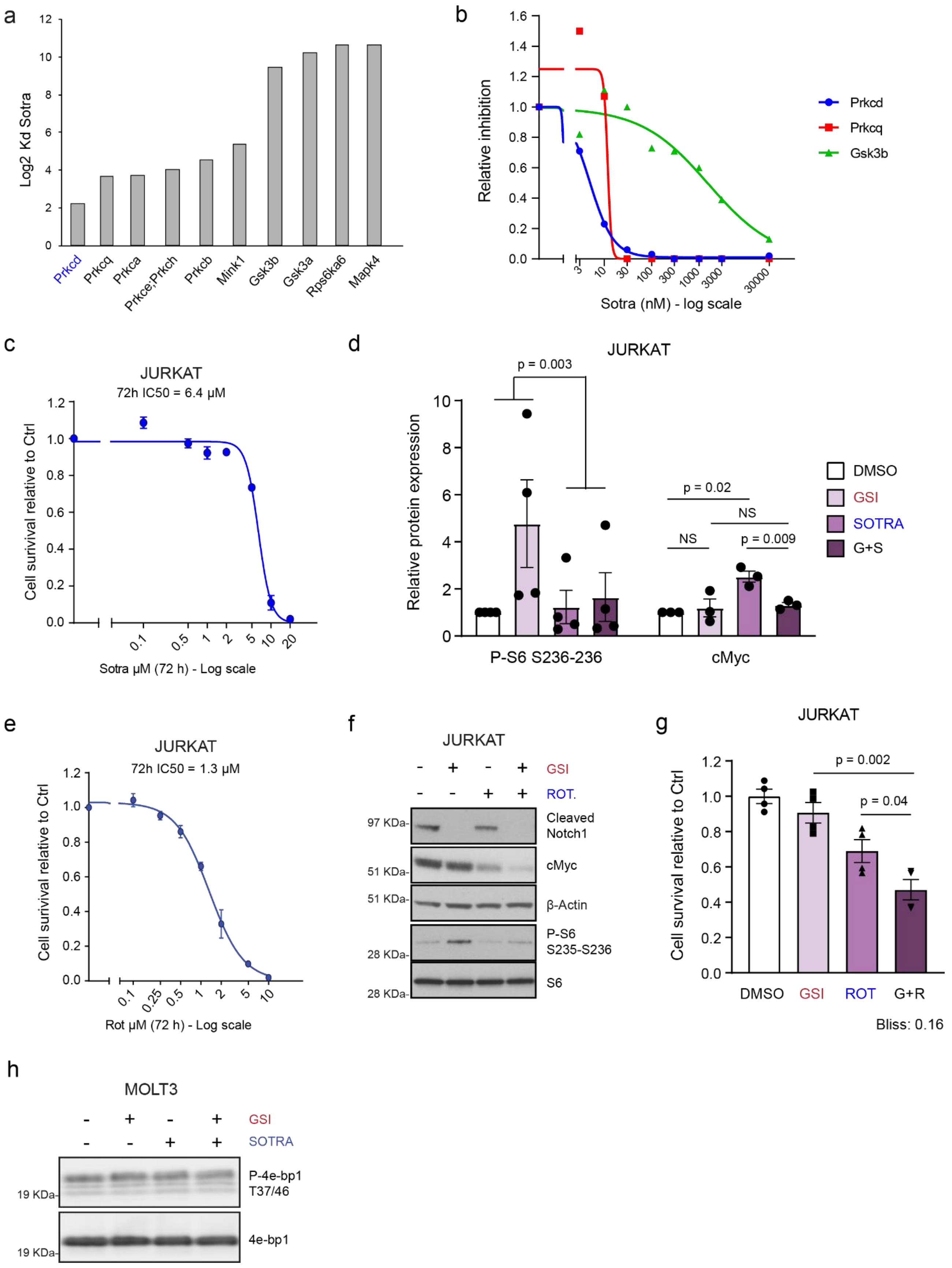

Supplementary Figure 9. **Combination treatment targeting Notch and PKC in resistant cells**. **a**. Sotrastaurin dissociation constant (Kd) of the drug-protein interaction for all the high confidence sotrastaurin targets. Kd is directly proportonial to the EC50, that is the effective drug concentration

at which half of the target protein is bound to the drug. **b**. Sotrataurin inhibition profiles for two on-targets (Prkcd and Prkcq) and one off-target (Gsk3b). Data in (A) and (B) were obtained from Klaeger at al. **c**. Cell survival of JURKAT cells treated with increasing doses of sotrastaurin for 72 hours. The values shown are mean ± SEM (n=8 biologically independent samples examined over 2 independent experiments). Half maximal inhibitory concentration (IC50) was calculated using a non-linear regression in GraphPad Prism. **d**. Quantification of the blot of Fig. 7c. The values shown are mean ± SEM (n=4 biologically independent experiments for P-S6; n=3 for cMyc). A two-sided t test was used to assess the differences in mean protein expression (paired for P-S6, unpaired for cMyc). Immunoblot quantification was performed in ImageJ. **e**. Cell survival assay of Jurkat cells in response to increasing doses of rottlerin for 72 hours. The values shown are mean ± SEM (n=8 biologically independent samples examined over 2 independent experiments). Half inhibitory concentration (IC50) was calculated using a non-linear regression in GraphPad Prism. **f**. JURKAT cells were treated for 3 days with GSI (0.5 μM), rottlerin (0.75 μM) or the combination, and the lysates were immunoblotted for the indicated antibodies. The blot is representative of two independent experiments. **g**. JURKAT cells were treated for 6 days with GSI (0.5 μM), rottlerin (0.75 μM) or the combination, and they were assayed for cell survival. The values shown are mean ± SEM (n=4 biologically independent samples examined over 2 independent experiments). An unpaired two-sided t test was used to assess the differences in mean survival. Bliss>0 = synergy; <0 = antagonism; =0 = additive effect. **h**. MOLT-3 cells were treated for 3 days with GSI (0.5 μM), sotrastaurin (5 μM) or the combination, and the lysates were immunoblotted for the indicated antibodies. The experiment was performed once. Sotra: sotrastaurin. NS: non-significant. Source data are provided as Source Data file.

a

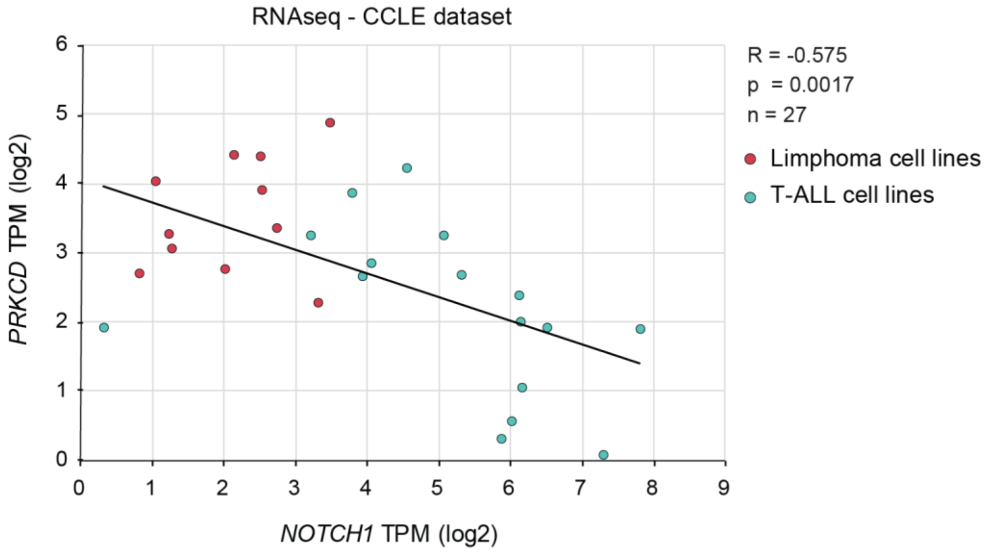

b

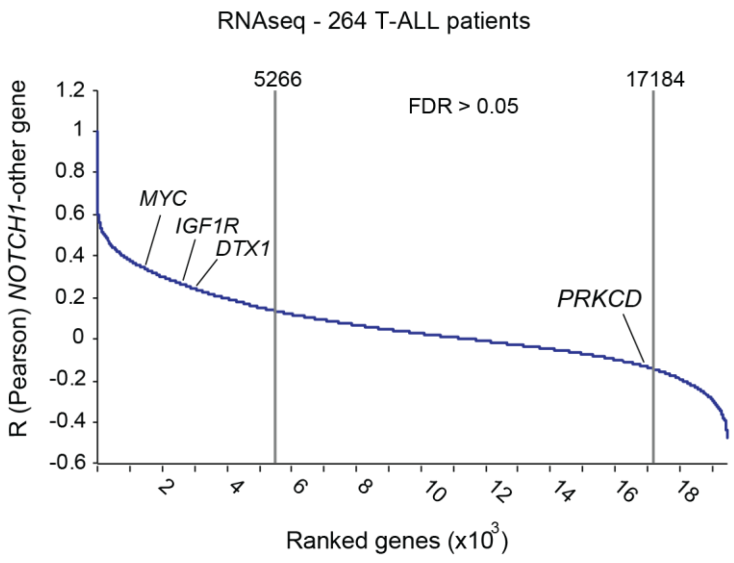

Gene	Rank	R	FDR
MYC	1723	0.32	9.7E-07
IGF1R	2390	0.28	2.9E-05
DTX1	2677	0.26	1E-05
PRKCD	17126	-0.14	0.055

Supplementary Figure 10. ***NOTCH1* and *PKCδ* gene expression are mutually exclusive in T-ALL cell lines and a cohort of 264 T-ALL patients**.

a. Two-tailed linear regression analysis of *NOTCH1* and *PKCδ* gene expression in a T-ALL cell line panel (RNA-seq data). Data were taken from the CCLE database (Expression Public 21Q1 dataset). TPM=Transcripts Per Million. R=Pearson correlation coefficient. **b**. Two-tailed linear regression analysis between *NOTCH1* gene expression and all the other genes in a cohort of 264 T-ALL patients (RNA-seq data). Pearson correlation coefficient values are plotted on the y axis and genes are ranked by Pearson correlation coefficient on the x axis. Significance of the correlation (p value) was corrected by Benjamini-Hochberg procedure and the significant gene correlations are those outside the black lines. Values in the table on the right represent rank, Pearson correlation coefficient and corrected p value (FDR) for three known Notch1 target genes (positive correlation with Notch1) and *PKCδ*. Source data are provided as Source Data file.

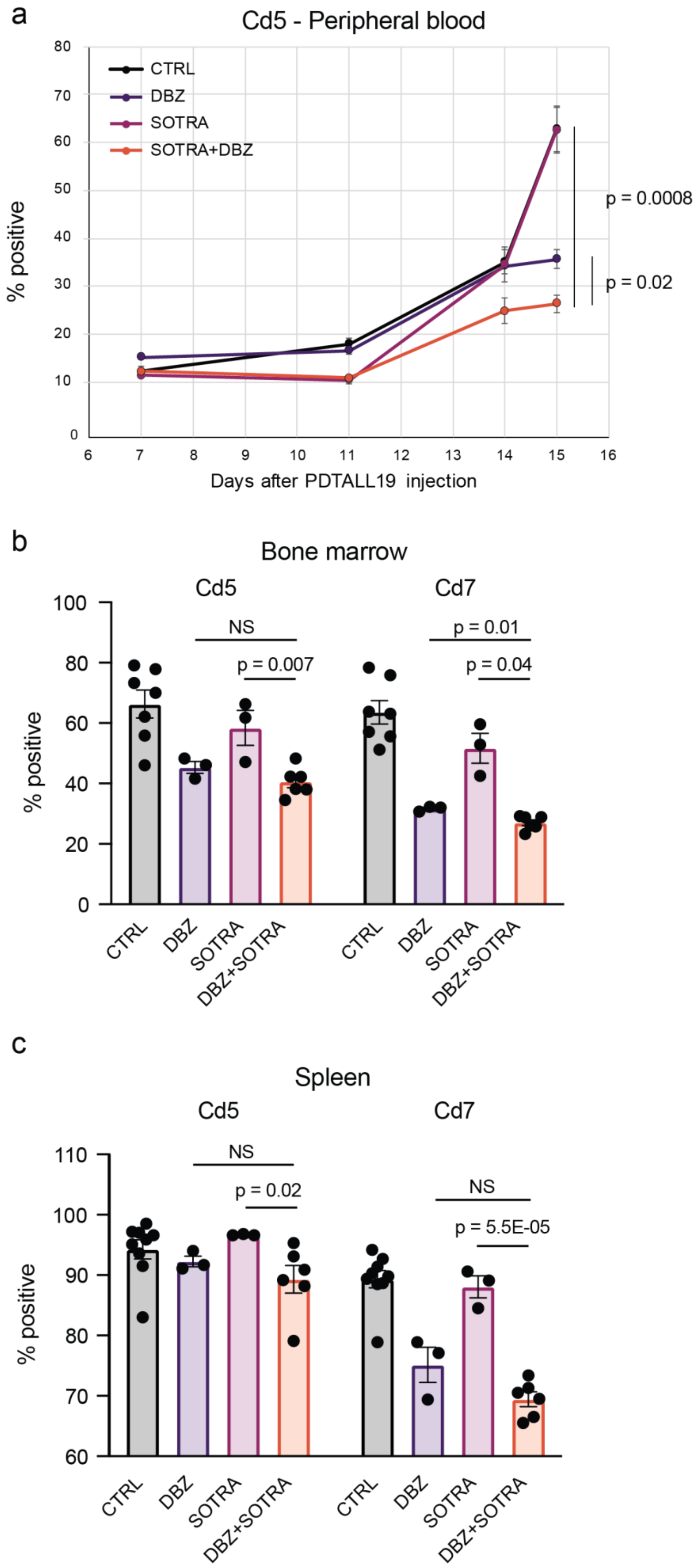
a
Cd5 - Peripheral blood
CTRL
DBZ
SOTRA
SOTRA+DBZ
% positive
80
70
60
50
40
30
20
10
0
p = 0.0008
p = 0.02
6
7
8
9
10
11
12
13
14
15
16
Days after PDTALL19 injection
b
Bone marrow
Cd5
Cd7
100
80
60
40
20
0
% positive
NS
p = 0.007
p = 0.01
p = 0.04
CTRL
DBZ
SOTRA
DBZ+SOTRA
CTRL
DBZ
SOTRA
DBZ+SOTRA
c
Spleen
Cd5
Cd7
110
100
90
80
70
60
% positive
NS
p = 0.02
NS
p = 5.5E-05
CTRL
DBZ
SOTRA
DBZ+SOTRA
CTRL
DBZ
SOTRA
DBZ+SOTRA

Supplementary Figure 11. **Combination treatment targeting Notch and PKC in PDTALL19 cells after engraftment in NOD/SCID mice**.

a. NOD/SCID mice were injected with $1x10^6$ PDTALL19 cells on day 1. On day 7, the mice were randomized and treated with vehicle (Ctrl), DBZ (GSI), sotrastaurin or sostrastaurin+DBZ. DBZ was administered in a four days on/three days off regimen (four doses in total). Sotrastaurin was administered in a regimen of 5 days on/2 days off (five doses in total). Flow cytometry analysis of human $CD5^+$ cells in peripheral blood was performed on day 7, 11, 14 and 15 (sacrifice day). The values showed are mean ± SEM. Significance was assessed by unpaired two-sided t test. Vehicle n=6 biologically independent mice; DBZ n=3; sotrastaurin n=5; sotrastaurin+DBZ n=6 biologically independent mice. **b**. Flow cytometry analysis of human $CD5^+$ and $CD7^+$ cells in bone marrow. The values showed are mean ± SEM. Significance was assessed by unpaired two-sided t test. Vehicle n=7 biologically independent mice; DBZ n=3; sotrastaurin n=3; sotrastaurin+DBZ n=6. **c**. Flow cytometry analysis of human $CD5^+$ and $CD7^+$ cells in spleen. The values showed are mean ± SEM. Significance was assessed by two-sided t test. Vehicle n=10 biologically independent mice; DBZ n=3; sotrastaurin n=3; sotrastaurin+DBZ n=6. NS: non-significant, sotra: sotrastaurin. Gating strategies are provided in Supplementary Fig. 12. Source data are provided as Source Data file.

a

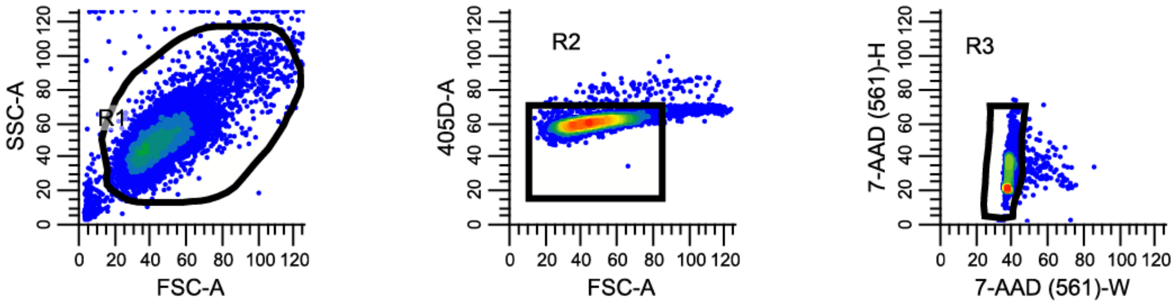

b

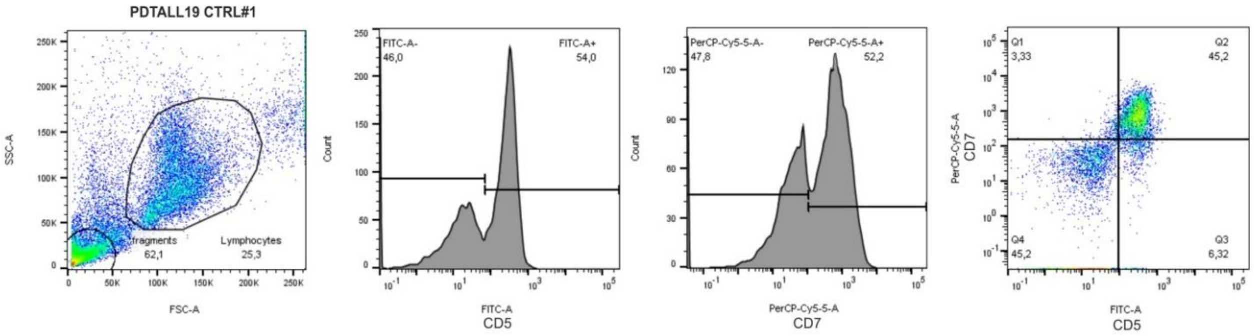

Supplementary Figure 12. **Gating strategies**.

a. Gating strategy for the cell cycle analysis shown in Supplementary Fig. 1c-d. The population shown in the plot on the right (R3) was used for analysis. One representative sample is shown. **b**. Left plot: gating strategy for analysis of human T-ALL cells in peripheral blood, spleen and bone marrow. The population labeled as Lymphocytes was used for analysis. Right plots: analysis strategy to measure CD5$^+$ and CD7$^+$ cells. One representative sample is shown.

SUPPLEMENTARY REFERENCES

1. Bekker-Jensen, D. B. *et al.* Rapid and site-specific deep phosphoproteome profiling by data-independent acquisition without the need for spectral libraries. *Nat. Commun.* **11**, 787 (2020).
2. Baker, E. S. *et al.* Advancing the high throughput identification of liver fibrosis protein signatures using multiplexed ion mobility spectrometry. *Mol. Cell. Proteomics* **13**, 1119–1127 (2014).
