# Supplementary material for: Proteomics of resistance to Notch1 inhibition in acute lymphoblastic leukemia reveals targetable kinase signatures
Source: Nat Commun. 2021 May 4;12:2507. doi: 10.1038/s41467-021-22787-9 (PMC8097059; doi:10.1038/s41467-021-22787-9)
Supplement: Supplementary file 2 — Description of Additional Supplementary Files [file 41467_2021_22787_MOESM2_ESM.docx]

Supplementary data 1. Information about the T-ALL cell lines used in the publication.

Supplementary data 2. T-ALL cell line (model n. 1) proteome and phosphoproteome (normalized data), including the results of the significance analysis.

Supplementary data 3. DND-41 (model n. 2) proteome and phosphoproteome (normalized data), including the results of the significance analysis.

Supplementary data 4. Results of the Fisher’s exact test performed on each of the six clusters displayed in Fig. 3b-c.

Supplementary data 5. PDTALL (model n. 3) proteome and phosphoproteome (normalized data), including the results of the significance analysis.

Supplementary data 6. Kinase-substrate enrichment analysis (KSEA) output.
