# Supplementary material for: Proteomics of resistance to Notch1 inhibition in acute lymphoblastic leukemia reveals targetable kinase signatures
Source: Nat Commun. 2021 May 4;12:2507. doi: 10.1038/s41467-021-22787-9 (PMC8097059; doi:10.1038/s41467-021-22787-9)
Supplement: Supplementary file 9 — Reporting Summary [file 41467_2021_22787_MOESM9_ESM.pdf]

nature research

Corresponding author(s): Jesper Velgaard Olsen

Last updated by author(s): Mar 8, 2021

Reporting Summary

Nature Research wishes to improve the reproducibility of the work that we publish. This form provides structure for consistency and transparency in reporting. For further information on Nature Research policies, see our Editorial Policies and the Editorial Policy Checklist.

Statistics

For all statistical analyses, confirm that the following items are present in the figure legend, table legend, main text, or Methods section.

n/a	Confirmed	
☐	☒	The exact sample size (*n*) for each experimental group/condition, given as a discrete number and unit of measurement
☐	☒	A statement on whether measurements were taken from distinct samples or whether the same sample was measured repeatedly
☐	☒	The statistical test(s) used AND whether they are one- or two-sided<br>*Only common tests should be described solely by name; describe more complex techniques in the Methods section.*
☒	☐	A description of all covariates tested
☐	☒	A description of any assumptions or corrections, such as tests of normality and adjustment for multiple comparisons
☐	☒	A full description of the statistical parameters including central tendency (e.g. means) or other basic estimates (e.g. regression coefficient) AND variation (e.g. standard deviation) or associated estimates of uncertainty (e.g. confidence intervals)
☐	☒	For null hypothesis testing, the test statistic (e.g. *F*, *t*, *r*) with confidence intervals, effect sizes, degrees of freedom and *P* value noted<br>*Give P values as exact values whenever suitable.*
☒	☐	For Bayesian analysis, information on the choice of priors and Markov chain Monte Carlo settings
☒	☐	For hierarchical and complex designs, identification of the appropriate level for tests and full reporting of outcomes
☐	☒	Estimates of effect sizes (e.g. Cohen's *d*, Pearson's *r*), indicating how they were calculated

Our web collection on statistics for biologists contains articles on many of the points above.

Software and code

Policy information about availability of computer code

Data collection	Xcalibur 3.1-4, Chronos 4.9.2.0, Chromeleon 6.8, BD FACSDiva 8-9
Data analysis	MaxQuant 1.5.8.4-1.6.17, Perseus 1.6.5-1.6.14, Spectronaut 8, R 4-4.0.3, R studio 1.2, Graphpad prism 7-9, Cytoscape 3.7-3.8, Microsoft Excel 2016, ImageJ 1.53, GIMP 2.10, Adobe Illustrator CS6-CC-2020, ModFit LT V.4.1.7, Enrichr (January 23rd 2019 release), CLUE 1.1, stringApp 1.5-1.6, clusterMaker2 1.3.1, KSEAapp 0.99.0

For manuscripts utilizing custom algorithms or software that are central to the research but not yet described in published literature, software must be made available to editors and reviewers. We strongly encourage code deposition in a community repository (e.g. GitHub). See the Nature Research guidelines for submitting code & software for further information.

Data

Policy information about availability of data

All manuscripts must include a data availability statement. This statement should provide the following information, where applicable:

- Accession codes, unique identifiers, or web links for publicly available datasets
- A list of figures that have associated raw data
- A description of any restrictions on data availability

All raw mass spectrometric data files generated in this study have been deposited to the ProteomeXchange Consortium via the PRIDE partner repository with the dataset identifier PXD018744.
The human Swiss-Prot database used for raw data search was downloaded from the UniProt database [https://www.uniprot.org/uniprot/?query=*&fil=organism%3A%22Homo+sapiens+%28Human%29+%5B9606%5D%22+AND+reviewed%3Ayes].
RNAseq gene expression data used in Fig. 6b and S10a were obtained from the CCLE database (Expression Public 19Q3 dataset) and were downloaded from the DepMap portal, by using the “Data Explorer” tool [https://depmap.org/portal/interactive].

Gene expression data used in Fig. S5a were downloaded from GEO [https://www.ncbi.nlm.nih.gov/geo] (accession number GSE54380). Gene expression data used in Fig. S6a were downloaded from GEO (accession number GSE123751).
RNAseq gene expression data used in Fig. S10b were downloaded from the supplementary information of the Liu et al. (table S5).
Survival data used in Fig. 2g and S4b were obtained from the Genomics of Drug Sensitivity in Cancer project [https://www.cancerrxgene.org], dataset GDSC1.
Data used in Fig. S9a-b were downloaded from the supplementary material of Klaeger et al. (tables S2 and S6).
Source data are provided with this paper. There are no restrictions on data availability.

Field-specific reporting

Please select the one below that is the best fit for your research. If you are not sure, read the appropriate sections before making your selection.

- [x] Life sciences
- [ ] Behavioural & social sciences
- [ ] Ecological, evolutionary & environmental sciences

For a reference copy of the document with all sections, see nature.com/documents/nr-reporting-summary-flat.pdf

Life sciences study design

All studies must disclose on these points even when the disclosure is negative.

Sample size	2-10 biological replicates were chosen based on the user's experience, in order to obtain sufficient downstream statistical power. No statistical method to calculate sample size was used.
Data exclusions	Two out of 31 in-vivo samples (PDTALL19_aN1_AT_B and PDTALL11_aN1_4) were considered outliers and excluded from the statistical analysis of the proteomics data.
Replication	For in-vitro proteomics experiments, biologically independent replicates were carried out by harvesting cells on different days (different passage number).<br>For in-vivo proteomics experiments, biologically independent replicates were represented by different mice.<br>Downstream sample preparation was always carried out in parallel.
Randomization	Allocation of cell wells / cell flasks / mice to experimental or control groups was random.<br>Randomization in sample acquisition was not carried out to reduce the chances of human error. All samples from one replicate were analyzed first and then the next replicate.
Blinding	Blinding was not carried out, as human participants were not involved in the study. Double blinding would not have been possible to carry out, as the user performing the experiment was the same person acquiring and analyzing the data.

Reporting for specific materials, systems and methods

We require information from authors about some types of materials, experimental systems and methods used in many studies. Here, indicate whether each material, system or method listed is relevant to your study. If you are not sure if a list item applies to your research, read the appropriate section before selecting a response.

Materials & experimental systems

n/a	Involved in the study	
[ ]	[x]	Antibodies
[ ]	[x]	Eukaryotic cell lines
[x]	[ ]	Palaeontology and archaeology
[ ]	[x]	Animals and other organisms
[x]	[ ]	Human research participants
[x]	[ ]	Clinical data
[x]	[ ]	Dual use research of concern

Methods

n/a	Involved in the study	
[x]	[ ]	ChIP-seq
[ ]	[x]	Flow cytometry
[x]	[ ]	MRI-based neuroimaging

Antibodies

Antibodies used	Cleaved Notch1 (Val1744), Cell Signaling Cat#4147<br>Phospho-S6 Ribosomal Protein (Ser235/236), Cell Signaling Cat#4858<br>S6 Ribosomal Protein, Santa Cruz Biotechnology Cat#74459<br>Phospho-PKCδ (Thr505), Cell Signaling Cat#9374<br>Phospho-PKCδ/θ (Ser643/676), Cell Signaling Cat#9376<br>Phospho PKCδ (Ser299), Abcam Cat#133456<br>PKCδ, Cell Signaling Cat#9616<br>PTEN, Cell Signaling Cat#9188<br>Phospho-Akt (Ser473), Cell Signaling Cat#4060

	Phospho-Akt (Thr 308), Cell Signaling Cat#4056<br>Akt, Cell Signaling Cat#9272<br>c-Myc, Cell Signaling Cat#5605<br>γ-Tubulin, Sigma Aldrich Cat#5326<br>Vinculin, Sigma Aldrich Cat#V9264<br>β-Actin, Santa Cruz Biotechnology Cat#47778<br>Cleaved caspase-3, Cell Signaling Cat#9664<br>Cleaved PARP, Cell Signaling Cat#5625<br>Anti-rabbit HRP conjugated secondary antibody, Jackson Immunoresearch 111-036-045<br>Anti-mouse HRP conjugated secondary antibody, Jackson Immunoresearch 115-036-062
Validation	All antibodies used were commercial and validated by supplier.<br>Western blotting: all antibodies used showed a specific band at the expected MW according to the protein marker.<br>Flow cytometry: all antibodies were validated by staining the cells with the isotype control antibody.<br>Cell cycle analysis: unstained cells were used as negative control.

Eukaryotic cell lines

Policy information about cell lines

Cell line source(s)	The human cell lines JURKAT (Clone E6-1) and MOLT-3 were purchased from ATCC. The human cell lines DND-41, HPB-ALL, ALL-SIL and PEER were purchased from DSMZ.
Authentication	Cell lines were authenticated by STR profiling using the ATCC authentication service according to manufacturers' instructions.
Mycoplasma contamination	All cell lines tested negative for mycoplasma contamination, evaluated using the EZ PCR Mycoplasma detection Kit (Biological Industries).
Commonly misidentified lines<br>(See ICLAC register)	No commonly misidentified cell lines were used in the study.

Animals and other organisms

Policy information about studies involving animals; ARRIVE guidelines recommended for reporting animal research

Laboratory animals	6-8 weeks old female NOD/SCID (NOD.CB17-Prkdcscid/NCrCrl) mice, purchased from Charles River Laboratories (Wilmington, MA, USA).
Wild animals	No wild animals were used in the study.
Field-collected samples	No field collected samples were used in the study.
Ethics oversight	Procedures involving animals conformed current laws and policies (EEC Council Directive 86/609, OJ L 358, 12/12 1987) and were authorized by the Italian Ministry of Health (894/2016-PR).

Note that full information on the approval of the study protocol must also be provided in the manuscript.

Flow Cytometry

Plots

Confirm that:

- [x] The axis labels state the marker and fluorochrome used (e.g. CD4-FITC).
- [x] The axis scales are clearly visible. Include numbers along axes only for bottom left plot of group (a 'group' is an analysis of identical markers).
- [x] All plots are contour plots with outliers or pseudocolor plots.
- [x] A numerical value for number of cells or percentage (with statistics) is provided.

Methodology

Sample preparation	Flow cytometry analysis of T-ALL PDX cells<br>Leukemic cells were collected from peripheral blood, spleen and BM of control and treated mice. Fluorescein isothiocyanate-labeled mAb against CD5 and phycoerythrin-Cy5-labeled mAb against CD7 (Coulter) were used for the detection of T-ALL cells in mouse samples. Samples were analyzed on the BD FACSCelesta flow cytometer. BD FACSDiva software was used for data acquisition and analysis.<br>Cell cycle analysis<br>1-2 x 10^6 cells were stained with the ghost dye violet 450 (Tonbo Biosciences), then fixed and permeabilized by using the Foxp3/Transcription Factor Staining Buffer Set (eBioscience), according to the manufacturer's instructions. DNA was stained by using a solution of 7-Aminoactinomycin D (7-AAD; Sigma-Aldrich), Ribonuclease A from bovine pancreas (Sigma-Aldrich) and PBS (Gibco). Cells were analyzed on a LSR Fortessa (BD Biosciences) flow cytometer; raw data were acquired through the

	BD FACSDiva software and analyzed by using the ModFit LT software.
Instrument	LSR Fortessa, BD FACSCelesta
Software	BD FACSDiva software, ModFit LT V.4.1.7
Cell population abundance	Leukemic cells in blood were measured in the lymphocyte gate in the FSCA vs SSC-A plot (25% population abundance as average value). This value is adequate for further analysis, considering a 62% average value of fragments in the population analyzed.
Gating strategy	FSC-A/SSC-A (for both mice and cell cycle); FSC-W/FSC-H, FSC-A/405D-A (for cell cycle only)

☒ Tick this box to confirm that a figure exemplifying the gating strategy is provided in the Supplementary Information.
