# Supplementary material for: Proteomics of resistance to Notch1 inhibition in acute lymphoblastic leukemia reveals targetable kinase signatures
Source: Nat Commun. 2021 May 4;12:2507. doi: 10.1038/s41467-021-22787-9 (PMC8097059; doi:10.1038/s41467-021-22787-9)

FIG. 6c left

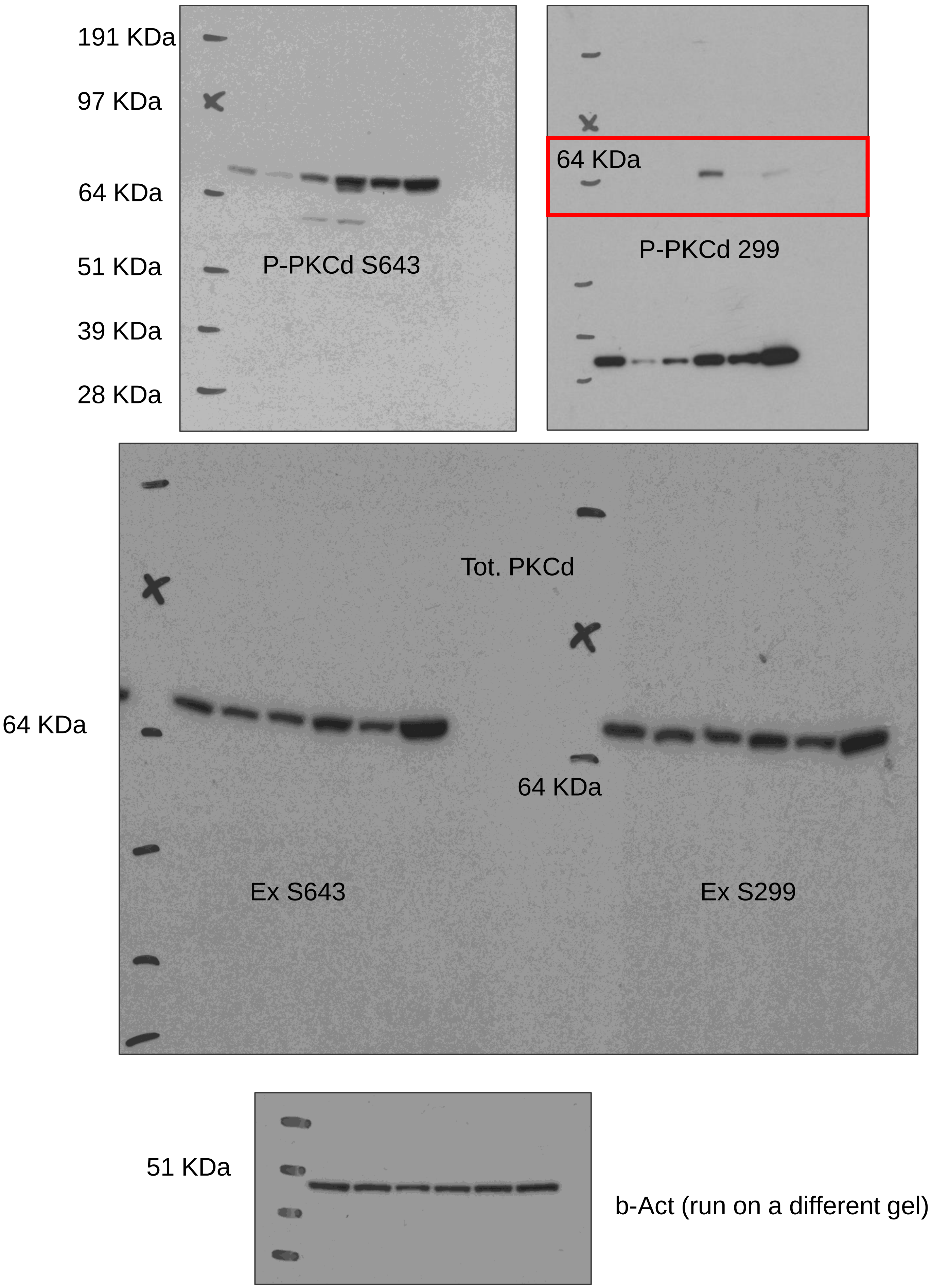

191 KDa
97 KDa
64 KDa
51 KDa
39 KDa
28 KDa
P-PKCd S643
64 KDa
P-PKCd 299
Tot. PKCd
64 KDa
64 KDa
Ex S643
Ex S299
51 KDa
b-Act (run on a different gel)

FIG. 6c right

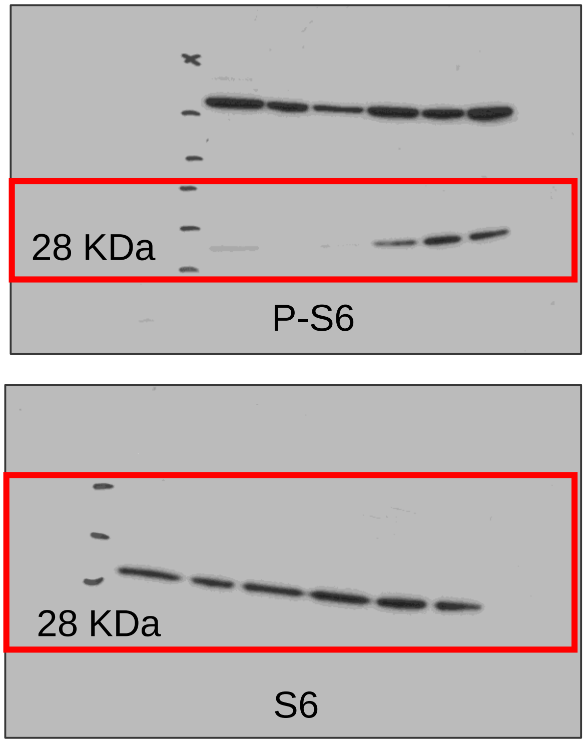

FIG. 6e

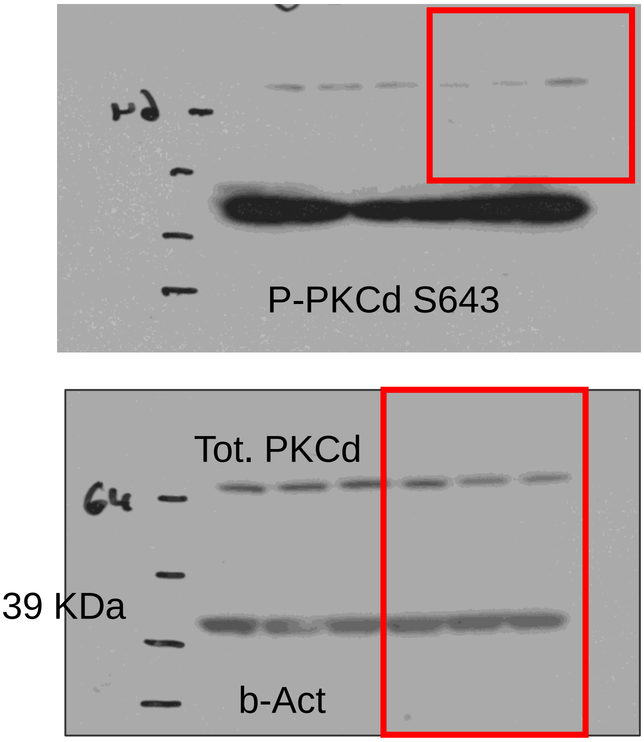

FIG. 6e

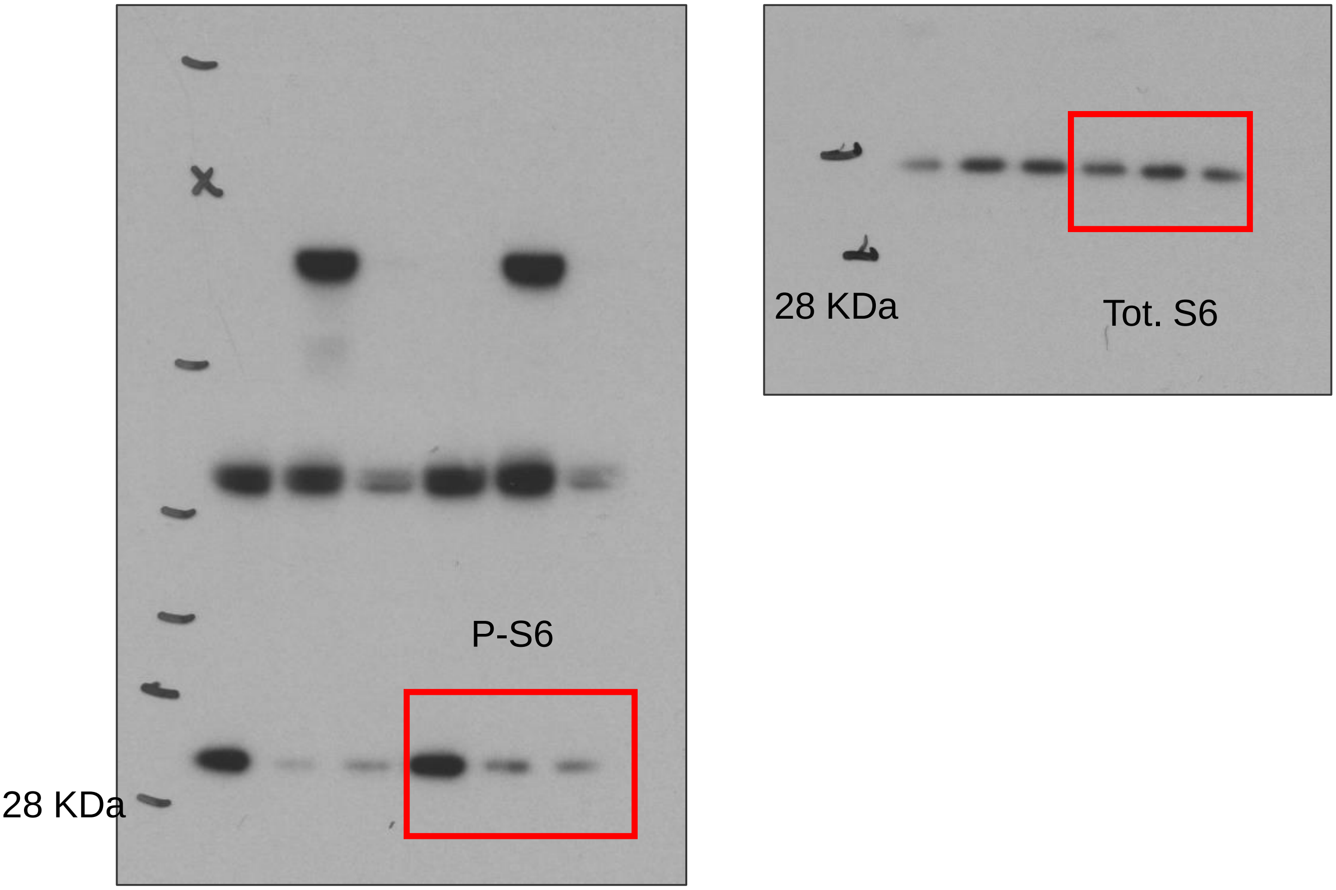

FIG. 6f

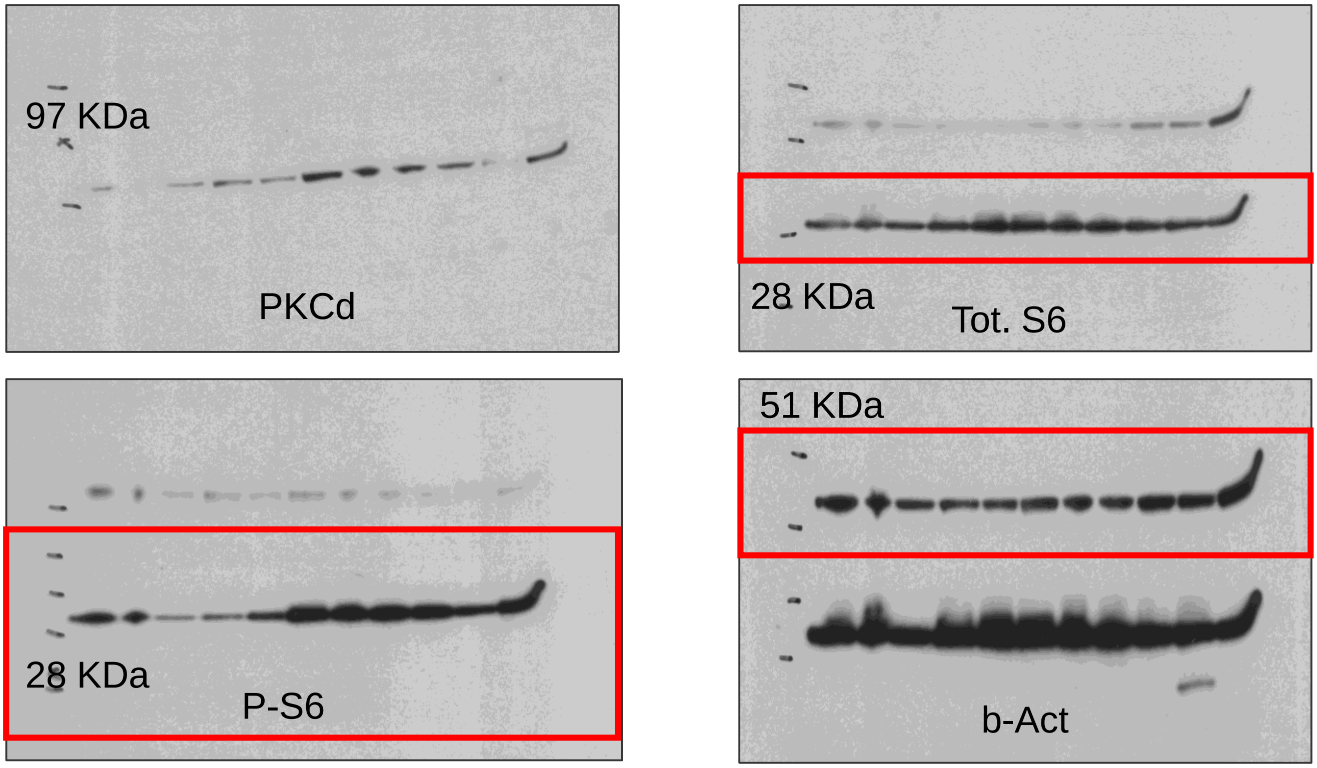

FIG. S6c

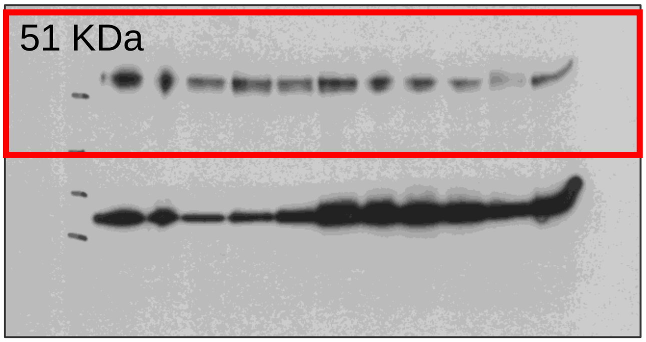

cMyc

FIG. 7a – Sensitive

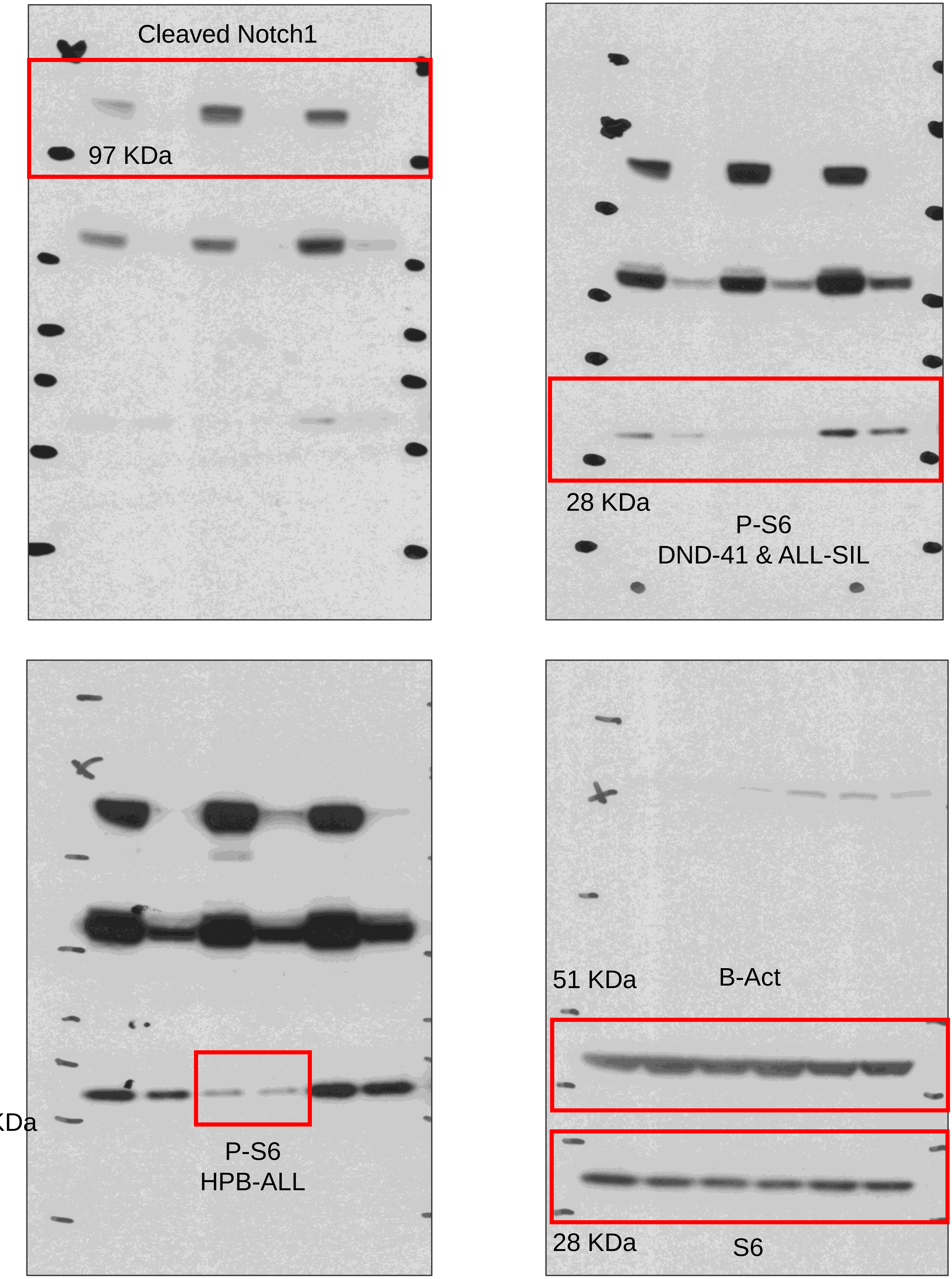

FIG. 7a – Resistant

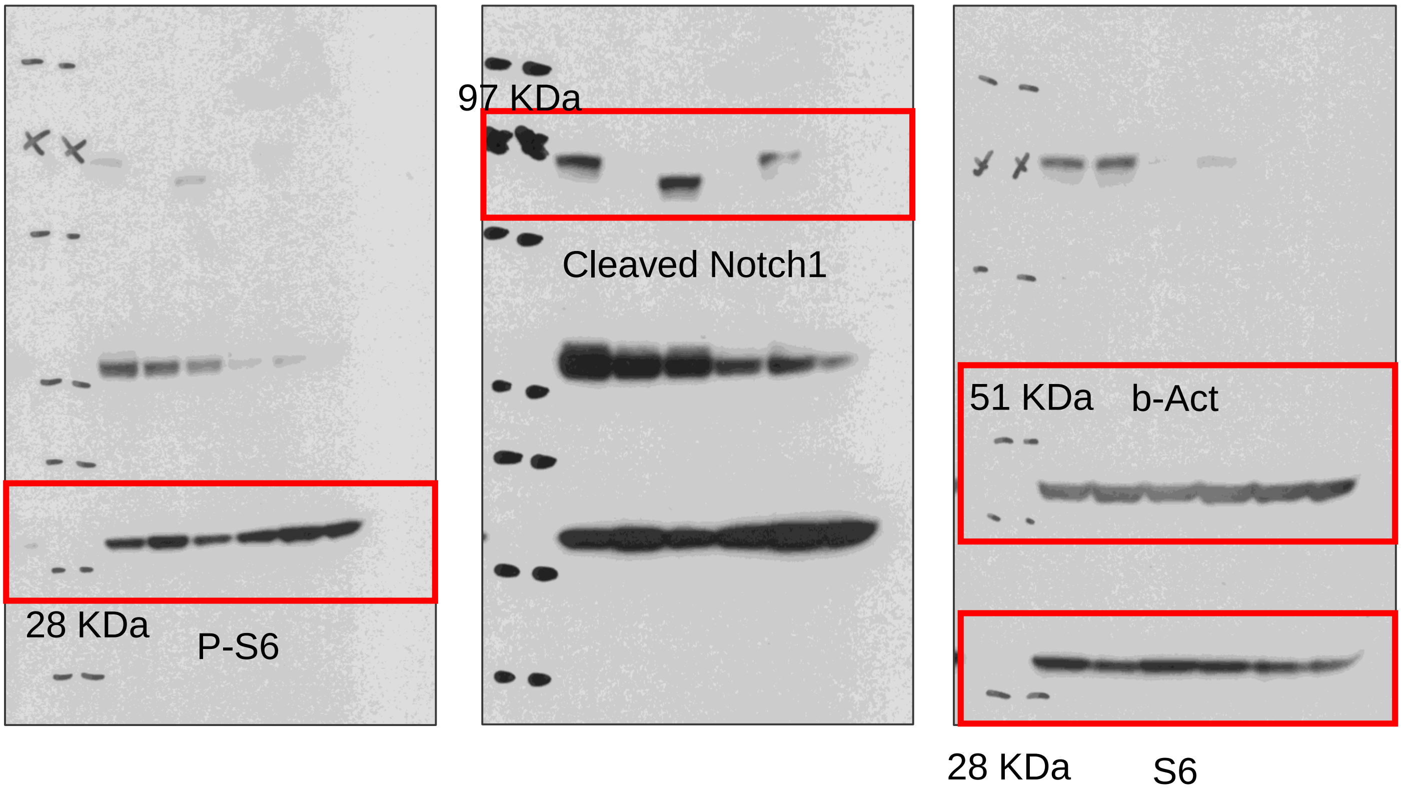

FIG. 7b JURKAT

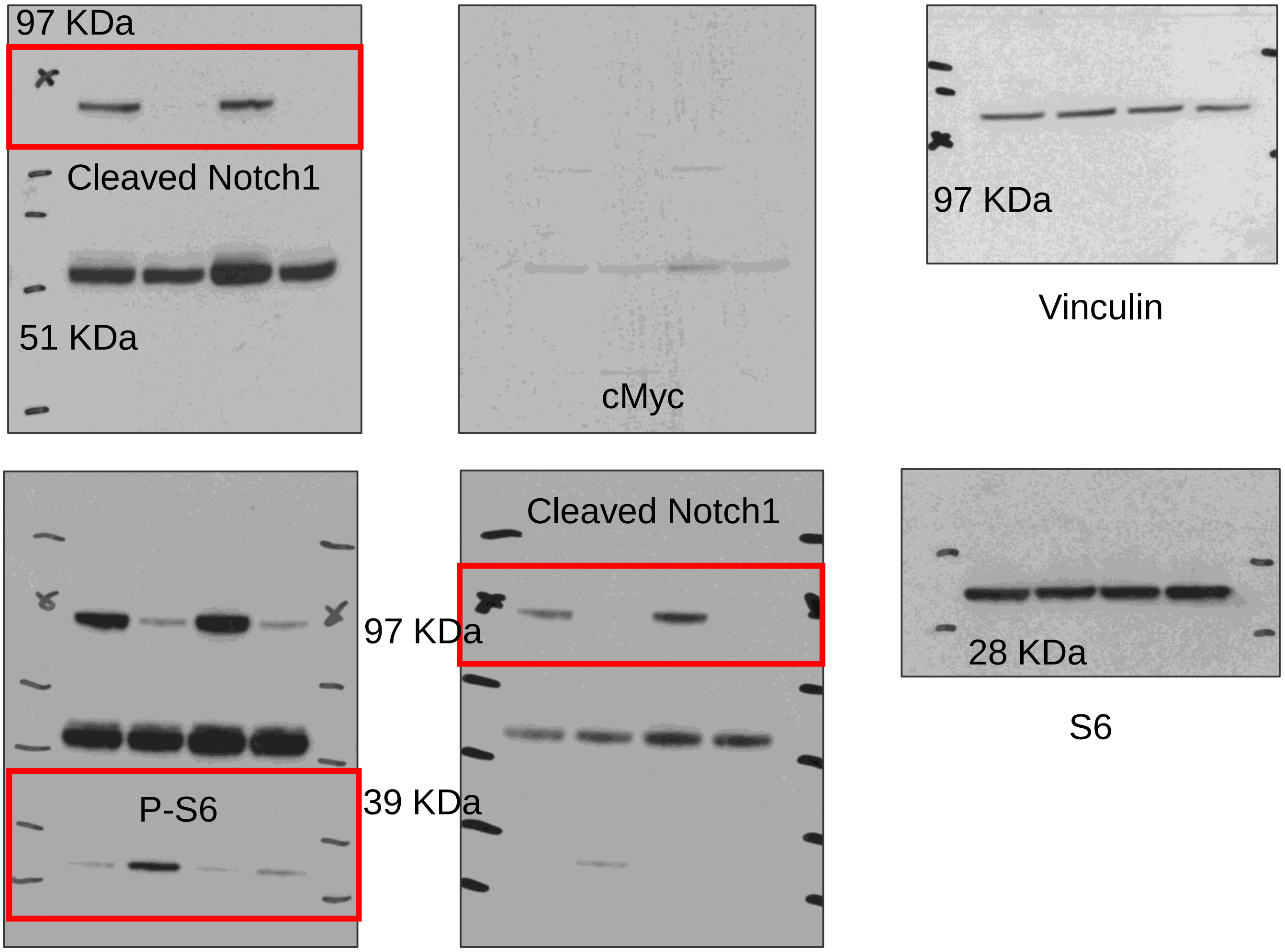

FIG. 7b PEER

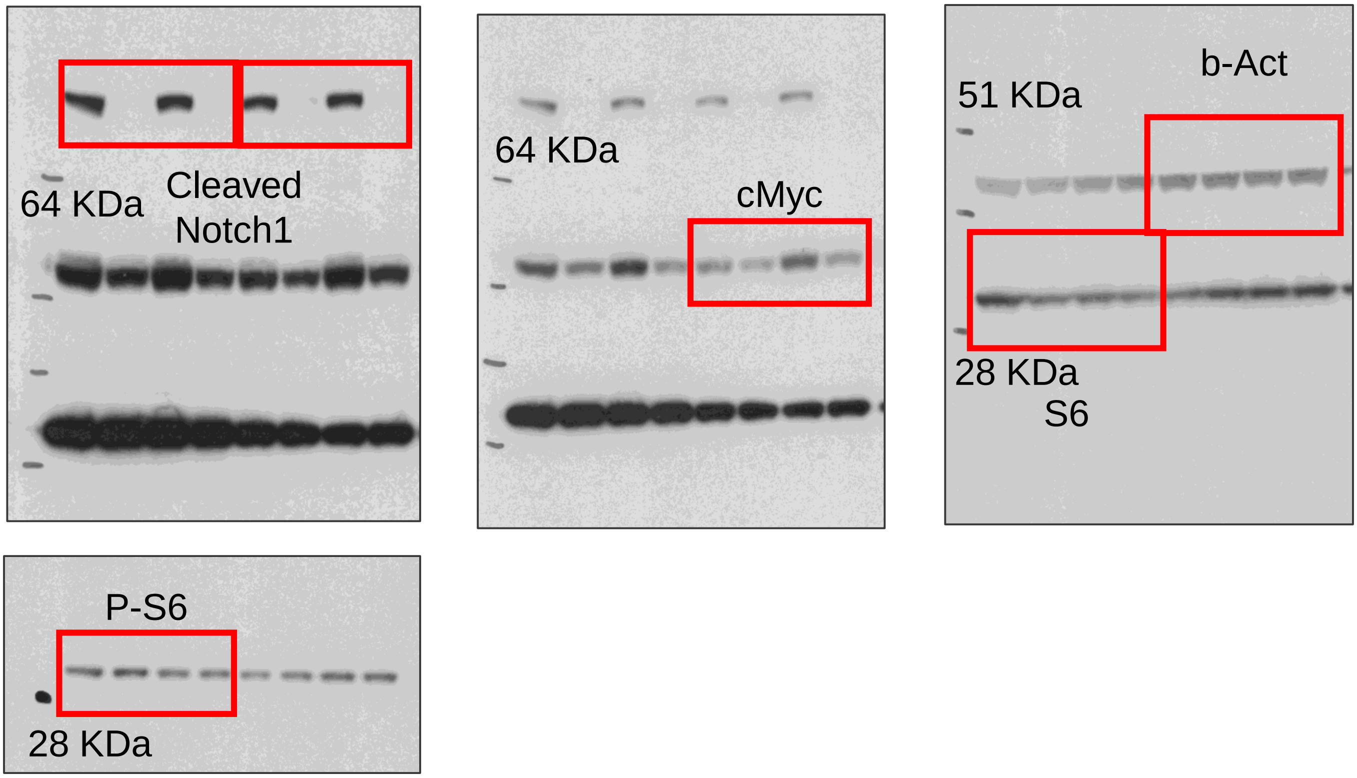

FIG. 7b MOLT-3

5-day time point

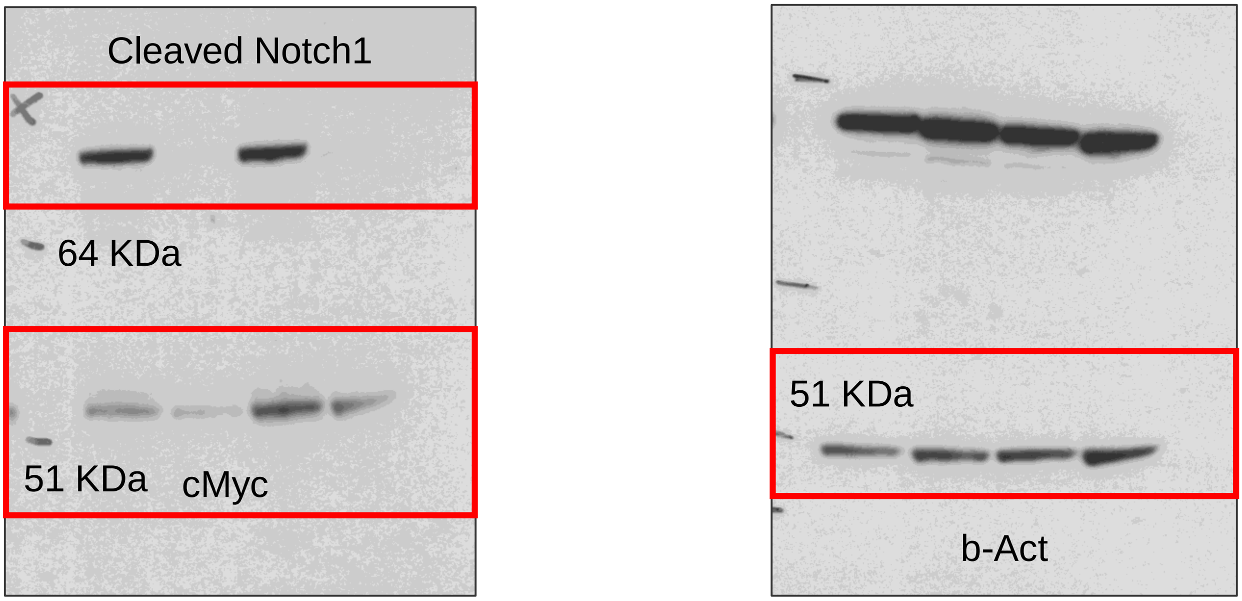

3-day time point

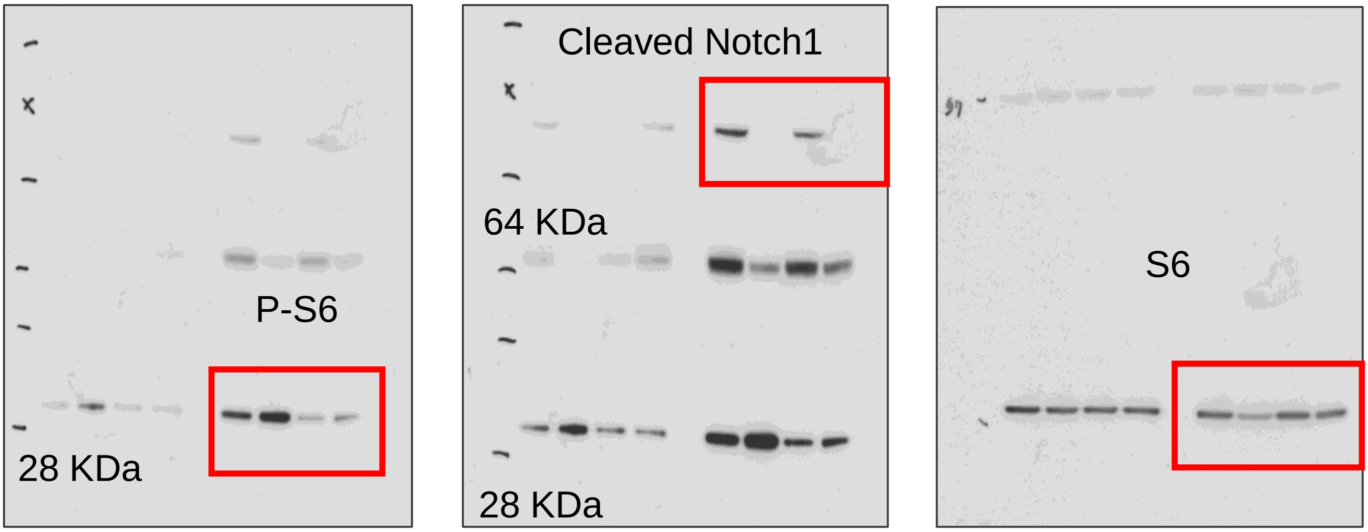

FIG. 7d

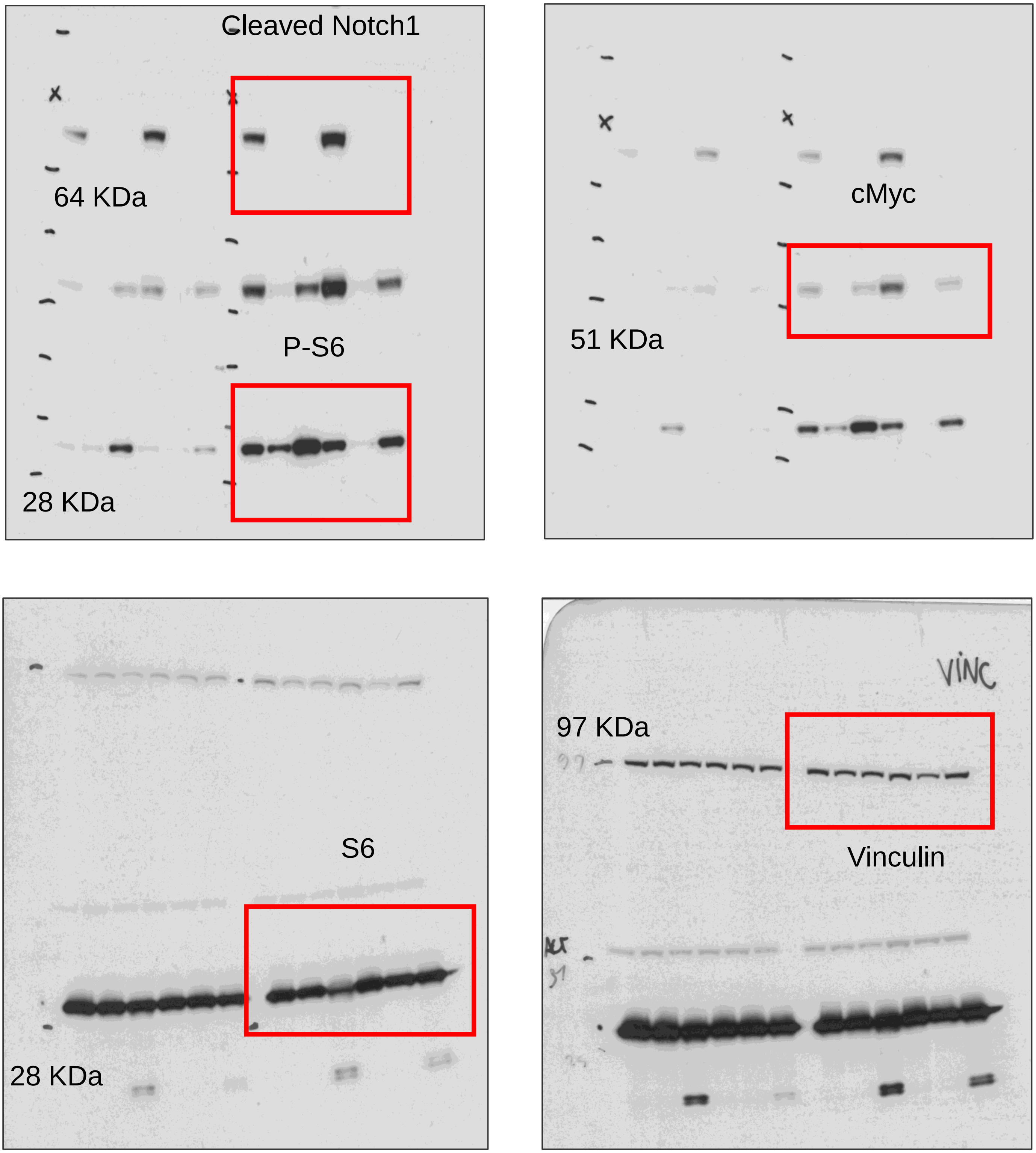

Cleaved Notch1
64 KDa
P-S6
28 KDa
cMyc
51 KDa
S6
28 KDa
VINC
97 KDa
Vinculin

DND-41

FIG. 7d

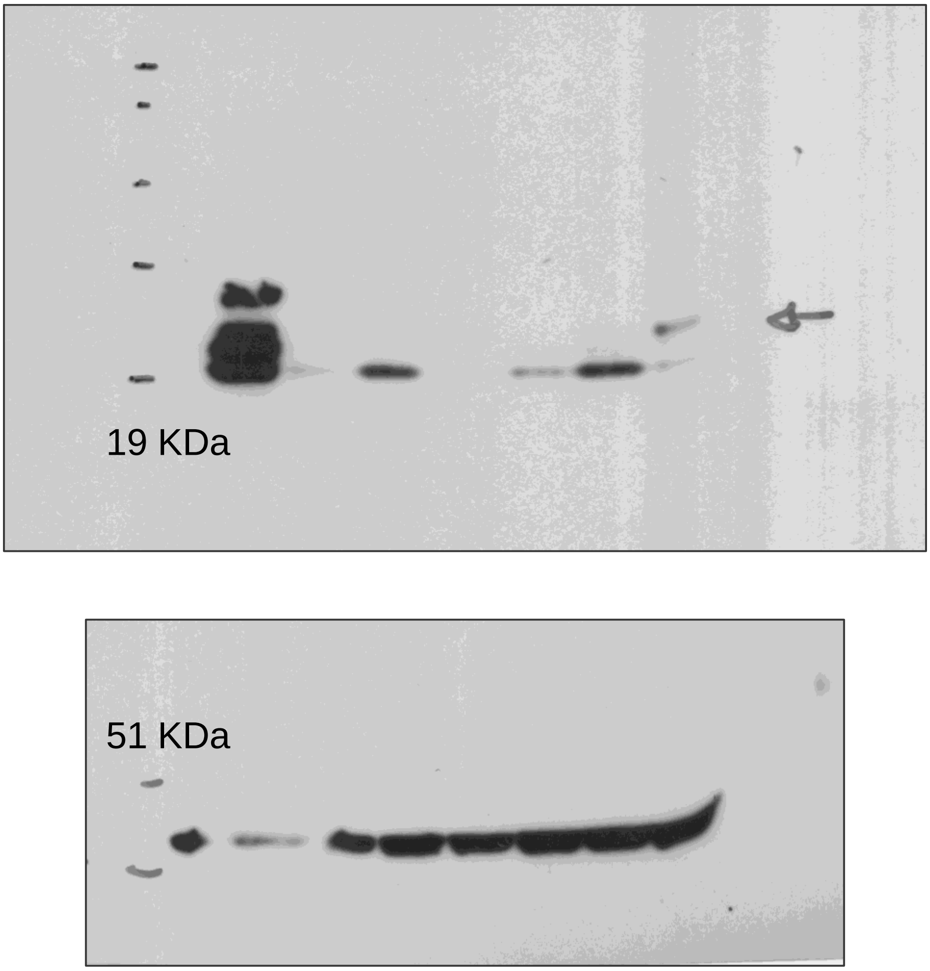

Cleaved caspase 3

β-Actin

FIG. 8e

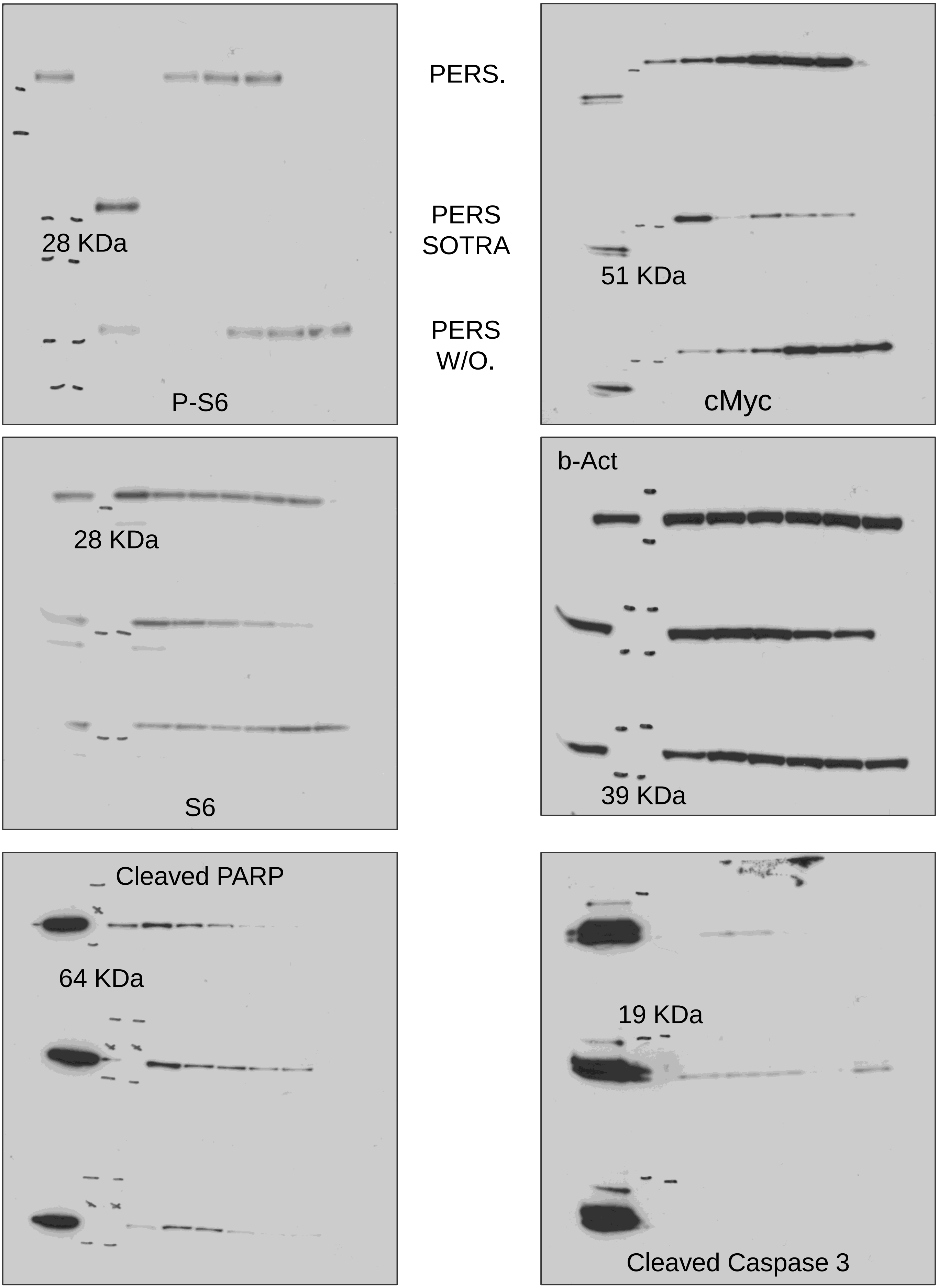
PERS.
PERS
SOTRA
PERS
W/O.
28 KDa
P-S6
51 KDa
cMyc
28 KDa
S6
b-Act
39 KDa
Cleaved PARP
64 KDa
19 KDa
Cleaved Caspase 3

FIG. S1b

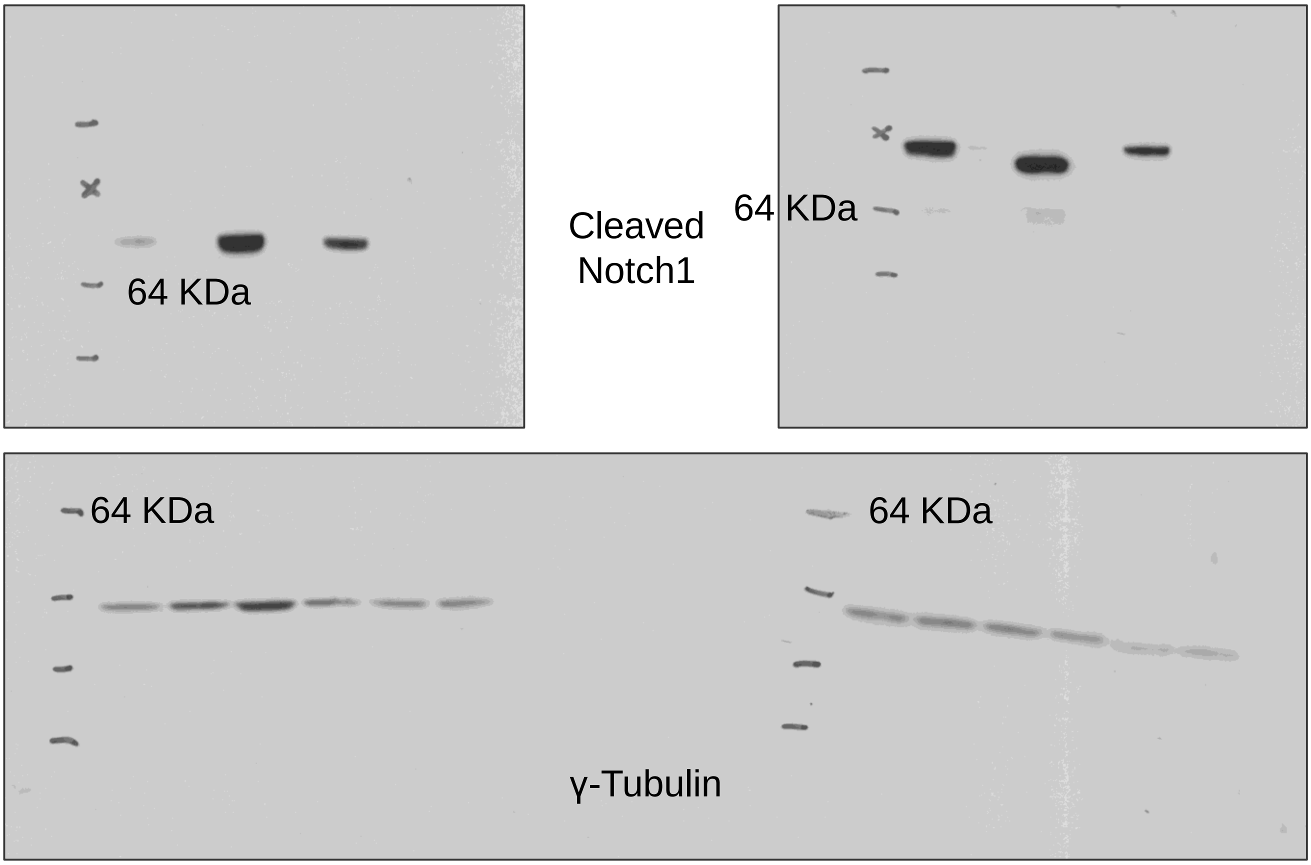

FIG. S2b

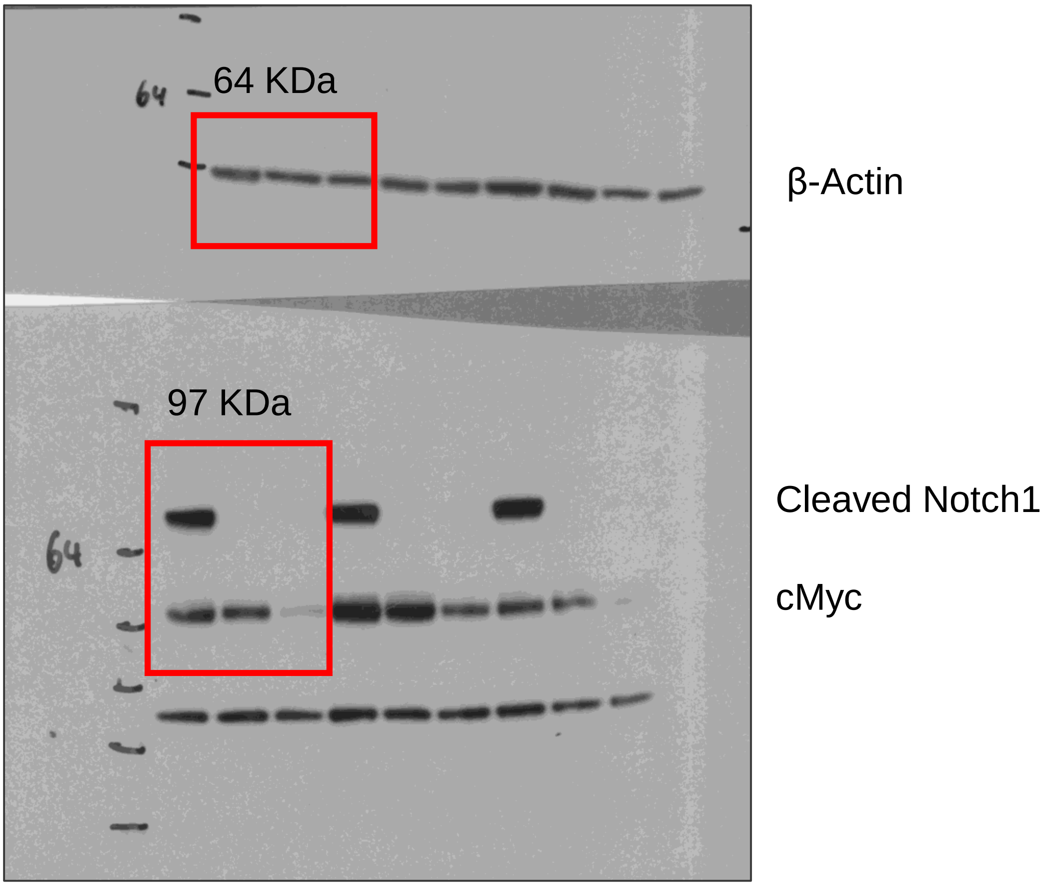

FIG. S6b & S7c

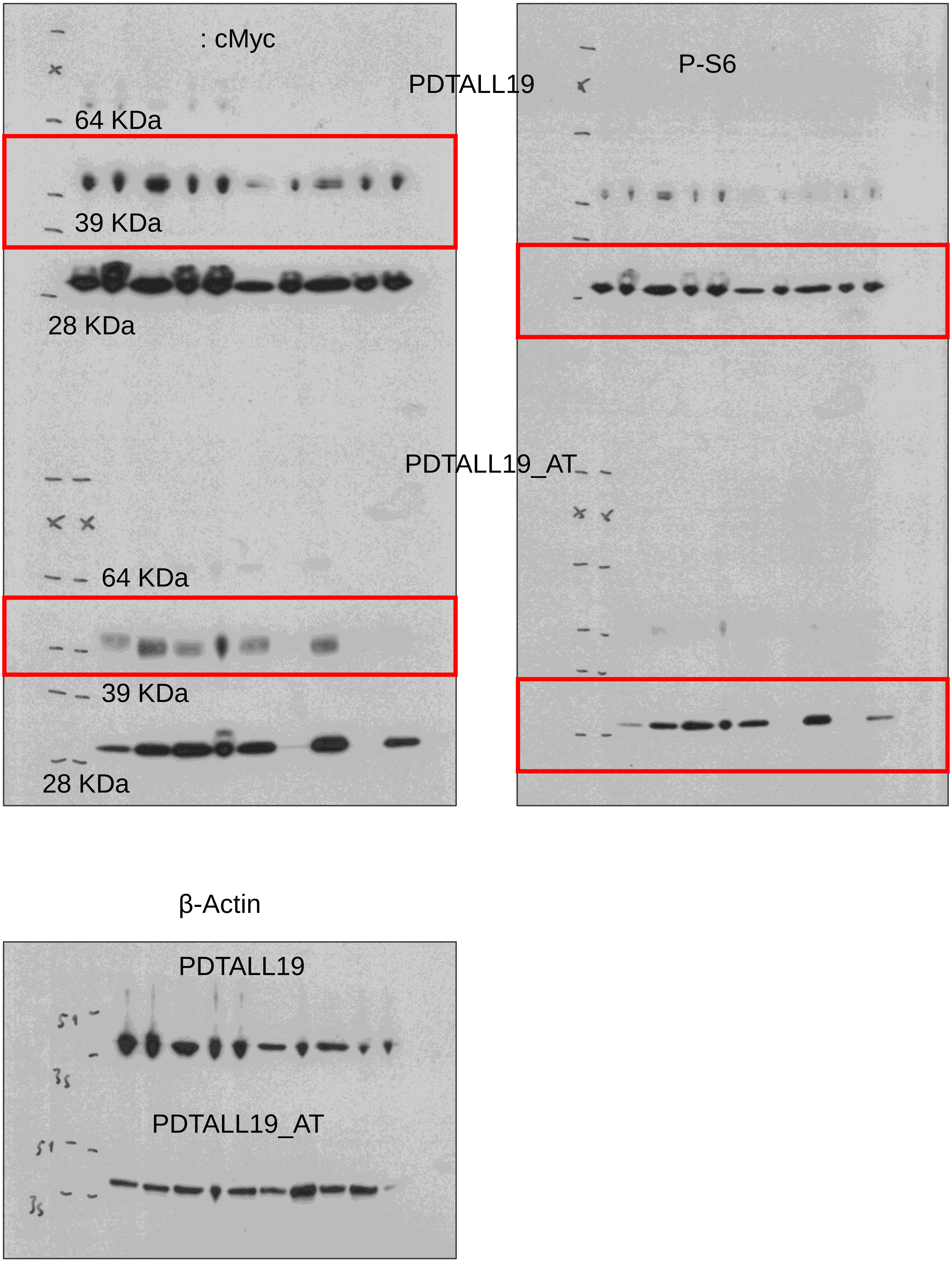
: cMyc
PDTALL19
64 KDa
39 KDa
28 KDa
PDTALL19_AT
64 KDa
39 KDa
28 KDa
P-S6
β-Actin
PDTALL19
PDTALL19_AT

FIG. S6b & S7c

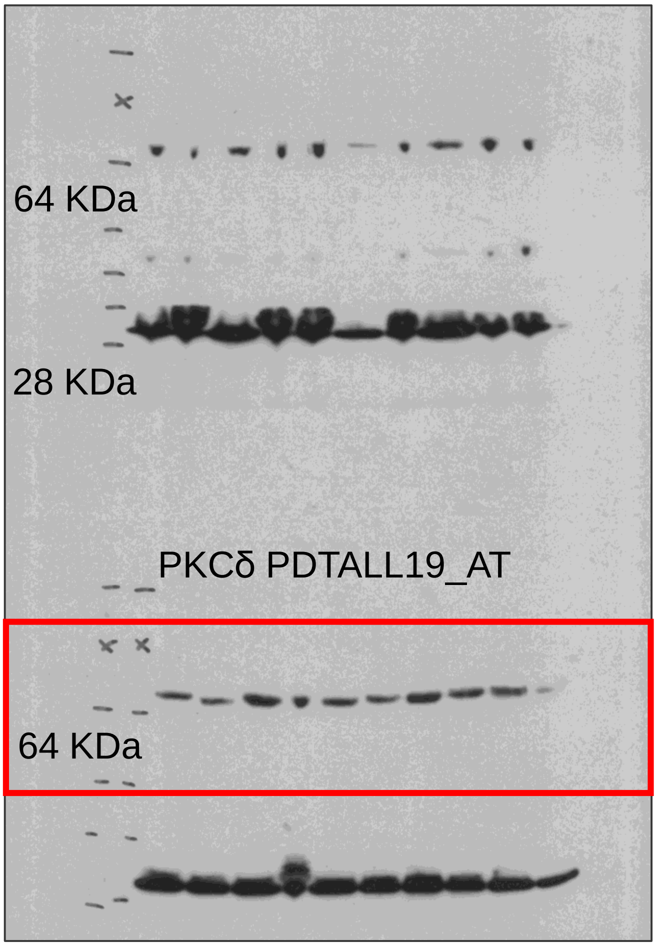

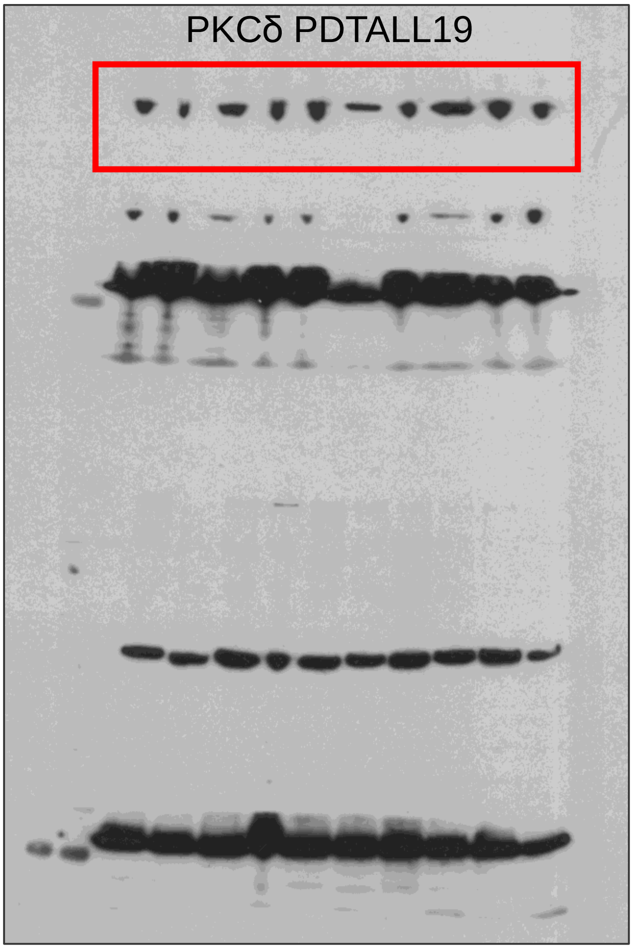

PDTALL19
S6

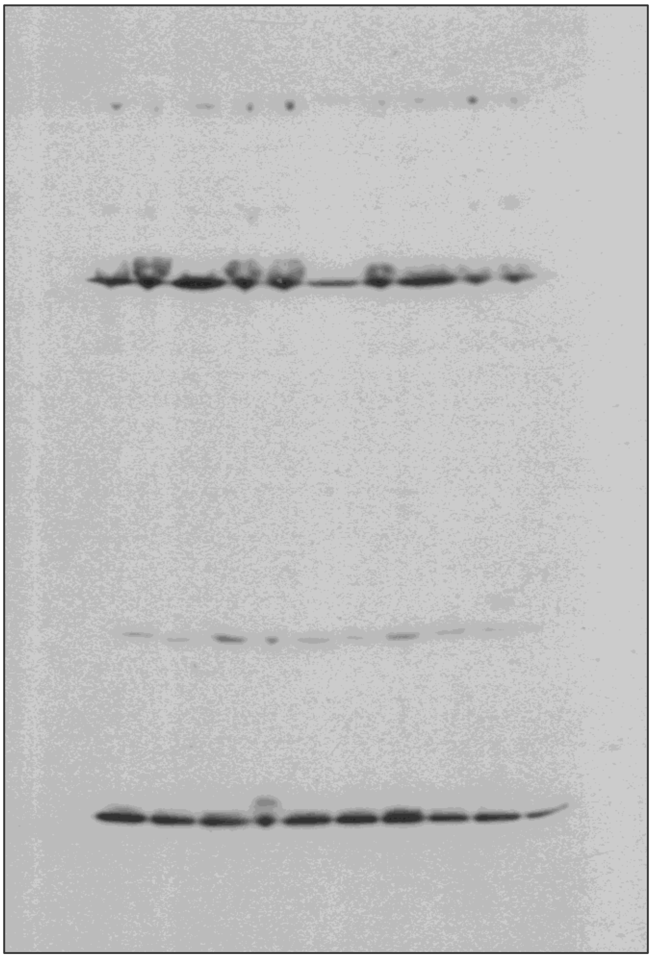

PDTALL19_AT
S6

FIG. S7a

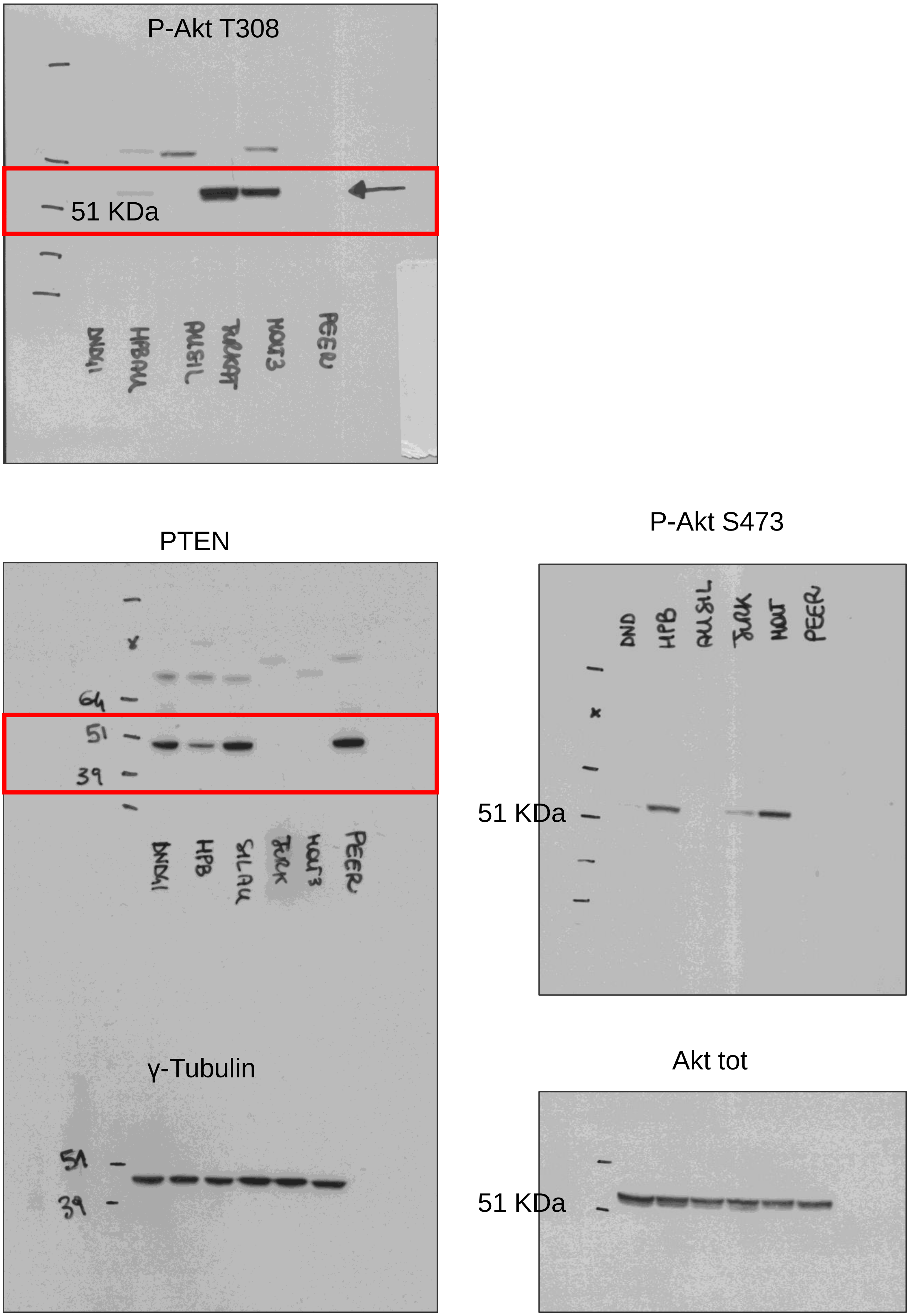

FIG. S7b

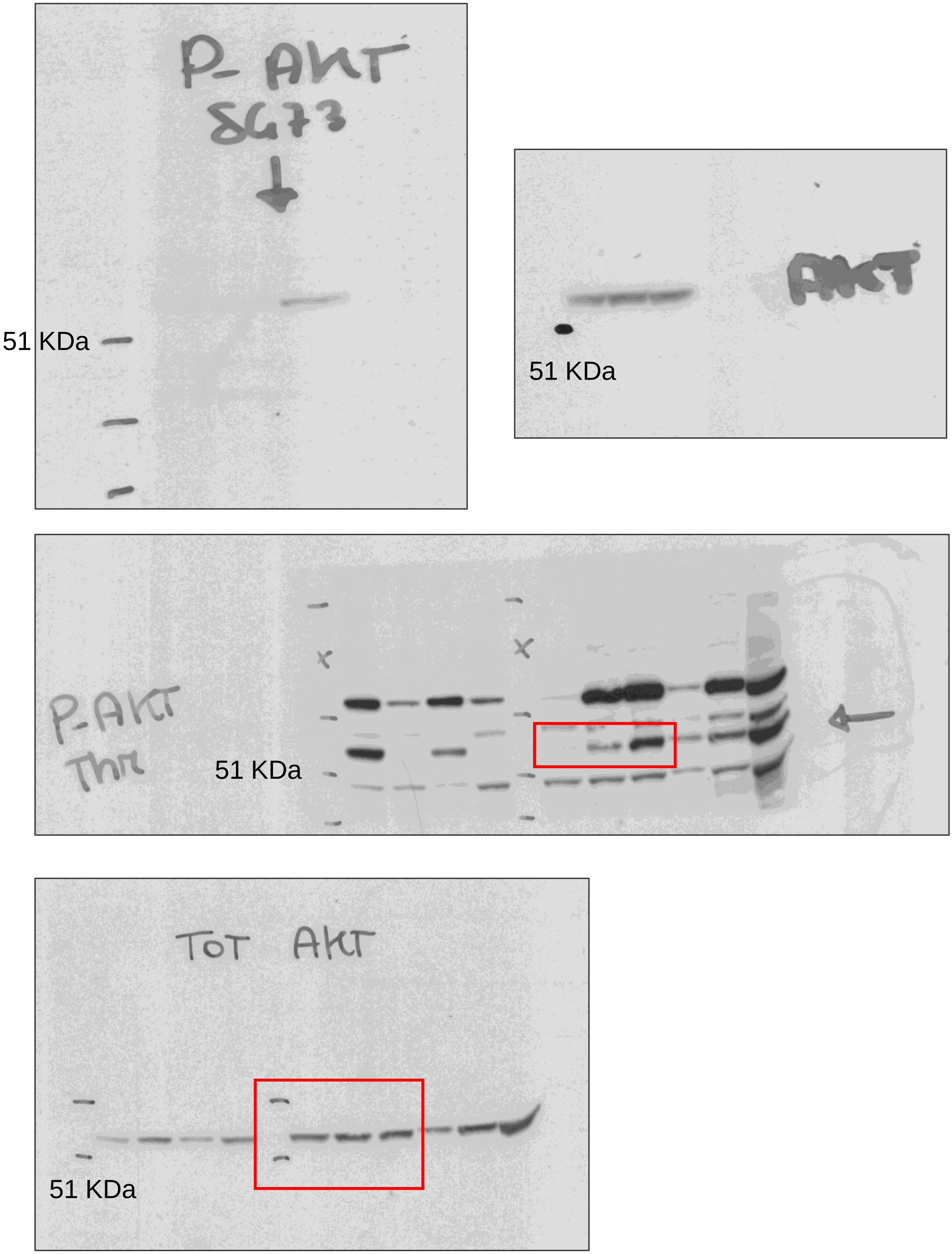

FIG. S7e

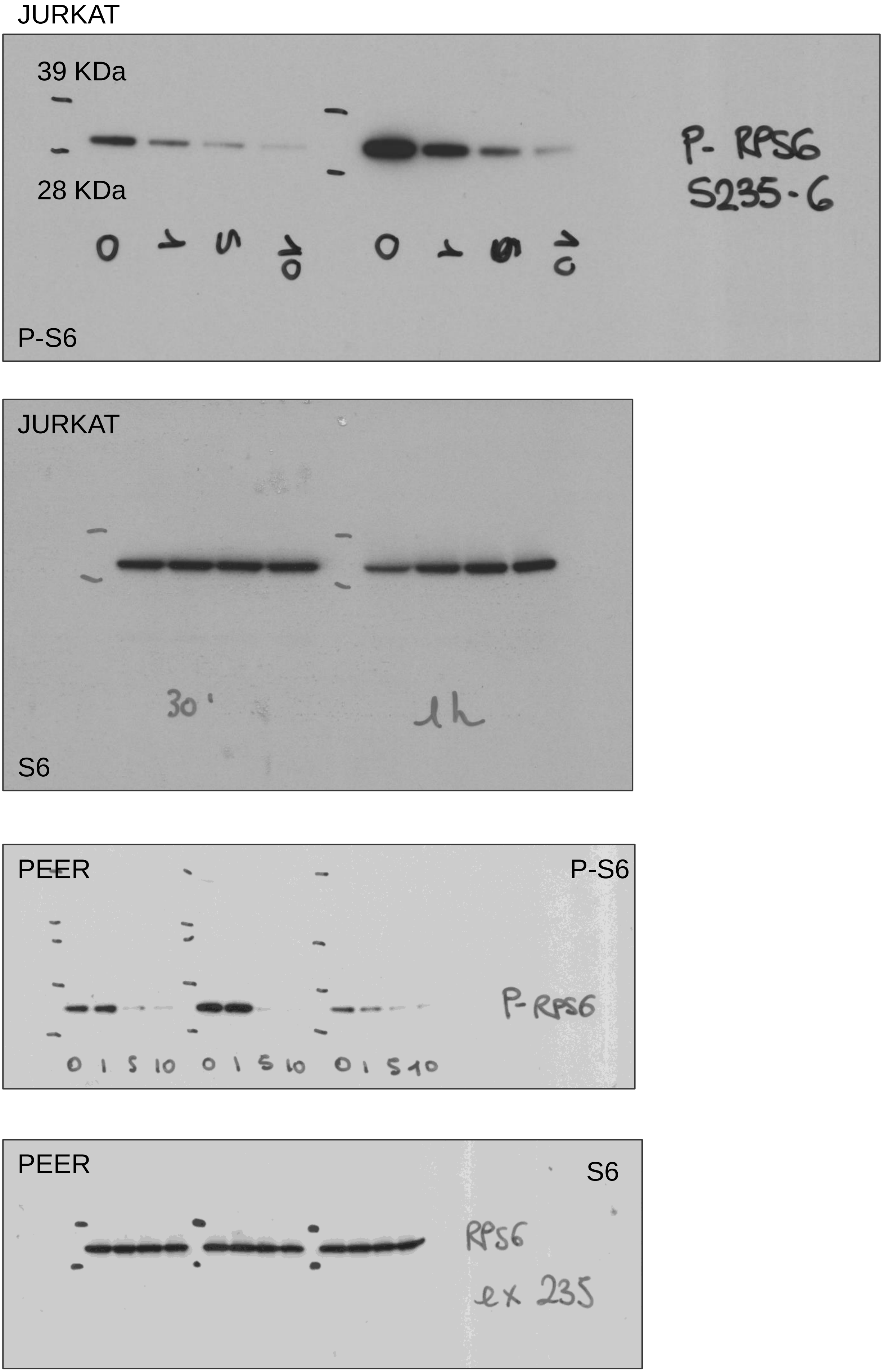
JURKAT
39 KDa
28 KDa
P- RPS6
S235-6
P-S6
JURKAT
30'
1h
S6
PEER
P-S6
P-RPS6
0 1 5 10 0 1 5 10 0 1 5 10
PEER
S6
RPS6
ex 235

FIG. S7e

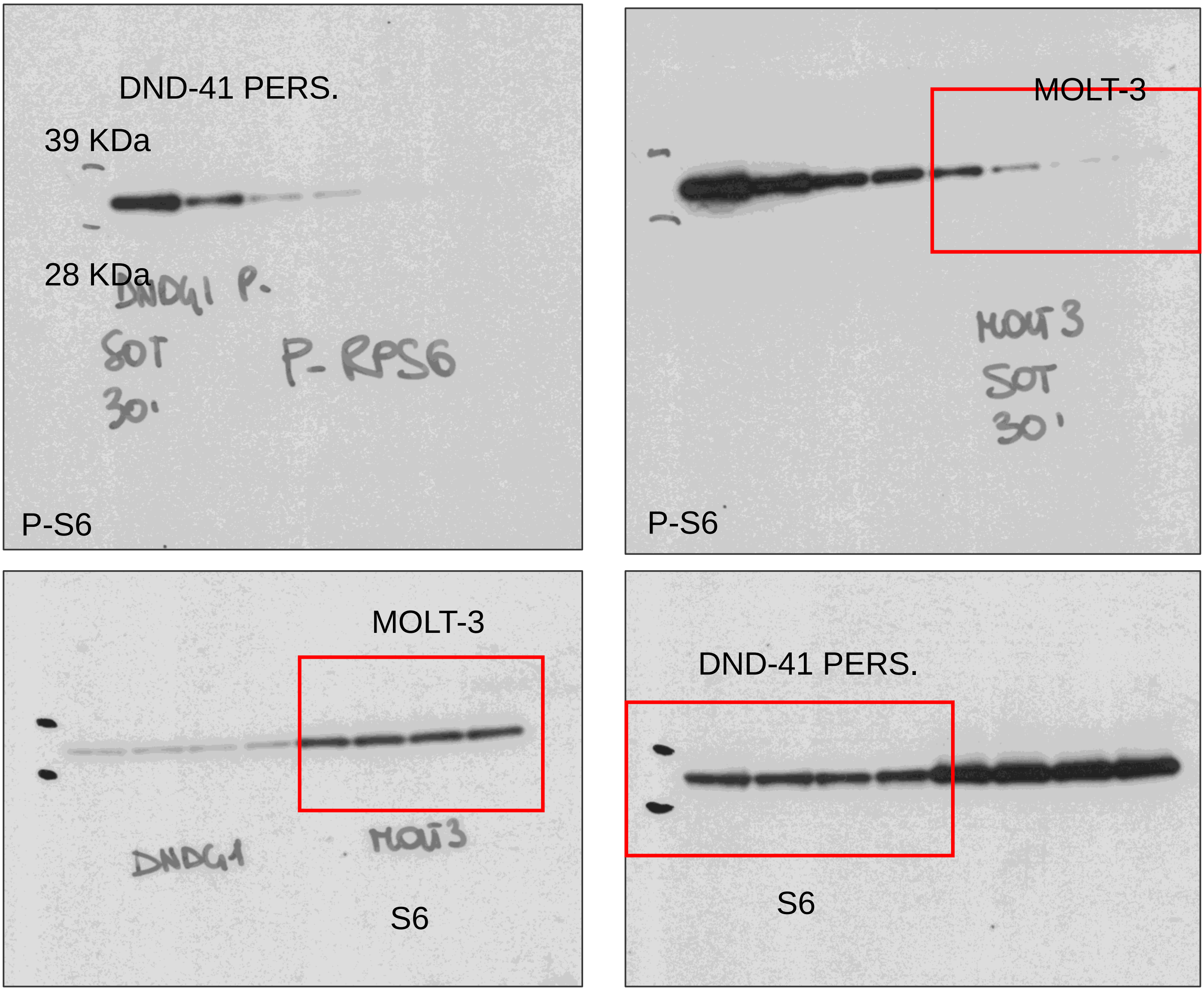
DND-41 PERS.
39 KDa
28 KDa
DND41 P.
SOT
30'
P-RPS6
P-S6
MOLT-3
MOLT3
SOT
30'
P-S6
MOLT-3
DND41
MOLT3
S6
DND-41 PERS.
S6

FIG. S7e

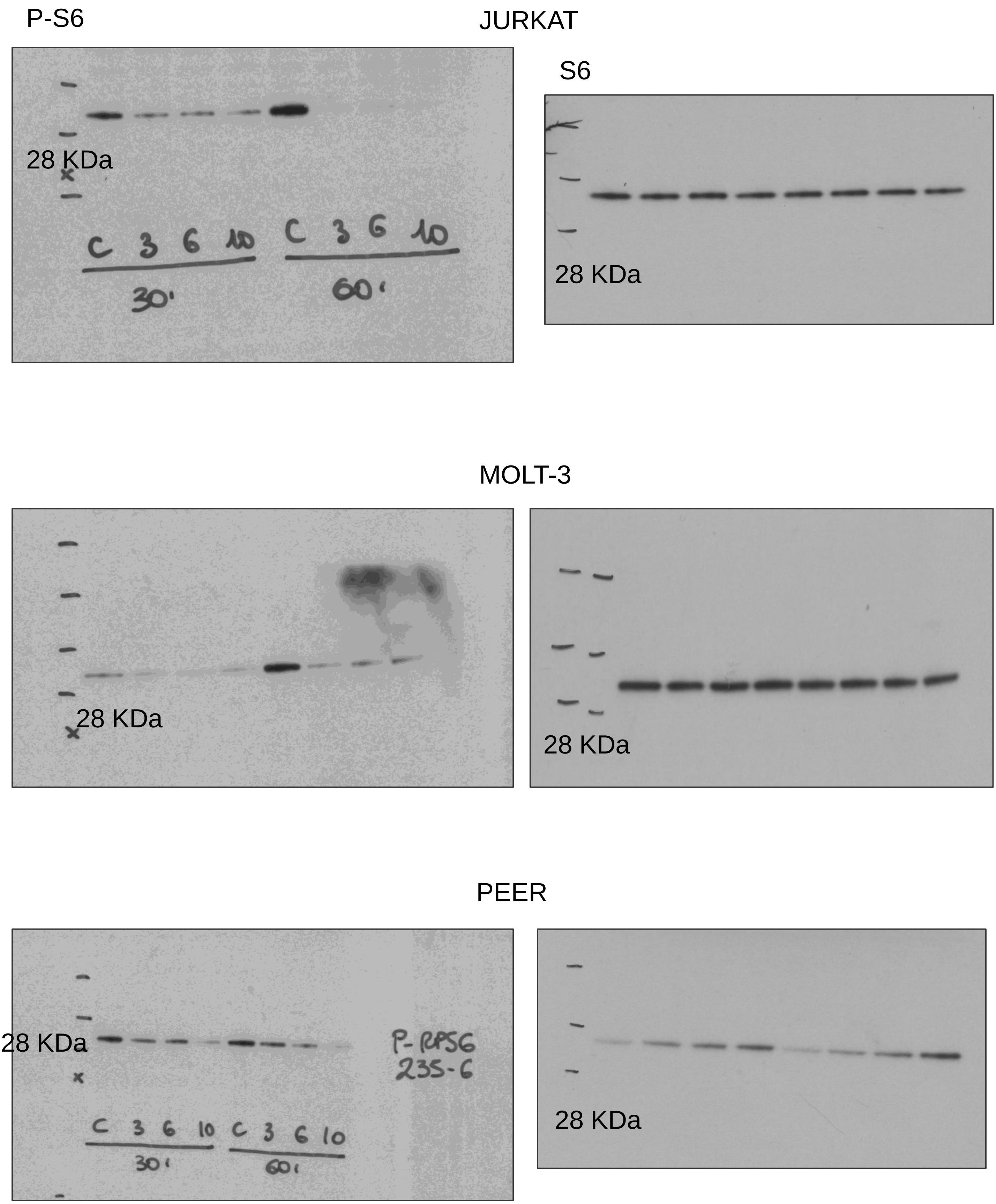
P-S6
JURKAT
S6
28 KDa
C 3 6 10 C 3 6 10
30'
60'
28 KDa
MOLT-3
28 KDa
28 KDa
PEER
28 KDa
P-RPS6
235-6
C 3 6 10 C 3 6 10
30'
60'
28 KDa

FIG. S7e

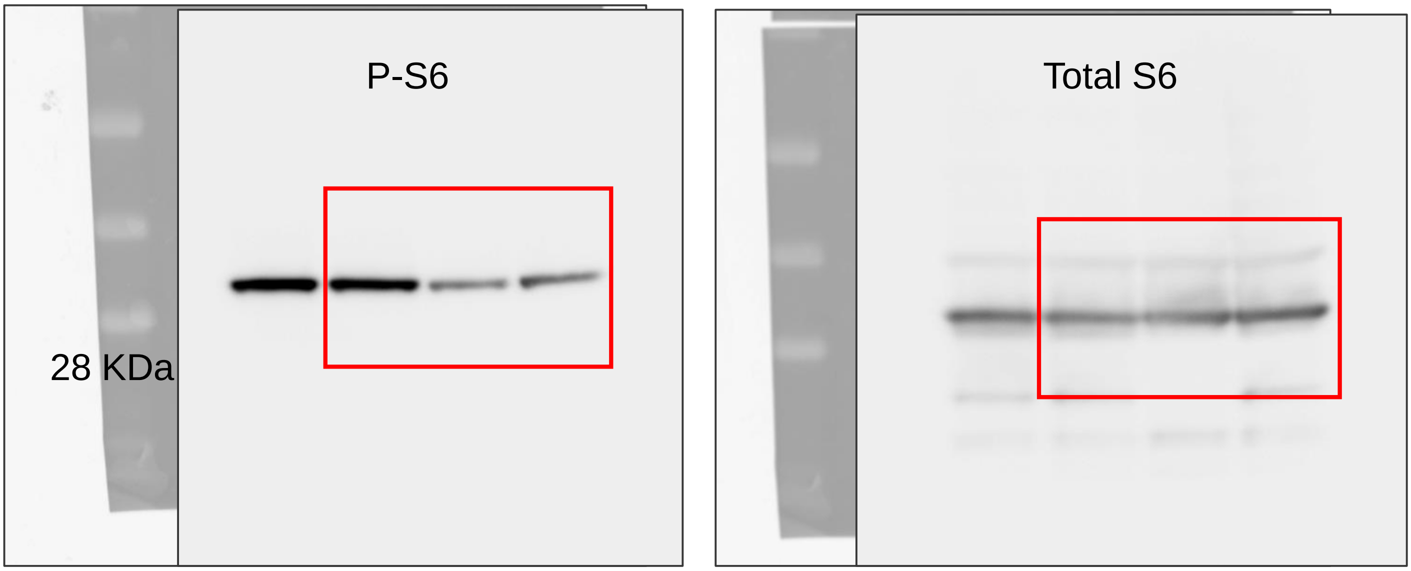

FIG. S7f

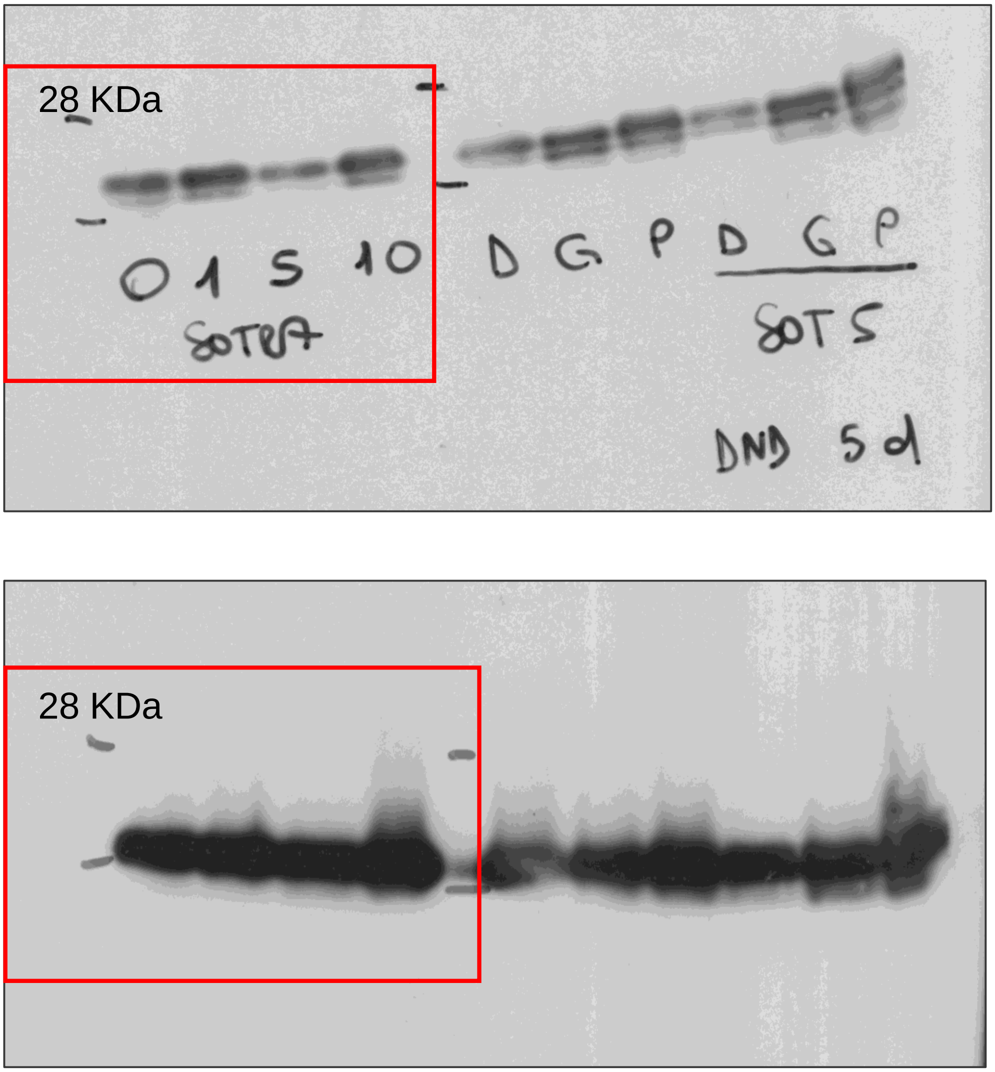

P-4E-BP1

4E-BP1

FIG. S8a

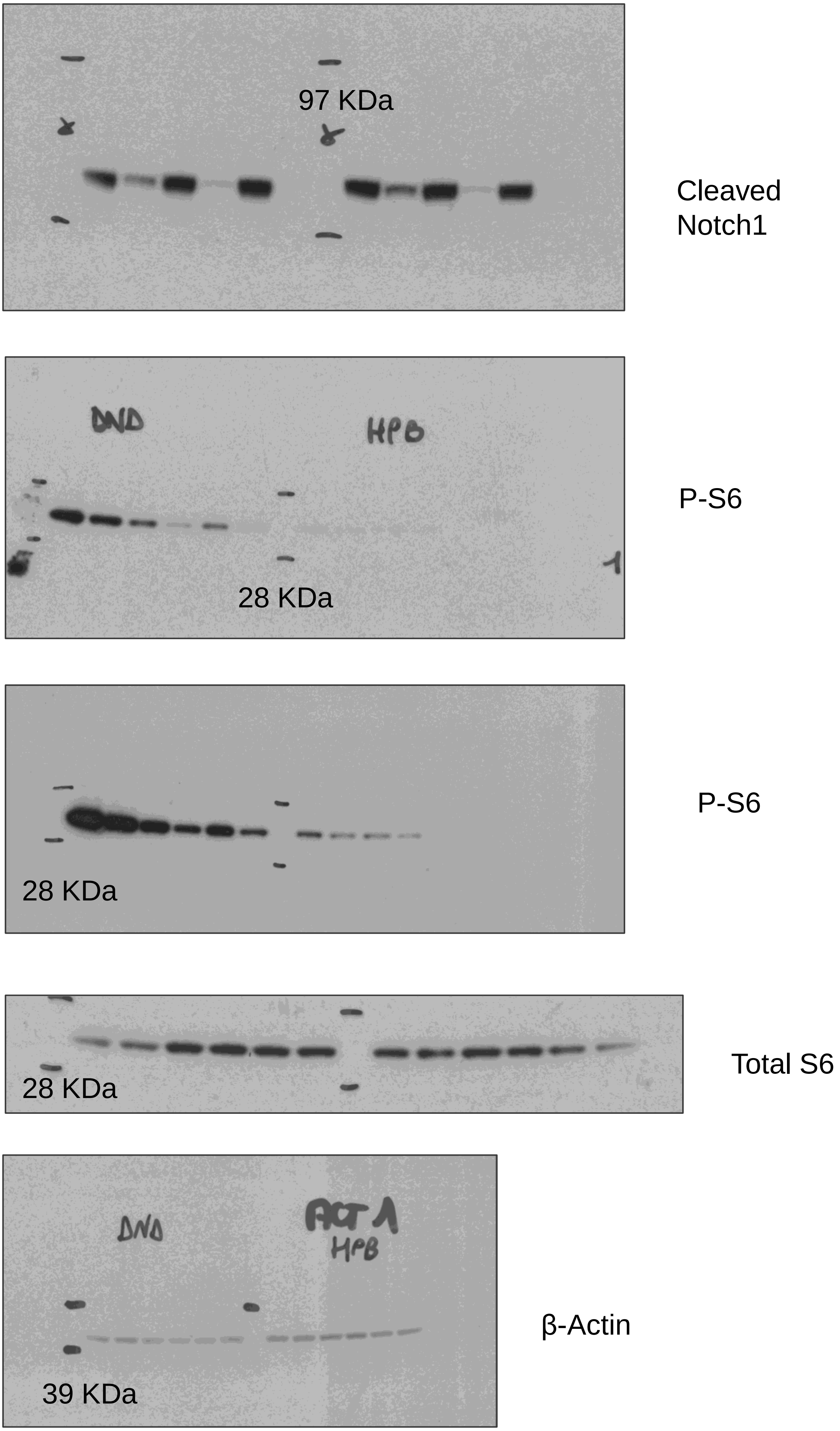
97 KDa
Cleaved Notch1
28 KDa
P-S6
28 KDa
P-S6
28 KDa
Total S6
39 KDa
β-Actin

FIG. S9f

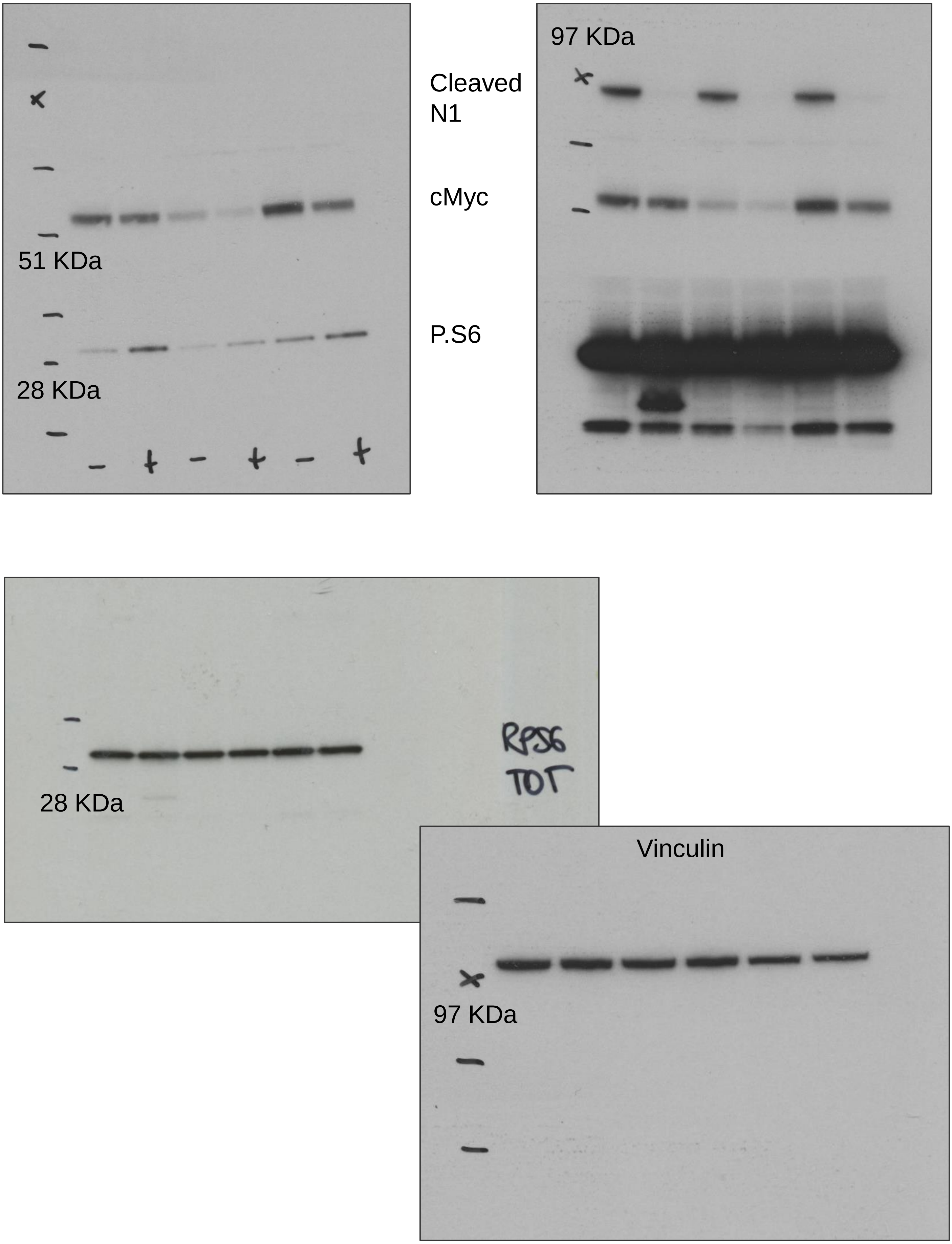
97 KDa
Cleaved
N1
cMyc
P.S6
51 KDa
28 KDa
- + - + - +
28 KDa
RPS6
TOT
Vinculin
97 KDa

FIG. S9h

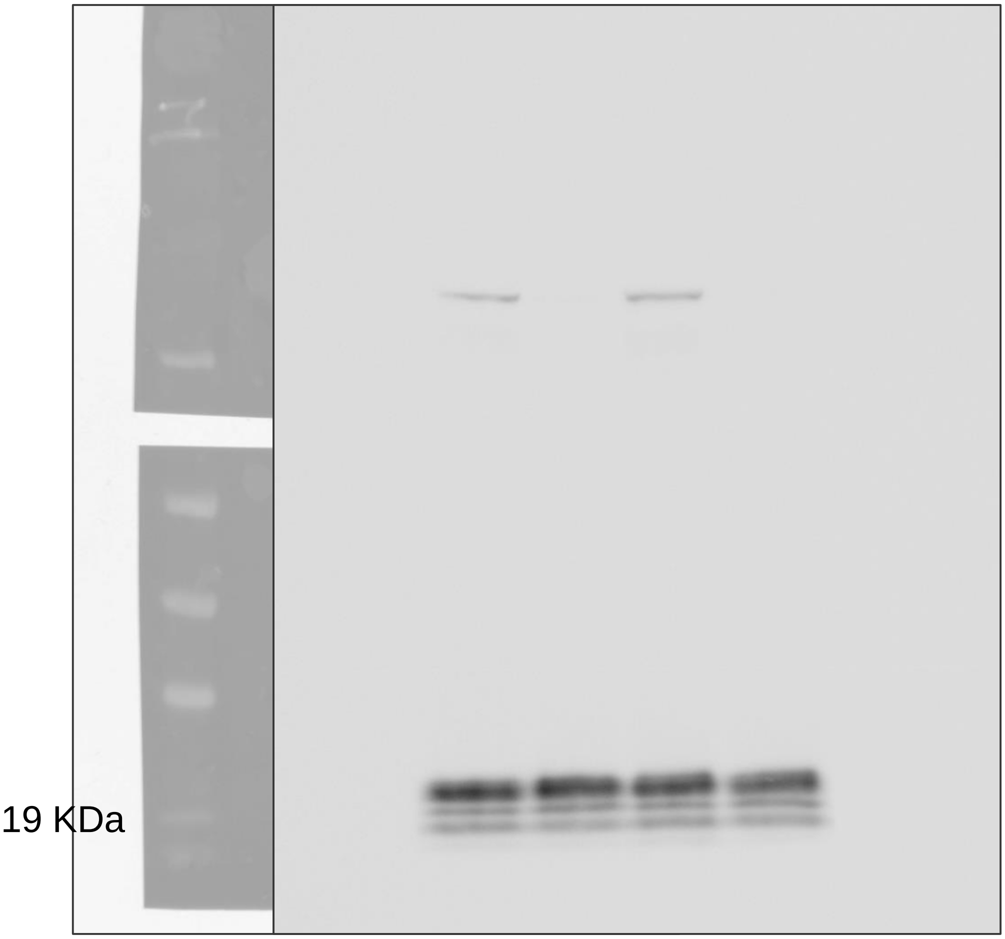

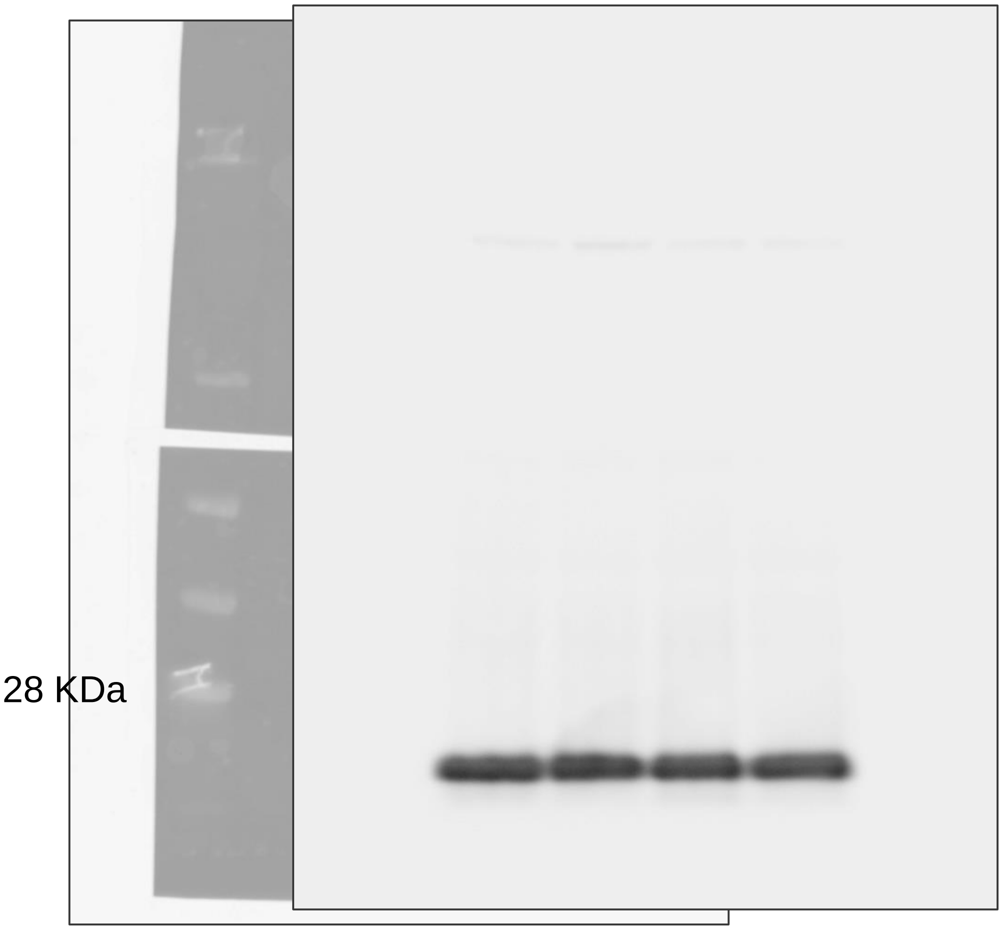

Supplement: Supplementary file 10 — Source Data [file 41467_2021_22787_MOESM10_ESM.zip › Source data/Uncropped blots.pdf]
